# A Sterically Encumbered Triarylborane Enabling CO_2_ Reduction to Formate by Electrons and Protons

**DOI:** 10.1021/acs.inorgchem.6c03816

**Published:** 2026-10-03

**Authors:** Felix Meyer, Ludwig Zapf, Eric Rivard, Inke Siewert

**Affiliations:** † Universität Göttingen, Institut für Anorganische Chemie, Tammannstr. 4, 37077 Göttingen, Germany; ‡ 3158 University of Alberta, Department of Chemistry, 11227 Saskatchewan Dr. NW, Edmonton, Alberta T6G 2G2, Canada

## Abstract

Borohydrides are widely used sources of hydride ions (H^–^), but their conventional synthesis from highly reducing metal hydrides is energetically demanding and undermines sustainable applications. Herein, we report a sterically encumbered thioether-substituted triaryl borane, 1,2-(^*t*^BuS)­C_6_H_4_(BMes_2_) (**3**) (Mes = 2,4,6-Me_3_C_6_H_2_), which is highly air- and moisture-stable. **3** exhibits a one-electron reduction at *E* ^0^ = −2.62 V vs Fc^+|0^ and a radical anion half-lifetime of ∼0.5 s in THF, enabling bimolecular follow-up reactivity. Indeed, two-electron reduction and protonation of **3** with KC_8_/[DBUH]^+^ (DBU = 1,8-diazabicyclo[5.4.0]­undec-7-ene) or hydride transfer from Li­(AlH_4_) affords the borohydride **4** ^**–**^. From a hydricity–potential correlation, we estimate a hydricity of ∼36 kcal mol^–1^ in MeCN, which allows **4** ^**–**^ to reduce H^+^ to H_2_ and CO_2_ to formate under ambient conditions in almost quantitative yields. The reaction of **3**, [DBUH]^+^, KC_8_, and CO_2_ furnishes potassium formate in ∼90% spectroscopic yield, establishing a closed synthetic cycle for CO_2_ reduction by protons and electrons at a boron center. These results highlight how tailor-made, redox-active boranes can complement transition-metal hydrides as modular, metal-free platforms for coupling renewable electrons and protons in CO_2_ utilization chemistry.

## Graphical abstract

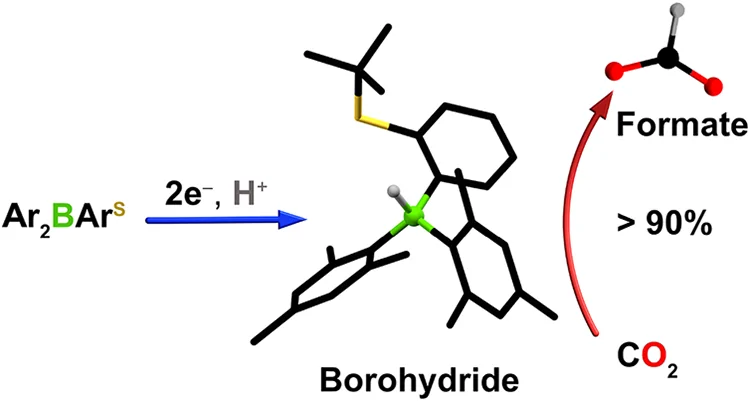

## Introduction

Since their discovery in the early 20th century, borohydrides have become cornerstone hydride donor reagents in organic reductions.^1^ More recently, borohydrides have also drawn interest in sustainable energy applications and in fuel productions.^2^ The first reports of borohydrides reducing CO_2_ to formate date back decades, via direct hydride transfer in either aprotic or protic media.^3^ Since then, stochiometric as well as catalytic protocols based on Na­(BH_4_), pinacolborane (HBpin), or catecholborane (HBcat) as hydride sources have been developed to effectively reduce CO_2_ to formate, formaldehyde, formic acid, or methanol.^4^ However, the inherent disadvantage of such protocols is the energy-expensive formation of the requisite borohydride reagents. To overcome this issue, boron-containing frustrated Lewis acid/base pairs (FLPs) that activate H_2_ heterolytically to generate H^+^/H^–^ equivalents have been used in alternative approaches for the stochiometric or catalytic reduction of CO_2_.^5^ Despite the progress that has been made over the last two decades in this field, the inherent problem of such protocols lies in the high oxophilicity of the Lewis-acidic boron atom, which hampers product release after hydride transfer to CO_2_, and the high H_2_/CO_2_ pressures and elevated temperatures associated with several of these reduction protocols, leading to safety issues.

A notable advantage of borohydrides as hydride sources lies in the facile tunability of their hydride donor strengths.^6^ The thermodynamic driving force to donate a hydride is described by the hydricity term, Δ*G*_H^–^_^0^ (Figure 1A). Depending on the type and number of substituents, borohydrides (R_3_BH^–^) can span a wide range of hydricities ranging from 68 kcal mol^–1^ down to 0.6 kcal mol^–1^, that is from moderate to very strong hydride donor abilities.^7^

**Figure 1:**
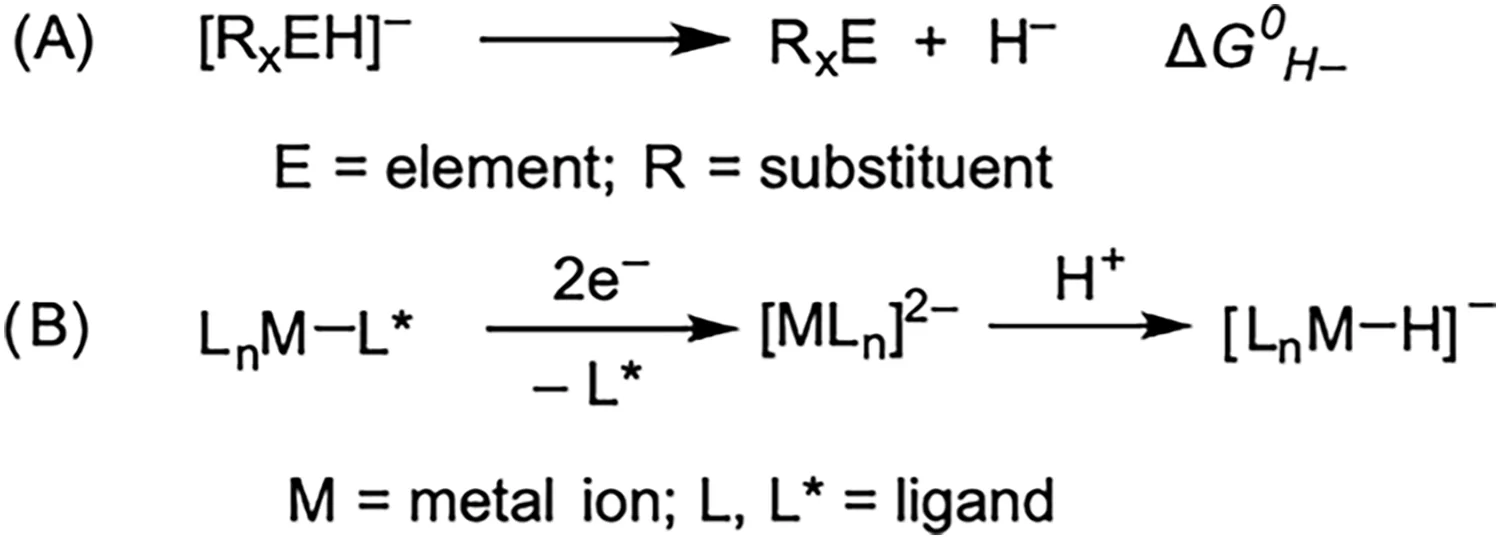
(A) Definition of the thermodynamic hydricity. (B) Common approach to access transition metal hydrides.

Against this background, it is therefore desirable to develop alternative strategies for the synthesis of borohydrides. Looking at transition metal hydride complexes, these are commonly formed by dihydrogen activation or by 2e^–^ reduction of the metal precursor and subsequent protonation (Figure 1B).^8^ The latter strategy seems appealing also for borohydrides, however, in the specific case of R_3_BH^–^, the reduction of the borane precursor R_3_B, if possible at all, requires usually very harsh reductants.^9^ Thus, only very few examples are reported following the approach of 2e^–^ reduction and protonation of R_3_B to access borohydrides and all of them use elaborate strategies to stabilize the intermediate anions, e.g., π-electron delocalization and/or metal ion coordination (**I**-**III**, Figure 2A).^10^

**Figure 2:**
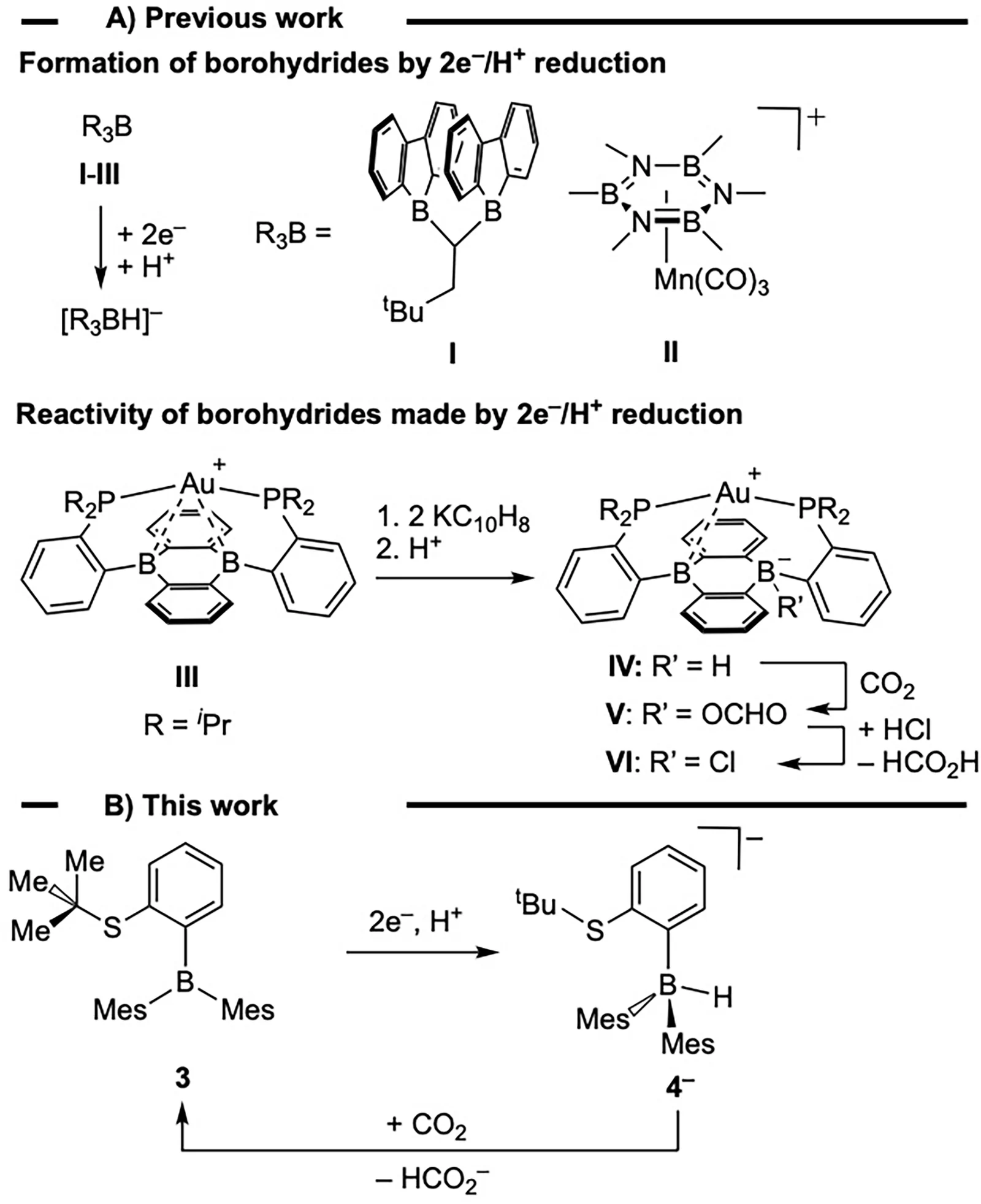
(A) BR_3_ compounds that form borohydrides R_3_BH^–^ upon 2e^–^ reduction and protonation; formation of reduced and protonated borohydride of **II** proposed to exist in situ. (B) Formation of **4** ^**–**^ via reduction and protonation of **3** presented in this work; Mes = 2,4,6-Me_3_C_6_H_2_.

Only for one of the above-mentioned examples, the metal masked borohydride **III**, follow-up hydride transfer reactivity was reported (Figure 2A).^10d^ **III** was reduced by potassium naphthalenide, protonated by [DBUH]^+^ and the unsymmetric borohydride **IV** reacts with CO_2_ leading to the formate adduct **V** (DBU = 1,8-diazabicyclo[5.4.0]­undec-7-ene). Formate can be released from **V** by adding HCl as a strong acid.

Inspired by the above-mentioned results on borohydride formation and reactivity, we aimed at developing a synthetic cycle for CO_2_-to-formate reduction mediated by a borohydride that is formed by reduction and protonation, and specifically to design the borane such that formate is released in the end under ambient conditions without the need of a harsh coreagent. Steric effects and spin delocalization through π-conjugation are important factors that make the intermediate anionic boron-centered radical, R_3_B^•–^, accessible and control its stability.^9b–9g^ Thus, we hypothesized that the thioether-substituted triarylborane 1,2-(^*t*^BuS)­C_6_H_4_(BMes_2_) (**3**) (Mes = 2,4,6-Me_3_C_6_H_2_) would be a promising candidate to promote the electrochemical reduction of CO_2_. In addition to providing sufficient hydricity within its hydridoborate **4** ^–^ (i.e., [**3**-H]^−^) congener for CO_2_ activation, the steric congestion provided by the bulky *tert*-butyl and mesityl groups should encourage the release of formate (Figure 2B).

## Results and Discussion

1,2-(^*t*^BuS)­C_6_H_4_(BMes_2_) (**3**) is synthesized in a three-step procedure starting from 2-bromobenzenethiol, as depicted in Figure 3.

**Figure 3:**
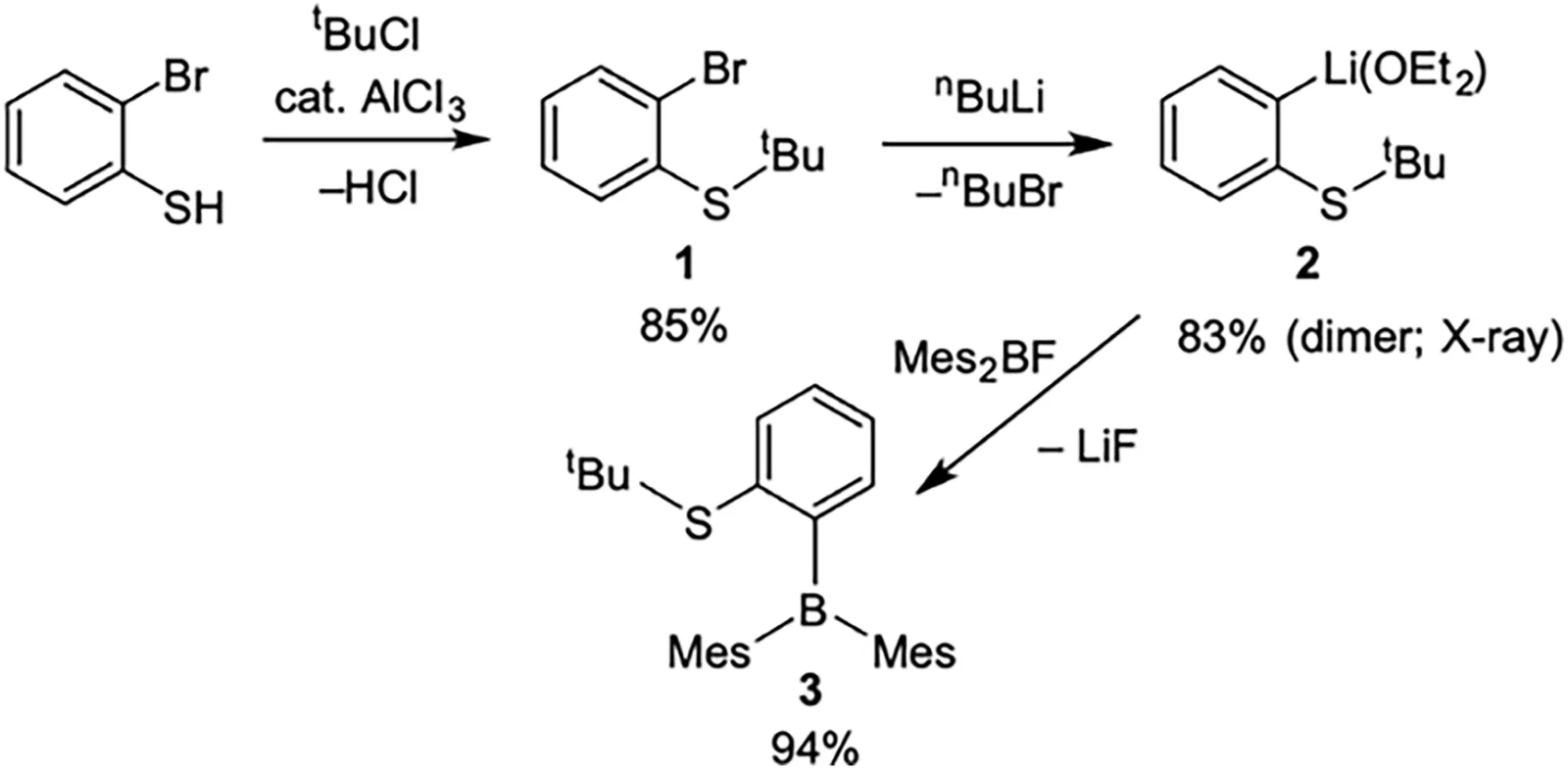
Synthesis of the borane **3**.

2-(*Tert-*butylthio)-bromobenzene, **1**, is synthesized in an AlCl_3_-catalyzed thiolation according to a modified literature procedure in a yield of 85%.^11^ Subsequent halogen lithium exchange with *n*-butyllithium affords (2-(*tert*-butylthio)­phenyl)­lithium, **2**, which is isolated as colorless crystals **2**·OEt_2_ in an 83% yield. The formation of **2**·OEt_2_ is supported by ^1^H, ^13^C­{^1^H}, and ^7^Li­{^1^H} NMR spectroscopy (Figures S10–S12) and its purity established by elemental analysis. Single crystals of **2**·OEt_2_ that were of suitable quality for X-ray crystallography were obtained by cooling a concentrated diethyl ether solution of **2** to –35 °C (Figure 4 (a), SI). Compound **2**·OEt_2_ adopts a centrosymmetric dimeric structure with each Li^+^ ion linking two aryl units with an added OEt_2_ molecule and weak Li---S contacts completing the coordination sphere.

**Figure 4:**
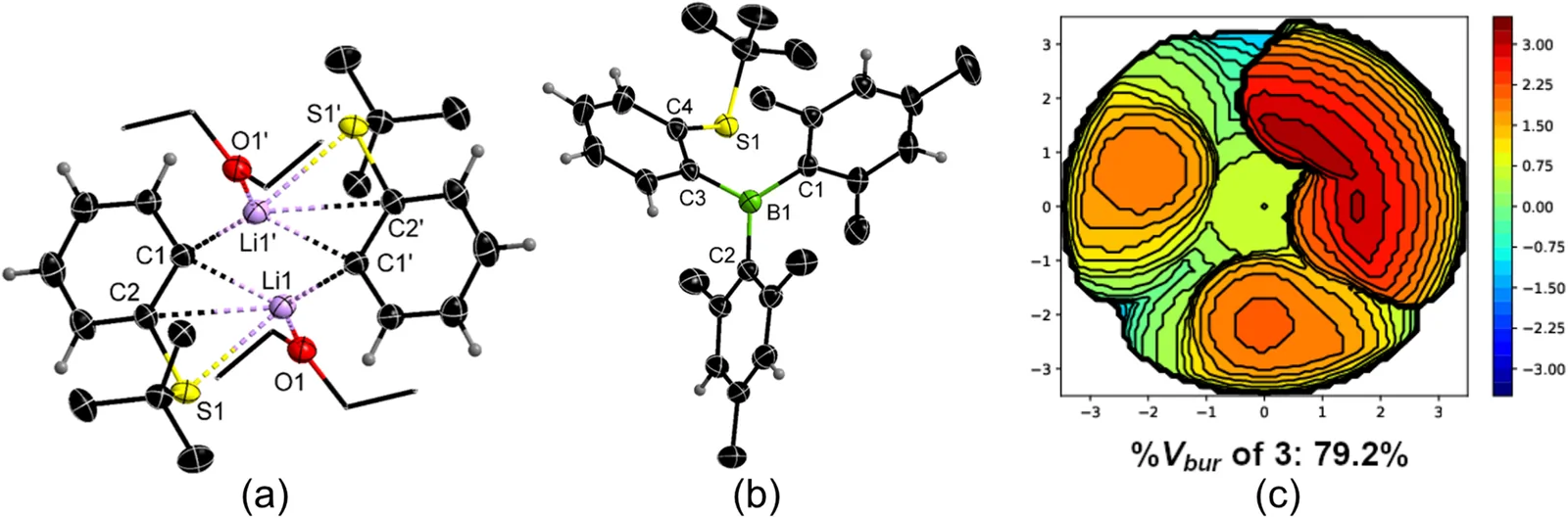
(a) Crystal structure of **2**·OEt_2_ (selected structural parameters (pm): C1–Li1 = 218.3(8), C1–Li1′ = 221.6(8), C2–Li1 = 270.5(10), S1–Li1 = 263.4(11); O1–Li1 = 193.5(12)). (b) **3** (selected bond lengths (pm) and angles (°): S1···B1 = 312.6(3), B1–C1 = 157.4(3), B1–C2 = 158.3(3), B1–C3 = 157.9(2), C3–C4 = 140.2(3)); ellipsoids are drawn at 35% probability level except for the H atoms that are depicted with arbitrary radii; H atoms of the CH_2_ and CH_3_ moieties are omitted for clarity and the C atoms of Et_2_O are depicted with a wire frame model. (c) Steric map of **3** derived from its fluoride adduct.

Combining **2** with dimesitylfluoroborane (Mes_2_BF) in Et_2_O affords 1,2-(^*t*^BuS)­C_6_H_4_(BMes_2_) (**3**) in an excellent yield of 94%. **3** can also be prepared in a one-pot procedure starting from **1** in a yield of 92%. Compound **3** affords ^1^H and ^13^C­{^1^H} NMR spectra consistent with the assigned structure, while a singlet is noted at 75 ppm in its ^11^B NMR spectrum in C_6_D_6_, illustrating a lack of strong intramolecular B---S bonding and retention of a three-coordinate B center in solution (Figures S13–S15). Colorless block-shaped X-ray quality crystals are obtained by cooling a concentrated solution of **3** in Et_2_O to −35 °C (Figure 4(b), SI). The boron atom in **3** exhibits a trigonal-planar geometry (∑*B* = 359.6(3)°) with B–C bond lengths in the narrow range of 157.4(3) pm to 158.3(3) pm. The distance between the B and the S atom is 312.6(3) pm, which is shorter than the sum of van der Waals radii of the respective elements (*r* ^vdW,B^ = 208 pm, *r* ^vdW,S^ = 206 pm).^12^ This might indicate weak interactions and in turn, DFT computations were performed using Gaussian16 ^13^ with the M06–2X^14^ functional, Grimme’s D3 dispersion correction,^15^ and the cc-pVTZ^16^ basis set (see SI). However, subsequent NBO analysis could not locate a discrete B---S interaction, while the associated Wiberg Bond Index between the B and S atoms is only 0.071.^17^ This confirms the experimental and spectroscopic results that there is no significant B---S interaction and in turn, the effect of the *tert*-butyl group is purely steric in nature.

Compound **3** is remarkably stable under air as a solid, without any decomposition over 4 months. This suggests that the Lewis-acidic boron center is indeed significantly sterically protected by the *tert*-butylthioether substituent, which is one key determinant of redox and moisture stability of triarylboranes.^18^ Indeed, the calculated percent buried volume^19^ (%*V*_bur_) of **3** as obtained by analyzing the corresponding fluoride adduct [**3–**F]^**–**^ is rather large (79.2%) with some added shielding of the B center due to the proximal *tert*-butyl group (Figure 4(c)); for comparison, the estimated %*V*_bur_ values for BMes_3_ and B­(C_6_Cl_5_)_3_ are 71.0% and 70.2%, respectively.^20^

Since a goal of this project is the synthesis of the borohydride **4** ^**–**^ from **3** by addition of electrons and protons, we then investigated the electrochemical properties of **3** by cyclic voltammetry (CV). The resulting CV data revealed an irreversible reduction process at a peak potential *E*_p_ = −2.66 V vs Fc^+|0^ and two anodic features at *E*_p,a_ = −1.38 V vs Fc^+|0^ and *E*_p,a_ = −0.57 V vs Fc^+|0^ in THF with ^*n*^Bu_4_NBPh_4_ as the electrolyte (scan rate ν = 0.1 V s^–1^; Figure 5(a)). The peak potential of the reductive event remains virtually unchanged when moving to ^*n*^Hex_4_NBAr_4_^F^ (Ar^F^ = 3,5-(F_3_C)_2_C_6_H_3_) as the supporting electrolyte in either THF or 1,2 difluorobenzene (DFB) as solvent (Figure S4). However, in both CV data sets two additional oxidative events appear, indicating that the fluorinated solvent/electrolytes might impact the reaction pathway of the reduced radical species of **3** (Figures S4–S7). Thus, we analyzed further the CV data of **3** collected in THF with ^*n*^Bu_4_NBPh_4_ as the electrolyte.

**Figure 5:**
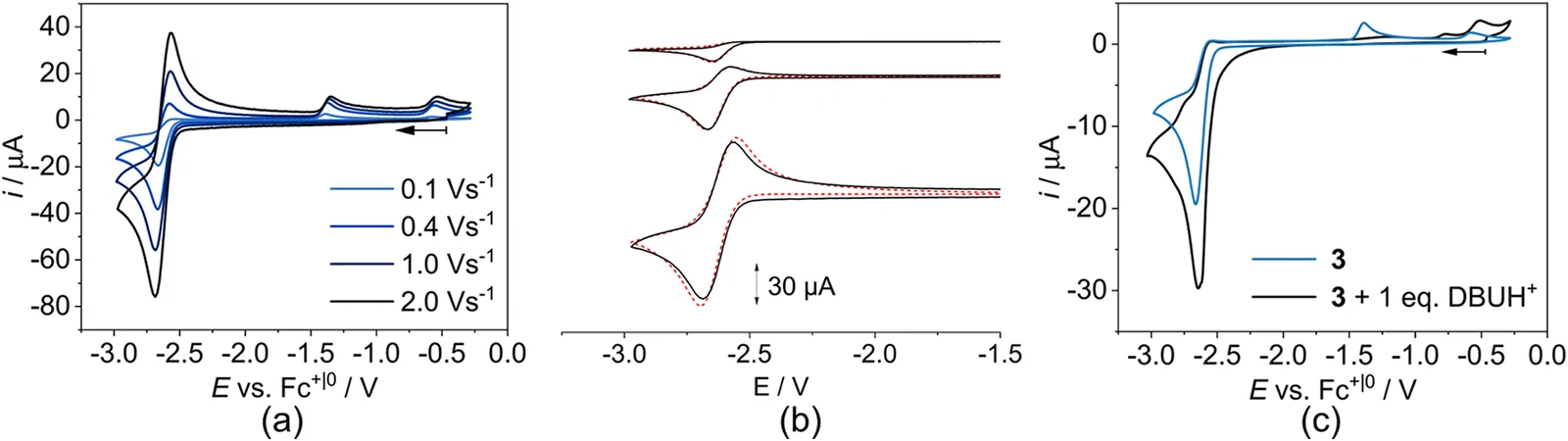
(a) CV data of **3** in THF, *I* = 0.1 M ^n^Bu_4_NBPh_4_, c_**3**_ ≈ 1 mM; *v* = 0.1, 0.4, 1.0, 2.0 V s^–1^. (b) Selected simulated (red dotted line) and measured (black line) CV data of **3** in THF, *I* = 0.1 M ^n^Bu_4_NBPh_4_; *c*_**3**_ ≈ 1 mM; *v* = 0.1, 0.4, 2 V s^–1^, simulation parameters see text and SI. (c) Overlay of the CV data of **3** and **3** + 1 eq. [DBUH]­[BPh_4_] in THF, *I* = 0.1 M *^n^* Bu_4_NBPh_4_, *c*_**3**_ ≈ 1 mM; *v* = 0.1 V s^–1^.

The missing reverse feature of the reduction event and multiple oxidation peaks indicates a follow-up reaction of **3** after reduction. Indeed, upon increasing the scan rate to 2 V s^–1^ the oxidative reverse feature at *E*_pa_ = −2.57 V vs Fc^+|0^ appears in the CV data indicating a chemical reaction upon reduction on the time scale of the CV (Figure 5(a)). Simulation of the scan rate dependent measurements allows for the determination of *E* ^0^ of **3** and the (pseudo) first-order rate constant *k* for the follow-up chemical reaction. Decent fits were obtained by applying a Butler–Volmer model over a large scan rate range (0.1–2 V s^–1^). Representative experimental data and fits are presented in Figure 5(b); further data showing the other scan rates and the simulation details are in the SI. The redox potential *E* ^0^ of **3** equals −2.62 V vs Fc^+|0^ in THF, which is very similar to the redox potential of BMes_3_, which equals −2.57 V vs Fc^+|0^ in MeCN.^21^ This further supports that there are no substantial interactions between the adjacent thioether group and the boron atom and in turn, the redox potential is governed by the steric effect of the substituents and spin delocalization through π-conjugation.^9b–9e^ The (pseudo) first order rate constant *k* for the follow up chemical reaction of **3** ^**•–**^ equals 1.4 s^–1^, which is similar to the decay constant of B­(C_6_F_5_)_3_^•–^ in DFB, but much slower than the decay constant of B­(Mes)_2_(C_6_F_5_)^•–^ in THF.^9e,22^

The half-life time τ^1/2^ of 0.5 s indicates reasonable stability of the radical anion of **3** in THF, which should allow for productive bimolecular follow-up reactivity, e.g., proton capture. Thus, we evaluated the redox chemistry of **3** in the presence of [DBUH]­[BPh_4_] as a proton source. Upon addition of one equivalent of the acid the peak potential of the initial reduction of **3** is virtually unaffected (*E*_p_ = −2.65 V vs Fc^+|0^ in THF; *ν* = 0.1 V s^–1^), however, the current increases indicating multiple electron transfer processes (Figures 5(c), and S9). Furthermore, the current of the reverse oxidation features at higher potentials, which were present in the CV data of **3**, almost all vanish, indicating a different follow-up reactivity than without acid. Intrigued by this observation, we aimed to synthesize the borate **4** ^**–**^ via two different routes: (1) by addition of electrons and a proton and (2) directly by hydride ion transfer (Figure 6).

**Figure 6:**
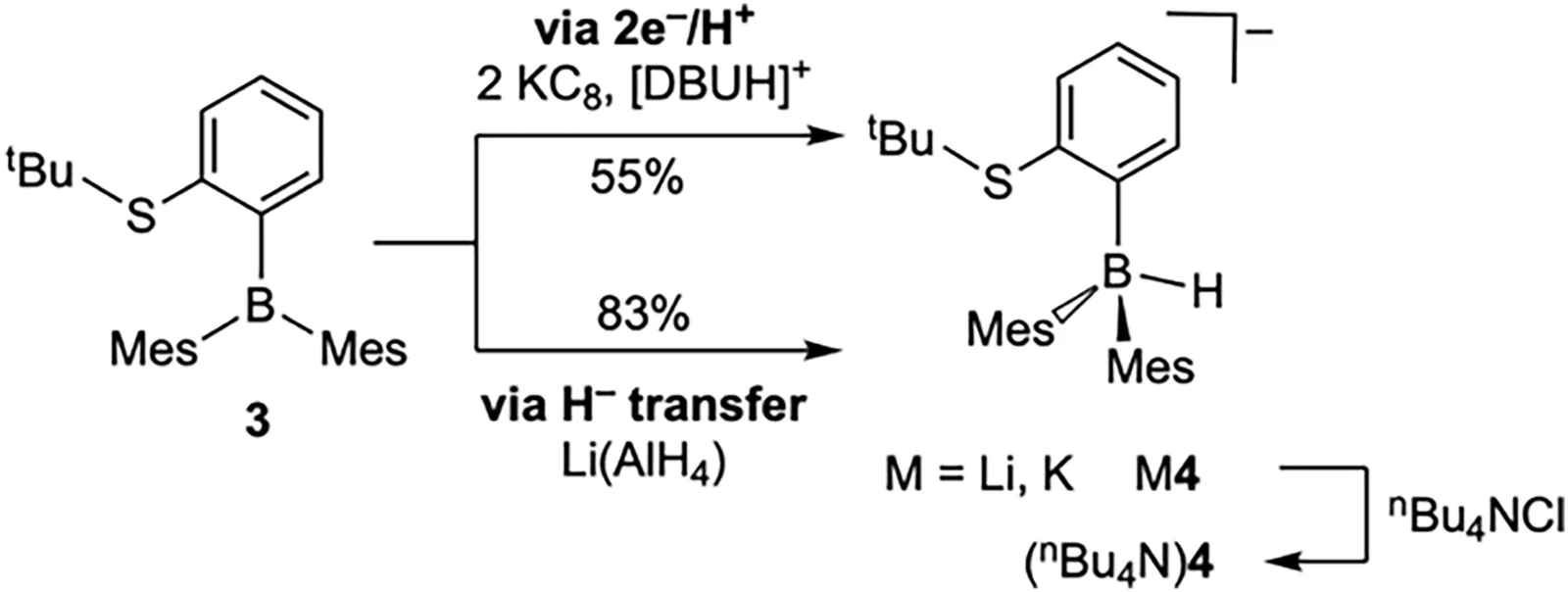
Synthesis of **4** ^**–**^.

Indeed, the reduction of **3** with KC_8_ in the presence of [DBUH]^+^ in THF yielded the potassium salt of **4** ^**–**^ in a spectroscopic yield of 55% within 10 min as determined by ^11^B NMR spectroscopy with ^*n*^Hex_4_NBAr_4_^F^ as an internal standard (Figure S29). Treating **3** with 1.1 eq. Li­(AlH_4_) in THF leads to the formation of the borohydride salt [Li­(THF)_3_]**4** as a white solid in an isolated yield of 83%. Counter ion exchange in Li**4** by salt metathesis using ^*n*^Bu_4_NCl is facile leading to (^*n*^Bu_4_N)**4**, which was isolated in a quantitative yield (see SI for details). The resulting Li^+^ and ^*n*^Bu_4_N^+^ salts of **4** ^**–**^ were comprehensively characterized and their bulk purities were confirmed by elemental analyses (Figures S16–S28). The ^11^B NMR spectrum of Li**4** in CD_3_CN shows a doublet at −14.1 ppm with ^1^ *J*_BH_ = 80.4 Hz, which is characteristic for triarylborohydrides (Figures S16–S28).^23^ Crystals of [Li­(THF)_4_]**4** and (^*n*^Bu_4_N)**4** suitable for single X-ray diffraction experiments were obtained and the results of the refinement can be found in Figure 7 and the SI. As expected, the boron atom in [Li­(THF)_4_]**4** exhibits a distorted tetrahedral geometry. The spectroscopic parameters of the borate anions **4** ^**–**^ in solution and the solid state are very similar, irrespective of the countercation; that is, the exchange of the counterion has no impact on the (electronic) structure of the anion **4** ^**–**^.

**Figure 7:**
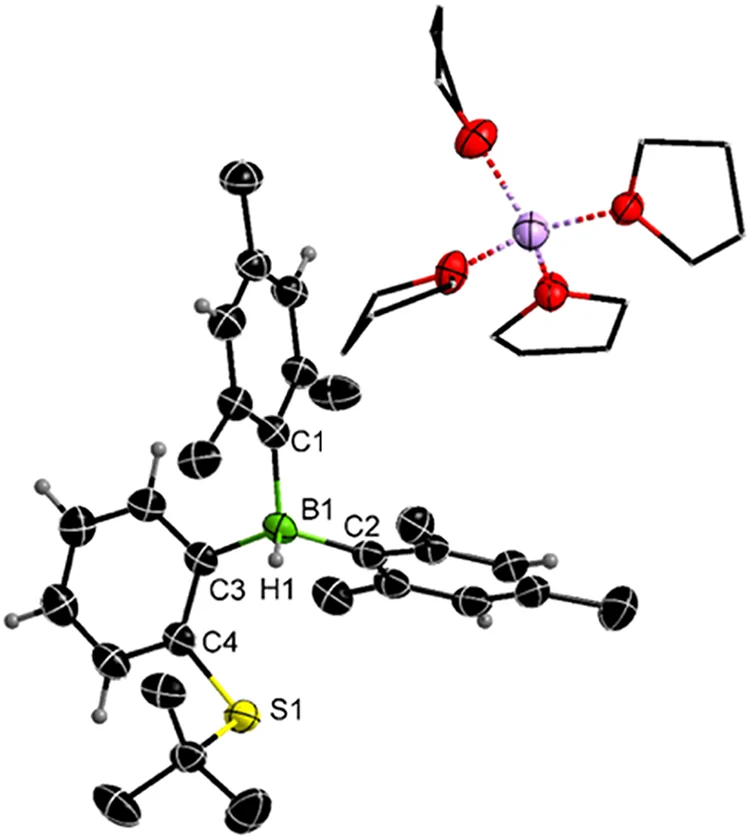
Molecular structure of [Li­(THF)_4_]**4** (thermal ellipsoids depicted at a 35% probability level, except for the H atoms that are depicted with arbitrary radii). H atoms of the CH_2_ and CH_3_ moieties are omitted for clarity and the C atoms of THF are depicted with a wire frame model. selected bond lengths (pm) and angles (°): B1–H = 113.2(26), B1–C1 = 165.6(3), B1–C2 = 165.3(3), B1–C3 = 164.1(3); C1–B1–C2 = 112.5(2), C2–B1–C3 = 120.1(2), C3–B1–C1 = 109.7(2), H1–B1–C1 = 108.0(13), C3–B1–H1 = 104.1(14), H1–B1–C2 = 101.2(13).

We then investigated the hydride transfer reactivity involving **4** ^**–**^. A very comprehensive study by Glusac and co-workers analyzing 280 hydride donors showed that there is a correlation between the initial redox potential and the thermodynamic hydricity of a given nonmetal hydride species.^7^ The hydricity Δ*G*_H^–^_^0^ of nonmetal hydride donors decreases linearly with decreasing initial reduction potential *E*_1_^0^. In other words, the more negative the potential of the initial reduction is, the easier the thermodynamic hydride transfer becomes. Taking Glusac’s correlation,^7^ we estimated a hydricity Δ*G*_H^–^_^0^ of approximately 36 kcal mol^–1^ for **4** ^**–**^ in MeCN (see SI for detailed derivation). This means that **4** ^**–**^ is a potent hydride transfer reagent sufficient to react with H^+^ or CO_2_, because the hydricity of HCO_2_^–^ equals 44 kcal mol^–1^ and the hydricity of H_2_ is 76 kcal mol^–1^, both in MeCN.^24^

Indeed, adding [DBUH]^+^ (p*K*_a_ = 24.1 in MeCN)^25^ to a solution of Na**4** or (^*n*^Bu_4_N)**4** in THF leads to the formation of H_2_ and **3** (Figures S42 and S43, see the SI for thermodynamic analysis). This behavior may also explain why the yield in the above-mentioned experiment forming **4** ^**–**^ from electrons (KC_8_) and protons ([DBUH] ^+^) is lower than via the hydride route, because in situ formed **4** ^**–**^ and unreacted [DBUH]^+^ could reform **3**, which lowers the yield of **4** ^**–**^. Subsequently, we exposed a solution of Li**4** in THF to 1 atm CO_2_ at room temperature, which resulted in the immediate precipitation of a colorless microcrystalline solid (Figure 8). NMR spectroscopic analysis of the supernatant confirmed the quantitative regeneration of **3** (isolated yield 90%) and the isolated precipitate was identified as lithium formate via ^1^H and ^13^C­{^1^H} NMR spectroscopy in D_2_O (Figures S30–S33). Noteworthy, the reaction was conducted under ambient conditions with nondried solvents. The high thermodynamic hydricity of Li**4** is further evidenced by its ability to capture atmospheric CO_2_ under ambient air conditions as solid, affording lithium formate and **3** with a half-life of approximately 4 weeks. In order to exclude precipitation of lithium formate as main driving force for the CO_2_ reduction, (^*n*^Bu_4_N)**4** was also exposed to CO_2_ and the homogeneous reaction mixture selectively yielded (^*n*^Bu_4_N)­(HCO_2_) and **3** within 10 min, as confirmed by ^1^H, ^13^C­{^1^H}, and ^11^B NMR spectroscopy (Figures S34–S36). Intrigued by these results, we evaluated the stochiometric CO_2_-to-formate conversion starting with **3**, protons, and electrons. Indeed, mixing **3** with 1 equiv of [DBUH]^+^ and 2 equiv of KC_8_ in THF under a ^13^CO_2_ atmosphere of 1.2 bar leads to the rapid formation of potassium formate as precipitate. Potassium formate was formed in spectroscopic yields of 90% as confirmed by ^1^H and ^13^C­{^1^H} spectroscopy in D_2_O using MeCN as an internal standard (Figures S37–S40). The high spectroscopic yield of [HCO_2_]^−^ shows that the reaction of **4** ^**–**^ with CO_2_ as an electrophile is faster than the reaction of **4** ^**–**^ with H^+^, despite the fact that the latter reaction has the larger driving force.

**Figure 8:**
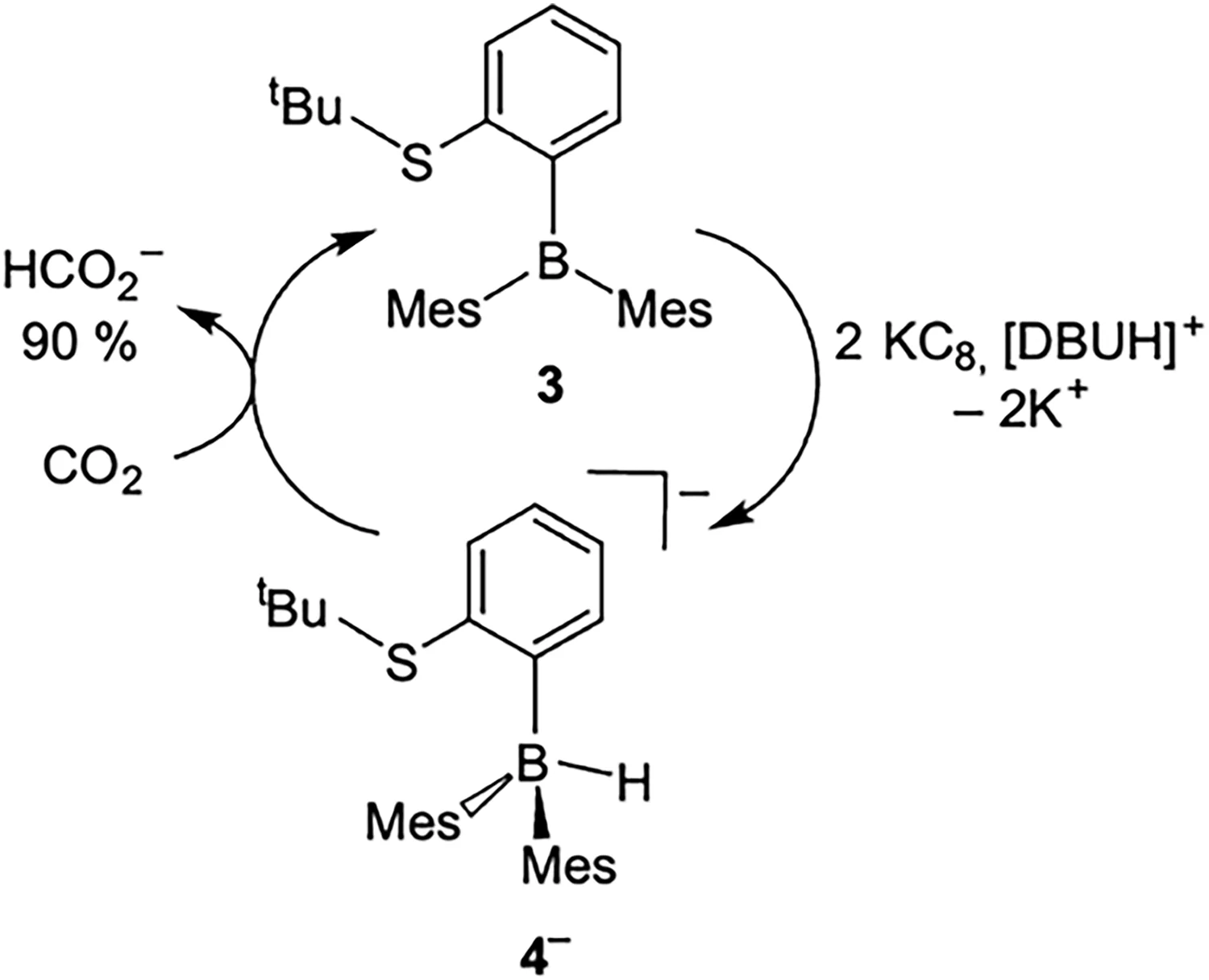
Closed synthetic cycle for the reduction of CO_2_ to formic acid with protons and electrons mediated by **3**.

Catalytic turn over using 1 equiv of **3**, 20 equiv of KC_8_, and 10 equiv of [DBUH]^+^ in THF under a ^13^CO_2_ atmosphere of 1.2 bar was not successful (TON of 1.3), likely due to the low solubility of [DBUH]^+^ in THF. Using 1600 equiv of KH instead of KC_8_/[DBUH]^+^ as hydride transfer reagent, we observed catalytic formate formation with a TON of 5.3 × 10^2^ within 1 h, highlighting the application of **3** in catalytic hydrogenation reactions.

## Conclusions

We have demonstrated that the sterically encumbered triarylborane 1,2-(^*t*^BuS)­C_6_H_4_(BMes_2_) (**3**) can be transformed by two electrons and a proton into the borohydride **4** ^**–**^, whose strong, yet tunable, hydride donor ability enables selective CO_2_-to-formate conversion under ambient conditions. The combination of electrochemical, spectroscopic, and crystallographic data establishes **3**/**4** ^–^ as a rare example of a redox-addressable borane/borohydride pair with a well-defined thermodynamic hydricity that competes with established main-group and transition-metal hydride platforms for CO_2_ reduction. By closing a synthetic cycle in which **3**, KC_8_, [DBUH]^+^, and CO_2_ are converted directly into the formate salt in 90% spectroscopic yield and regeneration of **3**, this work showcases how sterically protected, redox-active boranes can function as transition metal-free reaction centers that couple electrons and protons for small-molecule valorization, and it provides design principles for future boron-based systems for CO_2_ utilization and related transformations. Future studies will explore the modification of the mesityl substituents at the boron atom to tune the thermodynamic hydricity of this borohydride platform.^6a,7^ Expanding the substrate scope to other synthetically useful electrophiles will further establish the versatility of these redox-addressable borane/borohydride pairs beyond small-molecule transformations.

## Experimental Details

If not otherwise stated, manipulations of air-sensitive reagents were carried out by means of common Schlenk-type techniques involving the use of a dry argon or nitrogen atmosphere or performed in a glovebox manufactured by either MBraun or Innovative Technologies, Inc..

### Synthesis and Characterization of 2-(*tert-*Butylthiophenyl)­lithium, **2**·OEt_2_

A solution of *n*-butyllithium in hexanes (2.5 M, 2.00 mL, 5.0 mmol) was added dropwise to a solution of **1** (1.23 g, 5.00 mmol) in Et_2_O (6 mL) at room temperature. After stirring at room temperature for 5 min, the solution was cooled to −35 °C for 16 h. This afforded **2**·OEt_2_ as colorless crystals, which were separated from the mother liquor by decantation and dried under vacuum (885 mg, 3.59 mmol). The mother liquor was concentrated to a volume of ca. 4 mL and stored at −35 °C for 40 h to yield another crop of **2**·OEt_2_ as colorless crystals, which were separated from the mother liquor by decantation and dried under vacuum (141 mg, 0.572 mmol). Combined yield: 1.03 g (4.16 mmol, 83%).

^1^H NMR (C_6_D_6_, 399.78 MHz): δ 8.42 (dd, ^3^ *J*(^1^H,^1^H) = 6.6 Hz, ^4^ *J*(^1^H,^1^H) = 1.1 Hz, 1H, ^Ar^C*H*), 7.57 (d, ^3^ *J*(^1^H,^1^H) = 7.6 Hz, 1H, ^Ar^C*H*), 7.28 (tm, 1H, ^3^ *J*(^1^H,^1^H) = 7.0 Hz, ^Ar^C*H*), 7.13 (td, ^3^ *J*(^1^H,^1^H) = 7.5 Hz, ^4^ *J*(^1^H,^1^H) = 1.6 Hz, 1H, ^Ar^C*H*), 3.21 (q, ^3^ *J*(^1^H,^1^H) = 7.0 Hz, 4H, ^Et2O^C*H*_2_), 1.05 (s br, 9H, ^*t*Bu^C*H*_3_), 3.21 ppm (t, ^3^ *J*(^1^H,^1^H) = 7.0 Hz, 6H, ^Et2O^C*H*_3_).

^13^C­{^1^H} NMR (C_6_D_6_, 100.52 MHz): δ 147.7 (s, ^Ar^ *CS*), 140.7 (s, ^Ar^ *C*H), 134.0 (s,^Ar^ *C*H), 127.4 (s, ^Ar^ *C*H), 127.1 (s, ^Ar^ *C*H), 64.8 (s, ^Et2O^ *C*H_2_), 47.0 (s, ^*t*Bu^ *C*), 31.7 (s, ^Et2O^ *C*H_3_), 15.3 ppm (s, ^*t*Bu^ *C*H_3_). *Note*: *the resonance for the ArC bonded to lithium could not be located*.

^7^Li­{^1^H} NMR (C_6_D_6_, 155.36 MHz): δ –3.2 ppm (s).

#### Elemental Analysis

Calculated (%) for C_14_H_23_LiOS, C 68.26, H 9.41, S 13.01; found, C 69.06, H 8.85, S 12.92.

### Synthesis of **3**

Dimesitylfluoroborane (829 mg, 3.09 mmol) was slowly added to a solution of **2**·OEt_2_ (762 mg, 3.09 mmol) in Et_2_O (8 mL) and stirred at room temperature for 16 h. The reaction mixture was filtered, and the solution was concentrated to a volume of ca. 3 mL. Cooling of the solution to −35 °C for 16 h afforded **3** as pale-yellow crystals, which were separated from the mother liquor by decantation and dried under vacuum (1.02 g, 2.46 mmol). The mother liquor was concentrated to ca. 2 mL and stored at −35 °C for 40 h to yield another crop of **3** as pale-yellow crystals, which were separated from the mother liquor by decantation and dried under vacuum (185 mg, 0.446 mmol). Combined yield of **3**: 1.13 g (2.91 mmol, 94%).

^1^H NMR (CDCl_3_, 498.10 MHz): δ 7.54 (d, ^3^ *J*(^1^H,^1^H) = 7.9 Hz, 1H, ^Ar^C*H*), 7.33–7.27 (m, 1H, ^Ar^C*H*), 7.20–7.15 (ov m, 2H, ^Ar^C*H*), 6.72 (s, 4H, ^Mes^C*H*), 2.26 (s, 6H, ^*p*‑CH3^C*H*), 2.01 (br s, 12H, ^*o*‑CH3^C*H*), 2.26 ppm (s, 9H, ^*t*Bu^C*H_3_*).

^13^C­{^1^H} NMR (CDCl_3_, 125.25 MHz): δ 154.6 (s, ^Ar^ *CS*), 143.4 (s, ^Ar^ *C*B), 140.9 (s,^Mes^ *C*B), 138.9 (s, ^Mes^ *C*), 138.8 (s, ^Mes^C), 133.9 (s, ^Ar^ *C*H), 133.4 (s, ^Ar^ *C*H), 129.3 (s, ^Ar^ *C*H), 128.3 (s, ^Mes^ *C*H), 126.5 (s, ^Ar^ *C*H), 47.6 (s, ^*t*Bu^ *C*(CH_3_)_3_), 31.9 (s, ^*t*Bu^ *C*H_3_), 23.3 (br s, *o*-*C*H_3_), 21.4 ppm (s, *p*-*C*H_3_).

^11^B­{^1^H} NMR (CDCl_3_, 128.3 MHz): δ 75.0 ppm (s).

#### Elemental Analysis

Calculated (%) for C_28_H_35_BS, C 81.14, H 8.51, S 7.74; found, C 82.11, H 8.65, S 7.68.

### Synthesis of [Li­(THF)_3_]­4 via Hydride Transfer

**3** (95.3 mg, 0.230 mmol) was added slowly to a solution of Li­(AlH_4_) (10.0 mg, 0.264 mmol) in THF (3 mL) and stirred at room temperature for 5 min. The volatiles were removed under vacuum and the remaining colorless solid was suspended in toluene (3 mL). THF (3 mL) was added dropwise under string to give an almost clear mixture, which was filtered. Cooling of the filtrate to −35 °C for 16 h afforded [Li­(THF)_4_]**4** as colorless crystals, which were separated from the mother liquor by decantation and dried under vacuum for 2 h yielding a THF solvate content of [Li­(THF)_3_]**4**. Yield: 121 mg (0.190 mmol, 83%).

#### Data for [Li­(THF)_3_]**4**

^1^H NMR (CD_3_CN, 399.98 MHz): δ 7.30 (d, ^3^ *J*(^1^H,^1^H) = 7.5 Hz, 1H, ^Ar^C*H*), 6.92–6.85 (m, 1H, ^Ar^C*H*), 6.79 (t, ^3^ *J*(^1^H,^1^H) = 7.3 Hz, 1H, ^Ar^C*H*), 6.72 (t, ^3^ *J*(^1^H,^1^H) = 7.2 Hz, 1H, ^Ph^C*H*), 6.45 (s, 4H, ^Mes^C*H*), 3.83 (q, ^1^ *J*(^11^B,^1^H) = 80.4 Hz, 1H, B*H*), 3.69–3.62 (m, 12H, THF–C*H*_2_), 2.13 (s, 6H, ^*p*‑CH3^C*H*), 1.85 (br s, 12H, ^*o*‑CH3^C*H*), 1.84–1.77 (m, 12H, THF–C*H*_2_), 1.34 ppm (s, 9H, ^*t*Bu^C*H*_3_).

^1^H­{^11^B} NMR (CD_3_CN, 498.10 MHz): δ 7.30 (d, ^3^ *J*(^1^H,^1^H) = 7.5 Hz, 1H, ^Ar^C*H*), 6.92–6.85 (d, ^3^ *J*(^1^H,^1^H) = 7.3 Hz, 1H, ^Ar^C*H*), 6.79 (t, ^3^ *J*(^1^H,^1^H) = 7.3 Hz, 1H, ^Ar^C*H*), 6.72 (t, ^3^ *J*(^1^H,^1^H) = 7.2 Hz, 1H, ^Ph^C*H*), 6.45 (s, 4H, ^Mes^C*H*), 3.83 (s, 1H, B*H*), 3.69–3.62 (m, 12H, THF–C*H*_2_), 2.13 (s, 6H, ^*p*‑CH3^C*H*), 1.85 (br s, 12H, ^*o*‑CH3^C*H*), 1.84–1.77 (m, 12H, THF–C*H*_2_), 1.34 ppm (s, 9H, ^*t*Bu^C*H_3_*).

^13^C­{^1^H} NMR (CD_3_CN, 125.25 MHz): δ 171.1 (q, ^1^ *J*(^13^C,^11^B) = 49.5 Hz, ^Ar^ *C*B), 159.3 (q, ^1^ *J*(^13^C,^11^B) = 51.5 Hz, ^Mes^ *C*B), 143.3 (s, ^Mes^ *C*), 142.1 (s, ^Ar^ *CS*), 136.4 (s, ^Mes^ *C*), 133.9 (s, ^Ar^ *C*H), 130.6 (s, ^Ar^ *C*H), 120.0 (s, ^Ar^ *C*H), 125.3 (s, ^Mes^ *C*H), 122.5 (s, ^Ar^ *C*H), 68.3 (s, THF-*C*H_2_), 45.2 (s, ^*t*Bu^ *C*), 32.6 (s, ^*t*Bu^ *C*H_3_), 26.2 (s, THF-*C*H_2_), 25.0 (s, *o*-*C*H_3_), 21.0 ppm (s, *p*-*C*H_3_).

^7^Li­{^1^H} NMR (CD_3_CN, 155.45 MHz): δ – 2.6 ppm (s).

^11^B NMR (CD_3_CN, 160.36 MHz): δ –14.1 ppm (d, ^1^ *J*(^11^B,^1^H) = 80.4 Hz).

^11^B­{^1^H} NMR (CD_3_CN, 160.36 MHz): δ –14.1 ppm (s).

IR (THF drop-cast film, KCl plate): 2325 cm^–1^.

#### Elemental Analysis

Calculated (%) for C_40_H_57_BLiO_3_S, C 75.58, H 9.04, S 5.04; found, C 74.65, H 9.36, S 5.08.

### Synthesis of K4 via Reduction and Protonation

5 mg of **3** (12 μmol, 1 equiv) were dissolved in 0.5 mL dry and degassed THF. Afterward 5.7 mg of [DBUH]­[BPh_4_] (12 μmol, 1 equiv) were added. A suspension of KC_8_ (2 eq., 3.3 mg, 24 μmol) in 0.1 mL of THF was added to the solution of **3** and [DBUH]­[BPh_4_], mixed, transferred into a J-Young NMR tube and allowed to react for 10 min. To remove insoluble products, the supernatant was then decanted off into a J-Young NMR tube. The ^1^H and ^11^B NMR spectroscopic data matches those reported for [Li­(THF)_3_]**4** (see above). A spectroscopic yield of K**4** (55%) was determined by ^11^B NMR spectroscopy upon addition of 9.1 mg (7.5 μmol) ^*n*^Hex_4_NBAr_4_^F^ as internal standard (Ar^F^ = 3,5-(F_3_C)_2_C_6_H_3_).

## Supplementary Material





## Notes

Additional experimental procedures, CV data, all NMR data, IR data, UV–vis-data, details on X-ray refinement, computational details (PDF)
Coordinates of **3**, **3**F^–^, **4** ^**–**^ (XYZ)
