# Supplementary material for: A Sterically Encumbered Triarylborane Enabling CO2 Reduction to Formate by Electrons and Protons
Source: Inorg Chem. 2026 Oct 3;65(41):24410–7. doi: 10.1021/acs.inorgchem.6c03816 (PMC13685415; doi:10.1021/acs.inorgchem.6c03816)
Supplement: Supplementary file 1 [file ic6c03816_si_001.pdf]

Supporting Information

A Sterically Encumbered Triarylborane Enabling CO_2 Reduction to Formate by Electrons and Protons

Felix Meyer,[1,+] Ludwig Zapf,[2,+] Eric, Rivard,[2,*] Inke Siewert[1,*]

[+]: equal contribution

1: Universität Göttingen, Institut für Anorganische Chemie, Tammannstr. 4, 37077 Göttingen, Germany

2: University of Alberta, Department of Chemistry, 11227 Saskatchewan Dr. NW, Edmonton, AB, T6G 2G2, Canada

E-Mail: inke.siewert@chemie.uni-goettingen.de, erivard@ualberta.ca

Table of Content

Thermodynamic considerations S2

Estimation of the hydricity of 4^- S2

Thermodynamic analysis of the reaction of 4^- with CO_2: S2

Thermodynamic analysis of the reaction of 4^- with H^+: S2

Details on the simulation of the CV data of 3 ..... S2

Experimental section S3

General details S3

Instrumentation S3

Chemicals S3

X-Ray crystallography S7

CV data S11

NMR data S14

IR data S33

UV/Vis data S34

Computational Details S34

Thermodynamic considerations

Estimation of the hydricity of 4$^-$

All calculations regarding the hydricity of **4**$^-$ refer to MeCN as solvent. A thorough study by Glusac and co-workers analyzing 280 hydride donors showed that there is a linear correlation between the initial redox potential and the thermodynamic hydricity of a given non-metal hydride species, as given in eq. 1.[1]

$$\Delta G^0_{H^-} = [26.3\ \mathrm{kcal\ V^{-1}\ mol^{-1}} \cdot E^0] + 83.76\ \mathrm{kcal\ mol^{-1}} \quad (1)$$

$\Delta G^0_{H^-}$ denotes the hydricity in MeCN, E^0 the initial redox potential of the non-protonated hydride donor *vs*. NHE (Scheme S 1).

$$R_xE \xrightarrow{E^0_1} R_xE^- \xrightarrow{E^0_2} R_xE^{2-} \xrightarrow{+\ H^+} R_xEH^-$$

R = subsituent
E = any element

Scheme S 1. Relationship between the initial redox potential and the thermodynamic hydricity.

We determined a redox potential of –2.62 V *vs*. $Fc^{+|0}$ for **3** in THF, which is rather independent of the solvent (Figure S 4). –2.62 V *vs*. $Fc^{+|0}$ for **3** converts to a redox potential of −1.82 V vs. NHE taking the value of 0.56 V for Fc/SCE conversion in THF as given in reference 2 and of 0.241 V for SCE/NHE conversion. According to the correlation in eq. (1) this leads to a hydricity of approximately 36 kcal mol^{-1} for **3** in MeCN.

Thermodynamic analysis of the reaction of 4$^-$ with CO_2:

The value for the hydricity of CO_2 in MeCN was taken from reference 3.

**4**$^-$	→	**3** + H^-	$\Delta G^0_{H^-}$ = 36 kcal mol^{-1}
HCO_2^-	→	CO_2 + H^-	$\Delta G^0_{H^-}$ = 44 kcal mol^{-1}
**4**$^-$ + CO_2	→	**3** + HCO_2^-	$\Delta G^0_{H^-}$ = –8 kcal mol^{-1}

*Figure S 1. Thermodynamic scheme to estimate the driving force for hydrogenation of CO_2 by **4**$^-$.*

Thermodynamic analysis of the reaction of 4$^-$ with H^+:

The value for the hydricity of H_2 of MeCN was taken from reference 3 and the pK_a value of $[DBUH]^+$ in MeCN from reference 4.

**4**$^-$	→	**3** + H^-	$\Delta G^0_{H^-}$ = 36 kcal mol^{-1}
H_2	→	H^+ + H^-	$\Delta G^0_{H^-}$ = 79.6 kcal mol^{-1}
$DBUH^+$	→	DBU + H^+	ΔG^0 = 32.9 kcal mol^{-1}<br>pK_a = 24.1
**4**$^-$ + $DBUH^+$	→	**3** + H_2	$\Delta G^0_{H^-}$ = –11 kcal mol^{-1}

*Figure S 2. Thermodynamic scheme to estimate the driving force for H_2 evolution from $[DBUH]^+$ and **4**$^-$.*

Details on the simulation of the CV data of 3

DigiElch 8 Software was used for the simulation of the CV data applying a Butler-Volmer model for the redox process. The exact simulation parameters can be found in Table S 1and the model in Scheme S 2. The symmetry factor was fixed *α* at 0.5.

*Table S 1. Potential, E^0, associated electron transfer rate constant, k_s, and reaction rate, k_f, used for the simulation of the experimental CV data of **3** depicted in Figure S 8.*

E^0 [V]	k_s [cm s^{-1}]	k_f [s^{-1}]	D [cm^2 s^{-1}]
–2.62	0.013	1.4	$1.3 \bullet 10^{-5}$

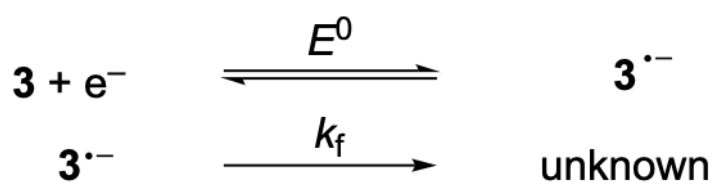

*Scheme S 2. Reaction scheme for the simulation of the CV data of **3**.*

Experimental section

General details

If not otherwise stated, manipulations of air-sensitive reagents were carried out by means of common Schlenk-type techniques involving the use of a dry argon or nitrogen atmosphere or performed in a glovebox manufactured either by MBraun or Innovative Technologies, Inc..

Instrumentation

^{1}H, ^{7}Li{^{1}H}, ^{11}B, ^{11}B-BS (BS = background suppression), ^{11}B{^{1}H}, and ^{13}C{^{1}H} NMR spectra were recorded at 25 °C in C_6D_6, $CDCl_3$, THF-d_8, THF-H_8, DMSO-d_6, or CD_3CN on either a Bruker Avance NEO 500 spectrometer, a Bruker Avance Nanobay 400 spectrometer, a Bruker Avance 300 spectrometer, or an Agilent/Varian MR 400 spectrometer. ^{1}H and ^{13}C{^{1}H} NMR chemical shifts were calibrated against the residual solvent signal internally (δ(^{1}H): C_6D_6 7.16 ppm, $CDCl_3$ 7.26 ppm, THF-d_8 3.58 ppm, DMSO-d_6 2.50 ppm, and CD_3CN 1.94 ppm; δ(^{13}C): C_6D_6 128.06 ppm, $CDCl_3$ 77.16 ppm, THF-d_8 67.21 ppm, DMSO-d_6 39.52 ppm, and CD_3CN 1.32 ppm), and ^{11}B chemical shifts externally against $Et_2O \bullet BF_3$ δ(^{11}B): THF-d_8 0.0 ppm. ^{11}B NMR spectra were recorded with the zg30 pulse sequences and the ^{11}B-BS NMR spectra with the zgbs pulse sequence as implemented in the Bruker Avance software. The zgbs pulse sequence leads to integrals that do not reflect the concentration of the compounds in the mixture.

IR spectra were recorded with a Nicolet IR100 FTIR spectrometer as drop-cast films from solutions in THF or CH_2Cl_2 onto KCl plates. Melting points were measured in sealed glass capillaries under a nitrogen atmosphere using a Mel-Temp apparatus and are reported without correction. Elemental analyses (C, H, N, S) were performed using a Flash 2000 Elemental Analyzer (Thermo Scientific). The UV/Vis data were recorded with a BWTek Exemplar LS spectrometer with a BWTek deuterium/tungsten light source that is connected via fiber optic cables to the glovebox.

Electrochemical measurements were recorded with a Gamry Instruments Reference 600 apparatus in the glovebox at room temperature. A common three electrode setup was used with a glassy carbon working electrode (GC: CH Instruments, ALS Japan; A = 7.1 mm^2, all other setups: GC: CH Instruments, ALS Japan; A = 2.0 mm^2), a platinum wire as a counter electrode and a silver wire in silver nitrate solution (I = 0.01 M) as a reference electrode. For measurements in THF, $^nBu_4NBPh_4$ (I = 0.1 M) or $^nHex_4NBAr^F_4$ (I = 0.2 M) was used as the conducting salt with a reactant concentration of ca. 1 mM (Ar^F = 3,5-$(F_3C)_2C_6H_3$). For measurements in difluorobenzene, $^nHex_4NBAr^F_4$ was used as the conducting salt, I = 0.2 M, with a reactant concentration of ca. 2 mM. All data were referenced internally *vs*. the $Fc^{+|0}$ redox potential (Fc = Fe(η^5-C_5H_5)$_2$). i*R* compensation was performed by the positive feedback method, as implemented in the PHE200 software provided by Gamry.

Chemicals

2-Bromothiophenol was purchased from Aaron Chemicals, *tert*-butyl chloride, anhydrous $AlCl_3$, *n*-butyllithium (2.5 M solution in hexanes), Li(AlH_4), $^nBu_4NCl$, benzophenone, $NaBPh_4$, HCl (2.0 M

solution in Et_2O), $Na(Et_3BH)$ 1.0 M solution in toluene, and $^{13}CO_2$ (99.0 atom % ^{13}C, <3 atom % ^{18}O) were purchased from Sigma-Aldrich and used as received. DBU was purchased from TCI Deutschland GmbH and used as received. Potassium was purchased from abcr GmbH and used as received. CO_2 (4.5 or 5.3 grade) was purchased from Praxair (Linde) and used as received. All solvents used for chemical synthesis were dried using a Grubbs-type solvent purification system (manufactured by Innovative Technology, Inc.) or an MBraun solvent purification system. THF used for electrochemical measurements was dried over Na/benzophenone, distilled, and stored over 4 Å molecular sieves. Difluorobenzene was dried over CaH_2, distilled, and stored over 4 Å molecular sieves. All solvents were stored over 4 Å molecular sieves under a nitrogen atmosphere prior to use. Dimesitylfluoroborane[5] and $^{n}Hex_4NBAr^F_4$[6] were synthesized according to known procedures.

Synthetic details

Synthesis of KC_8 as adapted from reference 7: Graphite was dried at 350 °C under vacuum for several hours and left under vacuum overnight at room temperature. A glass centrifuge tube was charged with graphite (0.201 g, 16.1 mmol, 7.7 eq.) and potassium (1 eq., 2.1 mmol, 0.083 g) was added piece by piece while stirring with a flame-dried glass rod until a dark golden solid is formed, which took approximately 30 min.

Synthesis of [DBUH][BPh_4] as adapted from reference 8: DBU (0.100 g, 0.657 mmol) was dissolved in 20 mL CH_2Cl_2 and HCl (2.0 M solution in Et_2O, 0.32 mL, 0.65 mmol) was added. The reaction mixture was stirred for 1 h and then solid $NaBPh_4$ (0.22 g, 0.65 mmol) was added. The mixture was stirred for another hour and filtered. Removal of the volatiles from the filtrate CH_2Cl_2 afforded a white solid that was recrystallized from a mixture of CH_2Cl_2 (22 mL) and hexanes (4 mL) at –35 °C. (0.25 g, 0.54 mmol, 83%) The NMR data of [DBUH][BPh_4] match those reported in literature.[8]

^1H NMR (DMSO-d$_6$, 300.13 MHz): δ 9.47 (br, 1H, N-*H*), 7.25–7.10 (m, 8H, ^{Ph}*o*-C*H*), 6.93 (t, $J(^1H,^1H)$ = 7.3 Hz, 8H, ^{Ph}*m*-C*H*), 6.79 (t, $J(^1H,^1H)$ = 7.1 Hz, 4H, ^{Ph}*p*-C*H*), 3.55–3.49 (m, 2H, HN-CH_2), 3.44 (t, $J(^1H,^1H)$ = 5.9 Hz, 2H, N-CH_2), 3.23 (t, $J(^1H,^1H)$ = 5.8 Hz, 2H, N-CH_2), 2.65–2.59 (m, 2H, C-CH_2), 1.89 (q, $J(^1H,^1H)$ = 5.8 Hz, 2H, HN-CH_2-CH_2), 1.71–1.50 ppm (m, 6H, C-CH_2-CH_2-CH_2-CH_2-CH_2-N).

$^{11}B\{^1H\}$ NMR (DMSO-d$_6$, 96.29 MHz): δ –6.4 ppm (s, *B*Ph_4).

$^{13}C\{^1H\}$ NMR (DMSO-d$_6$, 75.48 MHz): δ 135.5 (s, 2C, ^{Ph}*o*-*C*H), 125.2 (s, 2C, ^{Ph}*m*-*C*H), 121.5 (s, ^{Ph}*p*-*C*H), 53.4 (s, C-$(CH_2)_4$-*C*H_2-N), 47.9 (s, NH-$(CH_2)_2$-*C*H_2-N), 37.6 (s, NH-*C*H_2), 31.7 (s, C-*C*H_2), 28.2 (s, C-$(CH_2)_2$-*C*H_2-$(CH_2)_2$-N), 25.9 (s, C-CH_2-*C*H_2), 23.3 (s, C-CH_2-*C*H_2-$(CH_2)_3$-N), 18.8 ppm (s, *C*H_2-CH_2-NH).

Synthesis of 2-(*tert*-butylthio)bromobenzene, 1. To a solution of 2-bromothiophenol (8.70 g, 46.0 mmol) in *tert*-butyl chloride (50 mL) was added anhydrous $AlCl_3$ (100 mg, 0.750 mmol) and the mixture was stirred for 8 h at room temperature. An additional equivalent of anhydrous $AlCl_3$ (100 mg, 0.750 mmol) was then added and the reaction mixture was stirred for another 16 h. Afterwards, H_2O (100 mL) was added and the mixture extracted with Et_2O (3 × 50 mL). The combined organic extracts were washed with sat. aq. $NaHCO_3$ (20 mL) and brine (20 mL). The organic layer was dried over Na_2SO_4, the mixture filtered, and the solvent was removed from the filtrate under reduced pressure to give **1** as a colorless liquid (9.60 g, 39.2 mmol, 85%). The analytical data of **1** matched those reported in the literature.[9]

One-pot synthesis of (2-(*tert*-butylthio)phenyl)dimesitylborane, 3. A solution of *n*-butyllithium (2.5 M solution in hexanes, 6.00 mL, 15 mmol) was added dropwise to a solution of 2-(*tert*-butylthio)bromobenzene (**1**) (3.68 g, 15.0 mmol) in 60 mL of Et_2O at 0 °C. After stirring at 0 °C for 1.5

h, dimesitylfluoroborane (4.02 g, 15.0 mmol) was added slowly and the resulting reaction mixture was stirred at room temperature for 19 h. The reaction mixture was filtered, and the filtrate was concentrated to a volume of ca. 15 mL. Cooling of this solution to −35 °C for 2 days afforded **3** as pale-yellow crystals, which were separated from the mother liquor by decantation and dried under vacuum (3.10 g, 7.48 mmol). The remaining mother liquor was concentrated to a volume of ca. 5 mL and stored at −35 °C for 5 days to yield another crop of **3** as pale-yellow crystals, which were separated from the mother liquor by decantation and dried under vacuum (2.61 g, 6.30 mmol). Combined yield of **3**: 5.71 g (13.8 mmol, 92% yield). The analytical data of **3** matched those reported for Route 1 (see manuscript).

Synthesis of tetra-*n*-butylammonium (2-(*tert*-butylthio)phenyl)hydridodimesityl-borate, ($^{n}Bu_4N$)4. A solution of $^{n}Bu_4NCl$ (50.7 mg, 0.183 mmol) in dichloromethane (2 mL) was added dropwise to a solution of [Li(THF)$_3$]**4** (116 mg, 0.183 mmol) in dichloromethane (2 mL) resulting in the precipitation of a fine white solid. After stirring at room temperature for 1 h the mixture was filtered, and the filtrate was evaporated to dryness. The remaining colorless oil was triturated with hexanes (2 mL) and the resulting white solid, ($^{n}Bu_4N$)**4**, was dried under vacuum. Yield: 119 mg (0.181 mmol, 99%).

^{1}H NMR ($CDCl_3$, 498.10 MHz): δ 7.38−7.31 (m br, 1H, ^{Ar}C*H*), 7.18−7.11 (m br, 1H, ^{Ar}C*H*), 6.90−6.78 (m br, 2H, ^{Ar}C*H*), 6.51 (s, 4H, ^{Mes}C*H*), 3.99 (q, $^{1}J(^{11}B,^{1}H)$ = 80.4 Hz, 1H, B*H*), 2.78−2.62 (m, 8H, ^{nBu}NCH_2), 2.16 (s, 6H, $^{p\text{-}CH3}$C*H*), 1.94 (br s, 12H, $^{o\text{-}CH3}$C*H*), 1.46−1.37 (m, 8H, ^{nBu}CH_2), 1.35 (s, 9H, ^{tBu}CH_3), 1.33−1.26 (m, 8H, ^{nBu}CH_2), 0.98 ppm (t, $^{3}J(^{1}H,^{1}H)$ = 7.3 Hz, 12H, ^{nBu}CH_3).

^{1}H{^{11}B} NMR ($CDCl_3$, 498.10 MHz): δ 7.38−7.31 (m br, 1H, ^{Ar}C*H*), 7.18−7.11 (m br, 1H, ^{Ar}C*H*), 6.90−6.78 (m br, 2H, ^{Ar}C*H*), 6.51 (s, 4H, ^{Mes}C*H*), 3.99 (s, 1H, B*H*), 2.78−2.62 (m, 8H, ^{nBu}NCH_2), 2.16 (s, 6H, $^{p\text{-}CH3}$C*H*), 1.94 (br s, 12H, $^{o\text{-}CH3}$C*H*), 1.46−1.37 (m, 8H, ^{nBu}CH_2), 1.35 (s, 9H, ^{tBu}CH_3), 1.33−1.26 (m, 8H, ^{nBu}CH_2), 0.98 ppm (t, $^{3}J(^{1}H,^{1}H)$ = 7.3 Hz, 12H, ^{nBu}CH_3).

^{13}C{^{1}H} NMR ($CDCl_3$, 125.26 MHz): δ 171.6 (br, ^{Ar}*C*B), 159.4 (br, ^{Mes}*C*B), 143.2 (s, ^{Mes}*C*), 140.1 (s, ^{Ar}*CS*), 136.4 (s, ^{Mes}C), 134.8 (s, ^{Ar}*C*H), 129.4 (s, ^{Ar}*C*H), 127.4 (s, ^{Ar}*C*H), 125.4 (s, ^{Mes}*C*H), 121.6 (s, ^{Ar}CH), 58.6 (s, ^{nBu}N*C*H_2), 45.7 (s, ^{tBu}*C*(CH_3)$_3$), 32.5 (s, ^{tBu}*C*H_3), 25.1 (s, o-*C*H_3), 24.2 (s, ^{nBu}*C*H_2), 21.3 (s, p-*C*H_3), 19.8 (s, ^{nBu}*C*H_2), 13.9 ppm (s, ^{nBu}*C*H_3).

^{11}B NMR ($CDCl_3$, 160.36 MHz): δ −13.8 ppm (d, $^{1}J(^{11}B,^{1}H)$ = 80.4 Hz).

^{11}B{^{1}H} NMR ($CDCl_3$, 160.36 MHz): δ −13.8 ppm (s).

Elemental analysis: Calculated (%) for $C_{44}H_{72}BNS$, C 80.32, H 11.03, N 2.13, S 4.87; found, C 79.82, H 10.95, N 2.16, S 4.88.

IR (CH_2Cl_2 drop-cast film, KCl plate): 2319 cm^{-1} ($\tilde{\nu}$(BH)); computed for **4**$^{-}$ (M06-2X/cc-pVTZ, scaled by 0.946): 2356 cm^{-1} ($\tilde{\nu}$(BH)).

Reaction of 4^{-} with protons.

30 mg (1 eq., 72 μmol) of **3** and Na(Et_3BH) (1 eq., 72 μL, 1.0 M solution in toluene, 72 μmol) were dissolved in 2 mL of THF. After 1 h of stirring at room temperature, the volatiles were removed *in vacuo*. A saturated solution of [DBUH][BPh_4] (*c* = 52 mM, 0.026 mmol) in 0.5 mL THF-d_8 was prepared and 5 mg of Na**4** (0.01 mmol) were added. The resulting mixture was transferred to a J-Young NMR tube, and an ^{11}B-BS (background suppression) NMR spectrum was recorded 23 h after the addition of [DBUH][BPh_4].

Reaction of 4^{-} with CO_2.

I. Reaction of Li4 with CO_2. [Li(THF)$_3$]**4** (5.1 mg, 8.0 µmol) was dissolved in THF (0.5 mL) under a nitrogen atmosphere. The nitrogen atmosphere was replaced by purging with CO_2 gas which led to the immediate formation of a colorless micro-crystalline precipitate. The mother liquor was decanted, and the precipitate was washed with benzene (3 × 0.3 mL). Evaporation of the combined THF and benzene solutions yielded analytically pure **3** (3.1 mg, 7.20 µmol). The precipitate was dissolved in D_2O and NMR spectra were recorded confirming the formation of lithium formate (Figure S 30 - Figure S 33).

II. Reaction of ($^{n}Bu_4N$)4 with CO_2. ($^{n}Bu_4N$)**4** (8.4 mg, 13 µmol) was dissolved in either C_6D_6 (0.5 mL) or $CDCl_3$ (0.5 mL) or THF (0.5 mL) under a nitrogen atmosphere to give a colorless solution. The nitrogen atmosphere was replaced by purging with CO_2 gas and the colorless solutions were investigated by NMR spectroscopy within 10 min. Selective and quantitative formation of **3** alongside ($^{n}Bu_4N$)(HCO_2) was observed by ^{1}H, $^{13}C\{^{1}H\}$, and ^{11}B NMR spectroscopy of the reaction mixtures (Figure S 34 - Figure S 36).

III. Reaction of [DBUH][BPh_4], 3, KC_8 and $^{13}CO_2$. A J-Young NMR tube was charged with a suspension of **3** (30.0 mg, 72.4 µmol, 1 eq.) and [DBUH][BPh_4] (1 eq., 34.2 mg, 72.4 µmol) in 0.5 mL of dry, degassed THF-d_8 inside a glovebox. The mixture was frozen in liquid nitrogen, and the atmosphere was removed *in vacuo*. The tube was taken out of the liquid N_2, warmed to room temperature, and the headspace was filled with gaseous $^{13}CO_2$ to an overpressure of 0.2 bar. The tube was sealed and the mixture was shaken for at least 15 min to saturate the THF solution with CO_2. Subsequently, a suspension of KC_8 (2 eq., 19.6 mg, 0.145 mmol) in 0.1 mL of THF-d_8 was added slowly in a glovebox. The tube was resealed and immediately frozen in liquid nitrogen again, the atmosphere of the NMR tube was removed under vacuum, the tube warmed to room temperature, and the headspace of the tube was filled again with $^{13}CO_2$, as described above. The NMR tube was left for 10 min and then the volatiles were removed *in vacuo*, and the residue was suspended in 0.5 mL D_2O and the mother liquor was decanted after centrifugation at 6000 rpm; this process was repeated three times. The combined D_2O extracts were analyzed by ^{1}H NMR spectroscopy leading to a spectroscopic yield of 90% of $[HCO_2]^{-}$ [Note: 0.147 g (3.58 mmol) of MeCN was used as internal standard]. The remaining solid was dissolved in 0.5 mL THF-d_8 and decantated after centrifugation at 6000 rpm. This process was repeated three times. A spectroscopic yield of 43% for **3** was obtained after extraction in THF-d_8 and determined by ^{1}H NMR spectroscopy using 0.0892 g of CH_2Cl_2 as an internal standard.

Catalytic reaction of 3, KH and CO_2.

KH (321 mg, 8.00 mmol) was placed in a Teflon valve-capped Schlenk tube and THF (4 mL) and a stock solution of **3** in THF (100 µL, 0.0500 M, 5.00 µmol, 0.625 mol%) was added. The mixture was degassed via three freeze-pump-thaw cycles and filled with 1 atm of CO_2 gas. The reaction mixture was stirred at room temperature under 1 atm of CO_2 resulting in the formation of a fine white precipitate. After 1h the excess CO_2 was removed under reduced pressure, and the flask was refilled with N_2 gas. The reaction mixture was quenched by dropwise addition of water (0.5 mL). The volatiles were removed under reduced pressure to yield a colorless solid which was extracted into D_2O (4 × 1 mL). The combined aqueous extract was transferred to a volumetric flask (5.00 mL) and was filled with D_2O to exactly 5.00 mL. Aliquots of 500 µL were used to quantify the amount of potassium formate by ^{1}H NMR spectroscopy using methylsulfonylmethane (4.7 mg, 50.0 µmol) as an internal standard. Yield: 3.16 mmol, 33% calculated for KH; TON = 532; TOF = 532 h^{-1}.

Blank reaction of [DBUH][BPh_4], $^{13}CO_2$ and KC_8

A J-Young NMR tube was charged with a suspension of [DBUH][BPh_4] (34.2 mg, 72.4 µmol) in 0.5 mL of dry, degassed THF-d_8 inside a glovebox. The mixture was frozen in liquid nitrogen, and the atmosphere was removed *in vacuo*. The tube was taken out of the liquid N_2, warmed to room temperature, and the headspace was filled with gaseous $^{13}CO_2$ to an overpressure of 0.2 bar. The tube was sealed and the mixture was shaken for at least 15 min to saturate the THF solution with CO_2. Subsequently, a suspension of KC_8 (2 eq., 19.6 mg, 0.145 mmol) in 0.1 mL of THF-d_8 was added slowly in a glovebox. The tube was resealed and immediately frozen in liquid nitrogen again, the atmosphere of the NMR tube was removed under vacuum, the tube warmed to room temperature, and the headspace of the tube was filled again with $^{13}CO_2$, as described above. The experiment was done twice with two different workup procedures:

a) After 3 h, a ^1H NMR spectrum and $^{13}C\{^1H\}$ NMR spectrum of the THF-d_8 suspension were recorded showing partial consumption of [DBUH][BPh_4] and the formation of hydrogen. The $^{13}C\{^1H\}$ NMR data showed only the resonance of $^{13}CO_2$ (Figure S 45).

b) After 10 min, all volatiles were removed *in vacuo*, and the residue was suspended in 0.5 mL D_2O and the mother liquor was decanted off after centrifugation at 6000 rpm; this process was repeated three times. The combined D_2O extracts were analyzed by ^1H NMR spectroscopy and $^{13}C\{^1H\}$ NMR spectroscopy using MeCN as internal standard. No formate formation was observed (Figure S 46).

Blank reaction of KH and CO_2.

KH (321 mg, 8.00 mmol) was placed in a Teflon valve-capped Schlenk tube and THF (4.1 mL) was added. The mixture was degassed via three freeze-pump-thaw cycles and filled with 1 atm of CO_2 gas. The reaction mixture was stirred at room temperature under 1 atm of CO_2. After 1 h the excess CO_2 was removed under reduced pressure, and the flask was refilled with Ar gas. The reaction mixture was quenched by dropwise addition of water (0.5 mL). The volatiles were removed under reduced pressure to yield a colorless solid which was dissolved in 1 mL D_2O and the solution was transferred to an NMR tube to quantify the amount of potassium formate by ^1H NMR spectroscopy using methylsulfonylmethane (10.0 mg, 106 µmol) as an internal standard. Yield: 17 µmol, 0.2% calculated for KH.

X-Ray crystallography

Single-crystal X-ray data were collected on a Bruker D8 Venture diffractometer with a Photon III detector, using $Cu_{K\alpha}$ (micro-focus sealed X-ray tube, λ_{Cu} = 1.54184 Å) or $Mo_{K\alpha}$ radiation (micro-focus sealed X-ray tube, λ_{Mo} = 0.71073 Å). The structures were solved by intrinsic phasing methods (SHELXT), and refinement is based on full-matrix least-squares calculations on F^2 (SHELXL).[10,11] All nonhydrogen atoms were refined anisotropically. Unless specified differently, for CH, idealized bond lengths and angles were used. Calculations were carried out using the ShelXle graphical interface.[12] Molecular structure diagrams were drawn with the program Diamond 4.6.8.[13] Crystallographic data have been deposited with the Cambridge Crystallographic Data Centre. Experimental details, crystal data, and CCDC numbers are summarized in

Table S 2 and

Table S 3. These data can be obtained free of charge from The Cambridge Crystallographic Data Centre via www.ccdc.cam.ac.uk/data_request/cif.

2•OEt_2: One reflection was removed from the refinement as outlier. The Et_2O molecule is disordered over two positions. Thus, SIMU, RIGU, ISOR, and SADI restraints were used to ensure a stable refinement.

3: Some reflections were removed from the refinement as outliers.

$[Li(THF)_4]$**4**: The THF molecules are disordered over two and three positions, respectively. Thus, SIMU, RIGU, and SADI restraints were used to ensure a stable refinement.

($^{n}Bu_4N$)**4**: The ^{n}Bu groups are disordered over two positions. Thus, SIMU and RIGU restraints were used to ensure a stable refinement. The $^{t}BuS(C_6H_4)$ moiety is disordered over two positions. Thus, SIMU, RIGU, and SADI restraints were used to ensure a stable refinement.

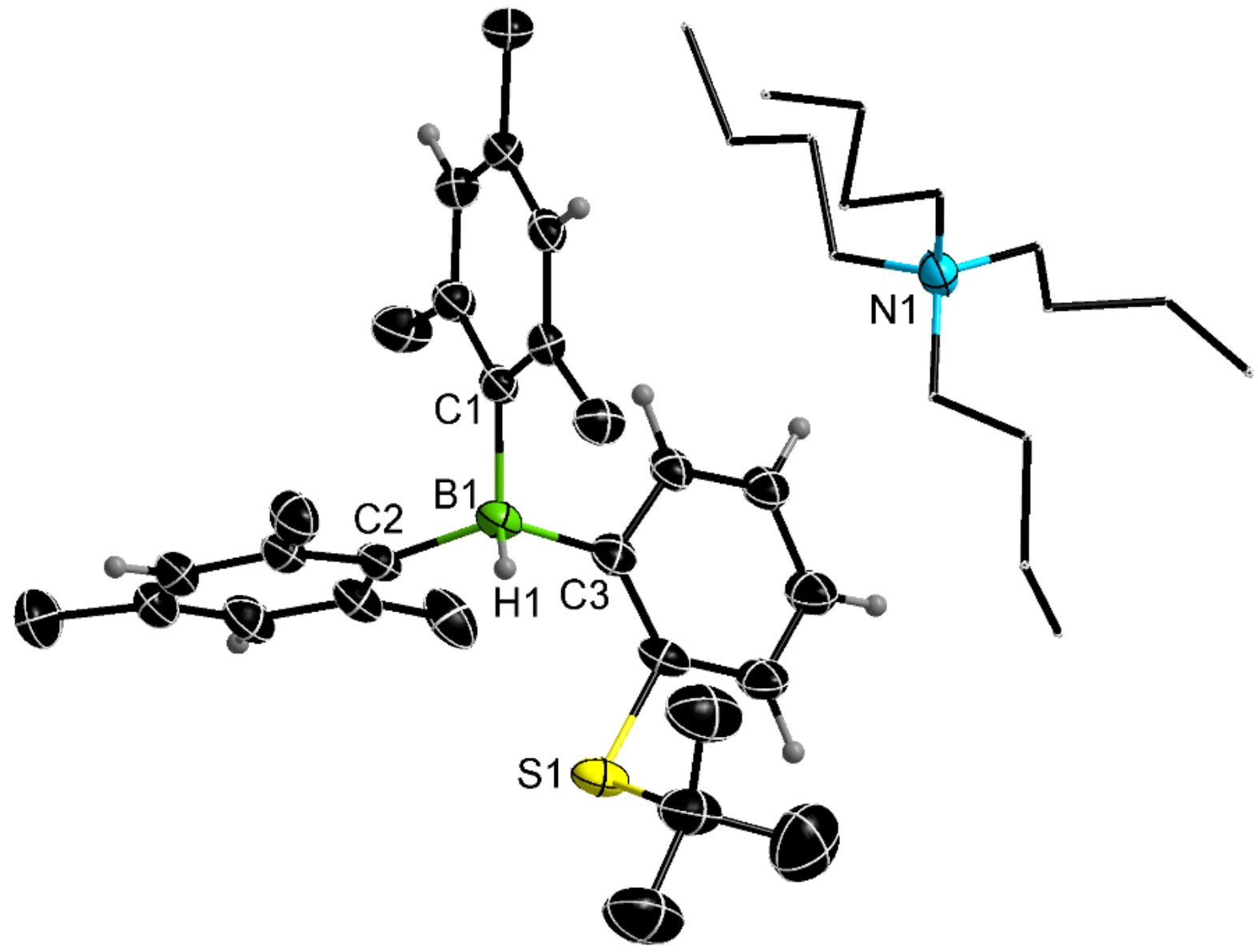

*Figure S 3. Molecular structure of ($^{n}Bu_4N$)**4** with thermal ellipsoid are drawn at a 35% probability level, except for the H atoms which are depicted with arbitrary radii. H atoms of the CH_2 and CH_3 moieties are omitted for clarity, and the C atoms of the n-butyl groups are depicted as wire frame model.*

*Table S 2. Selected crystal data and refinement details of the molecular structures of **2•OEt$_2$** and **3**.*

	**2•OEt$_2$**	**3**
Formula	$C_{28}H_{46}Li_2O_2S_2$	$C_{28}H_{35}BS$
M_W	492.65	414.43
T (K)	173(2)	173(2)
Crystal system	triclinic	triclinic
Space group	$P-1$	$P-1$
a (Å)	10.0540(9)	7.7733(14)
b (Å)	18.4499(18)	12.451(2)
c (Å)	19.6142(18)	13.709(3)
α	63.303(5)	101.380(8)
b	77.775(6)	101.081(8)
γ	78.053(6)	91.051(7)
Volume (Å^3)	3150.6(5)	1274.3(4)
Z	4	2
r (calcd) (Mg m^{-3})	1.039	1.080
m (mm^{-1})	1.664	1.185
F(000)	1072	448
No. of collected reflections	73698	31949
No. of unique reflections	11068	4731
R(int)	0.0915	0.0572
No. of parameters / restraints	825 / 1212	280 / 0
$R1$ ($I > 2s(I)$)	0.0798	0.0548
$wR2$ (all)	0.1832	0.1314
GOF on F^2	1.026	1.023
Largest diff. peak / hole / e Å^{-3}	0.351 / −0.488	0.260 / −0.256
CCDC no.	2562683	2562684

*Table S 3. Selected crystal data and refinement details of the molecular structures of [Li(THF)$_4$]**4** and (^{n}Bu$_4$N)**4**.*

	[Li(THF)$_4$]**4**	(^{n}Bu$_4$N)**4**
Formula	$C_{44}H_{68}BLiO_4S$	$C_{44}H_{72}BNS$
M_W	710.79	657.89
T (K)	173(2)	173(2)
Crystal system	monoclinic	monoclinic
Space group	$P2_1/n$	Cc
a (Å)	14.5241(5)	21.7837(6)
b (Å)	14.4402(5)	12.4049(4)
c (Å)	20.3624(8)	16.3297(4)
α	90	90
b	90.790(2)	107.280(2)
γ	90	90
Volume (Å^3)	4270.2(3)	4213.5(2)
Z	4	4
r (calcd) (Mg m^{-3})	1.106	1.037
m (mm^{-1})	0.959	0.872
F(000)	1552	1456
No. of collected reflections	59730	52595
No. of unique reflections	8354	8073
R(int)	0.0687	0.0504
No. of parameters / restraints	657 / 1234	648 / 836
$R1$ ($I > 2s(I)$)	0.0593	0.0396
$wR2$ (all)	0.1812	0.0903
GOF on F^2	1.049	1.118
Largest diff. peak / hole / e Å^{-3}	0.446 / −0.265	0.192 / −0.175
CCDC no.	2562685	2562686

CV data

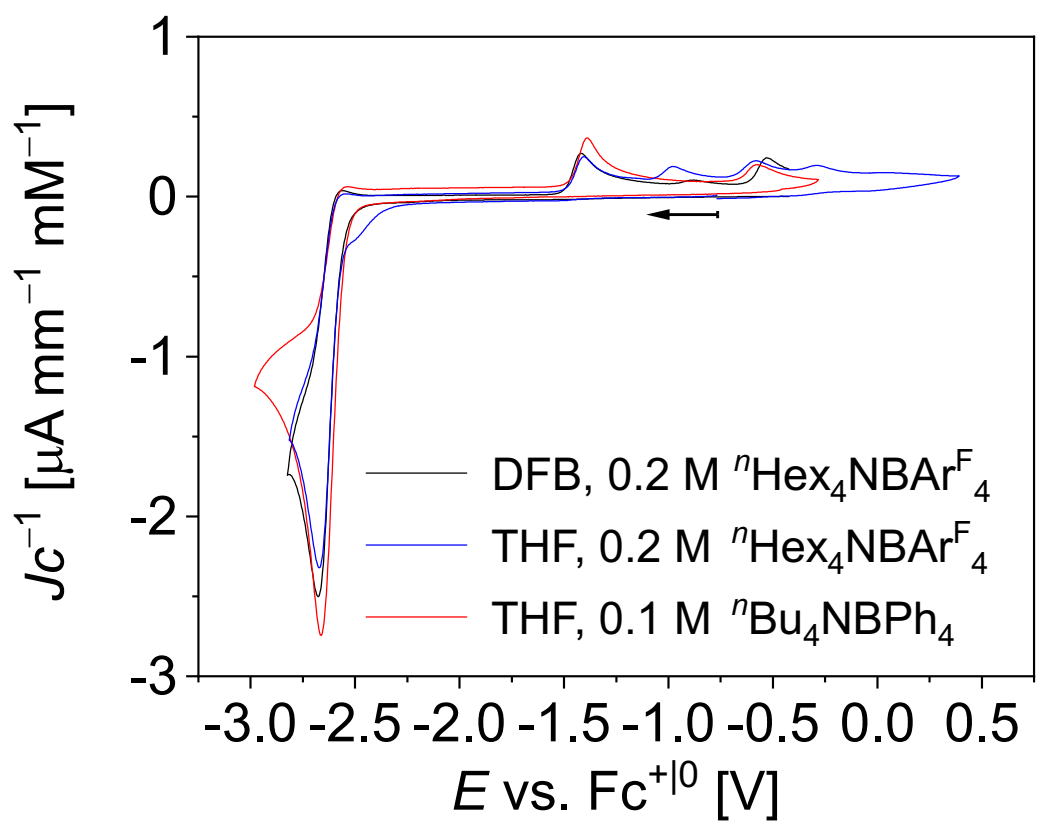

*Figure S 4. Overlay of the concentration normalized CV data of **3** in various solvents and electrolytes, $v = 0.1$ Vs^{-1}. Black: in difluorobenzene with $I = 0.2$ M ^{n}Hex$_4$NBAr^F$_4$, $c_3 = 2$ mM. Blue: in THF with $I = 0.2$ M ^{n}Hex$_4$NBAr^F$_4$, $c_3 = 1$ mM. Red: in THF with $I = 0.1$ M ^{n}Bu$_4$NBPh$_4$, $c_3 = 1$ mM. WE: GC, RE: Ag$^{+|0}$, CE: Pt.*

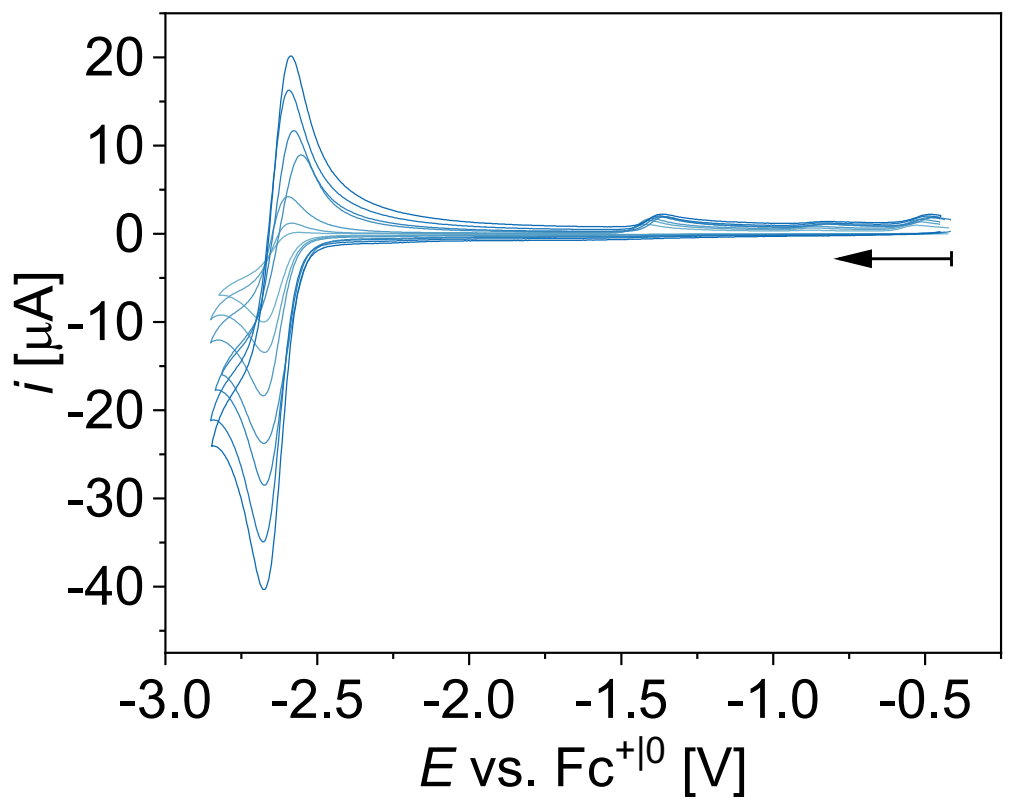

*Figure S 5. CV data of **3** (c_3 = 2 mM) in difluorobenzene, I = 0.2 M ^{n}Hex$_4$NBAr$^F_4$, ν = 0.1, 0.2, 0.4, 0.8, 1.0, 1.5, 2.0 Vs^{-1}. WE: GC, RE: Ag$^{+|0}$, CE: Pt.*

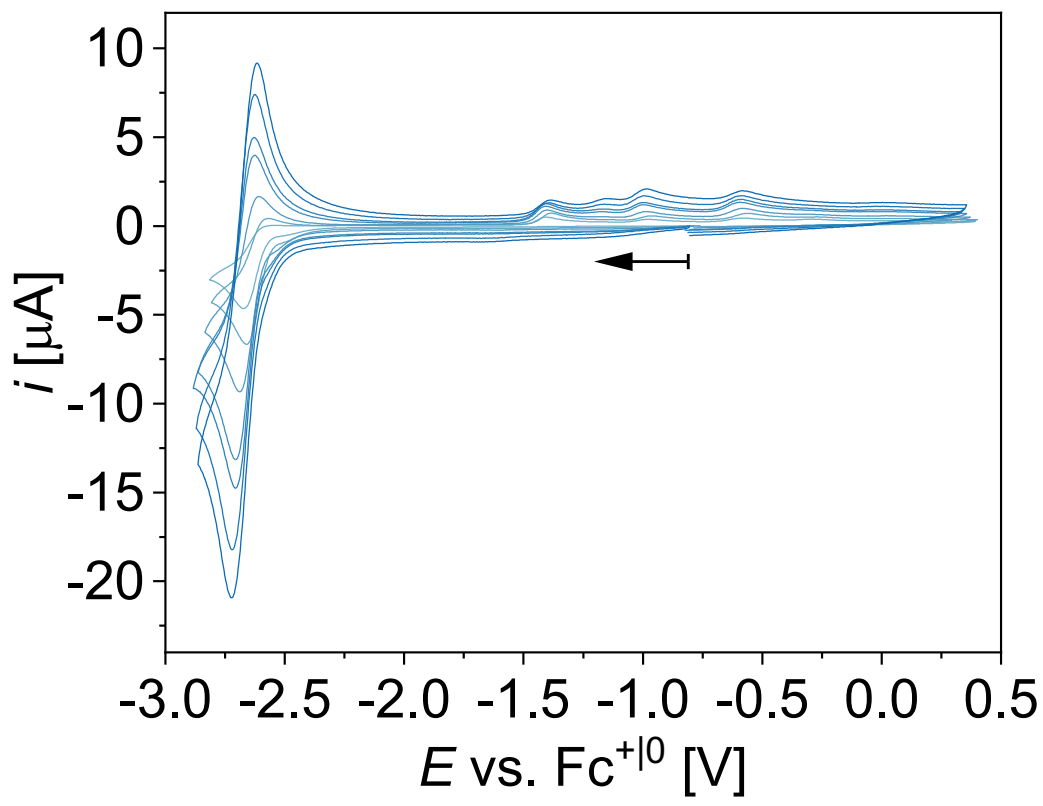

*Figure S 6. CV data of **3** (c_3 = 1 mM) in THF, I = 0.2 M ^{n}Hex$_4$NBAr$^F_4$, ν = 0.1, 0.2, 0.4, 0.8, 1.0, 1.5, 2.0 Vs^{-1}. WE: GC, RE: Ag$^{+|0}$, CE: Pt.*

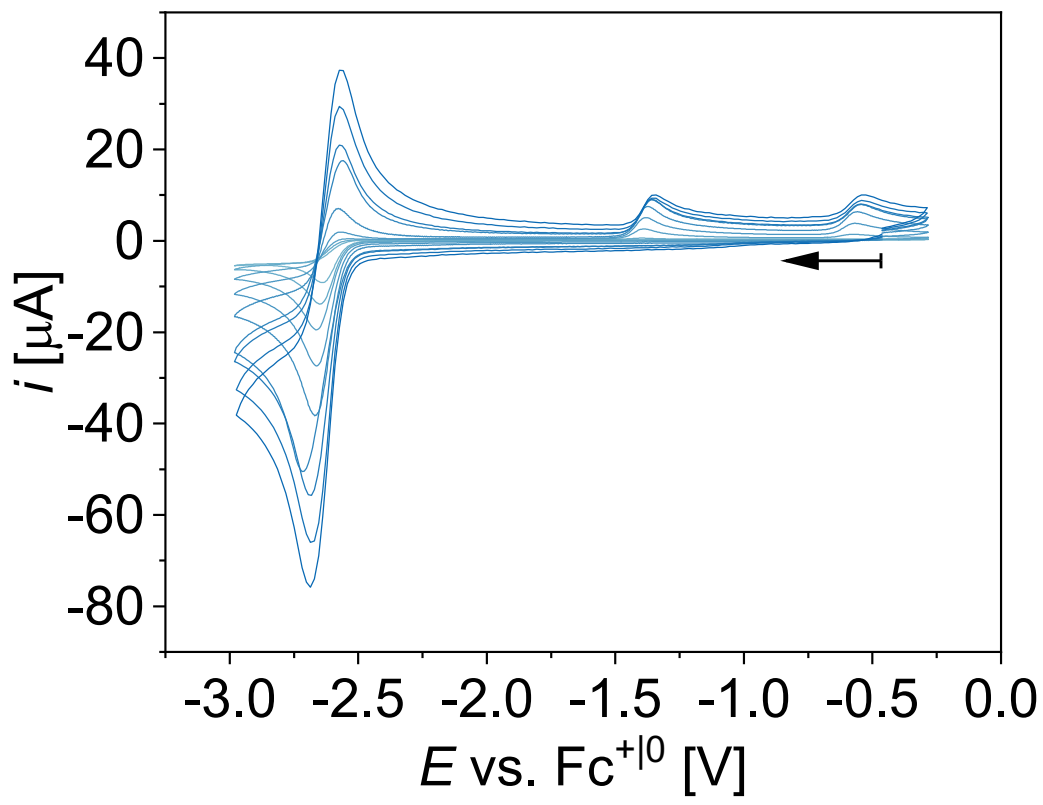

*Figure S 7. CV data of **3** (c_3 = 1 mM) in THF, I = 0.1 M ^{n}Bu$_4$NBPh$_4$, ν = 0.02, 0.05, 0.1, 0.2, 0.4, 0.8, 1.0, 1.5, 2.0 Vs^{-1}. WE: GC, RE: Ag$^{+|0}$, CE: Pt.*

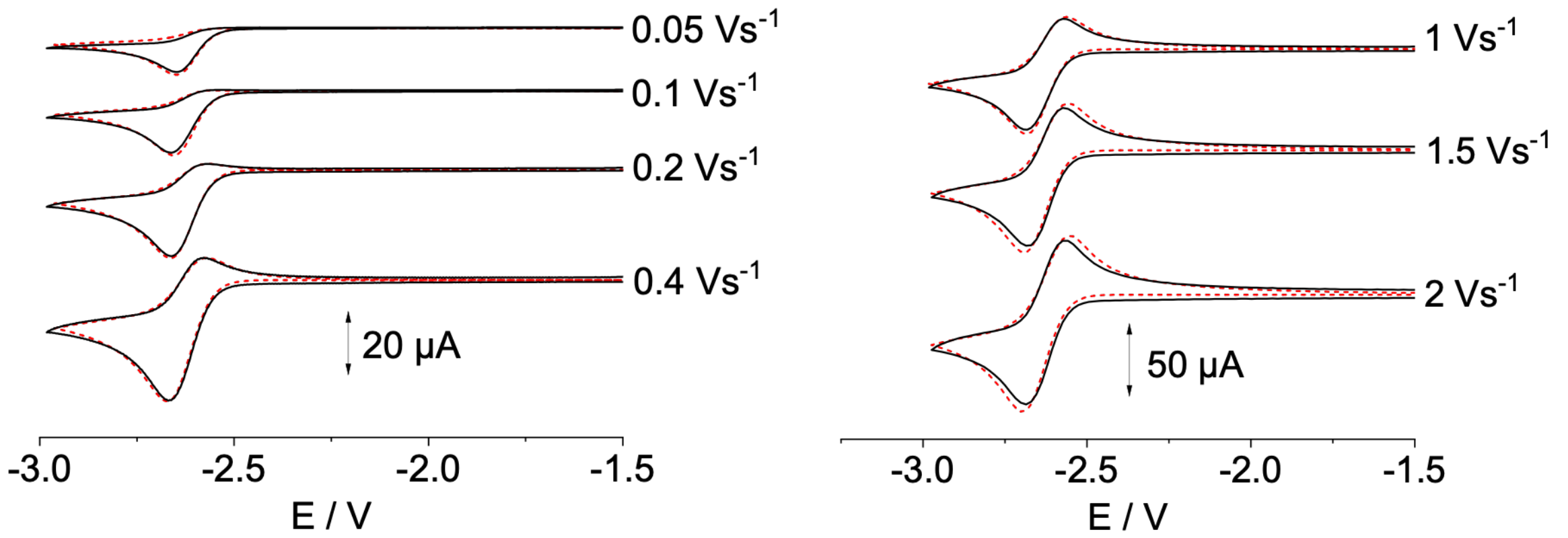

*Figure S 8. Measured (black) and simulated (dotted red) CV data of **3** (ca. 1 mM) in THF, I = 0.1 M ^{n}Bu$_4$NBPh$_4$, v = 0.05, 0.1, 0.2, 0.4, 0.8, 1.0, 1.5, 2.0 Vs^{-1}.*

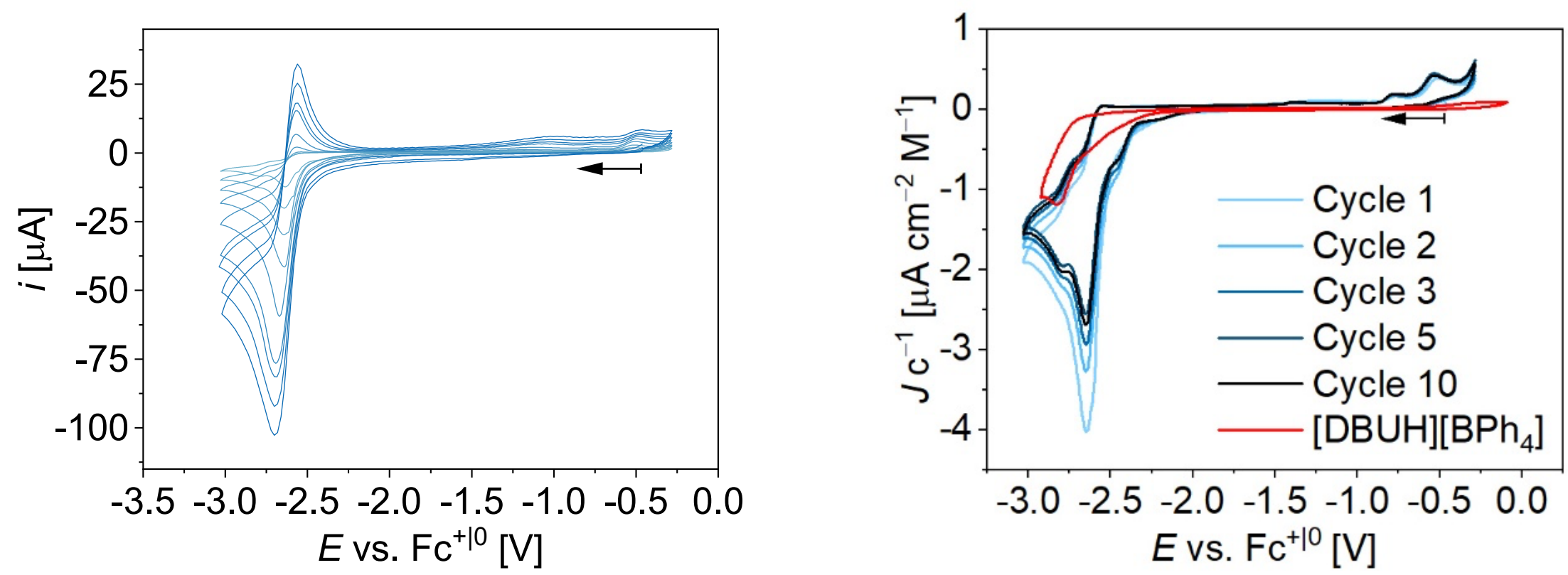

*Figure S 9. CV data of **3** (c_3 = 1mM) and 1 eq. of [DBUH][BPh$_4$] in THF, I = 0.1 M ^{n}Bu$_4$NBPh$_4$, WE: GC, RE: Ag$^{+|0}$, CE: Pt. Left: Scan rate dependence data, v = 0.02, 0.05, 0.1, 0.2, 0.4, 0.8, 1.0, 1.5, 2.0 Vs^{-1}. Right: Multi-cycle CV data of **3** and 1 eq. of [DBUH][BPh$_4$] in blue with an overlay of [DBUH][BPh$_4$] in red, v = 0.1 Vs^{-1}.*

NMR data

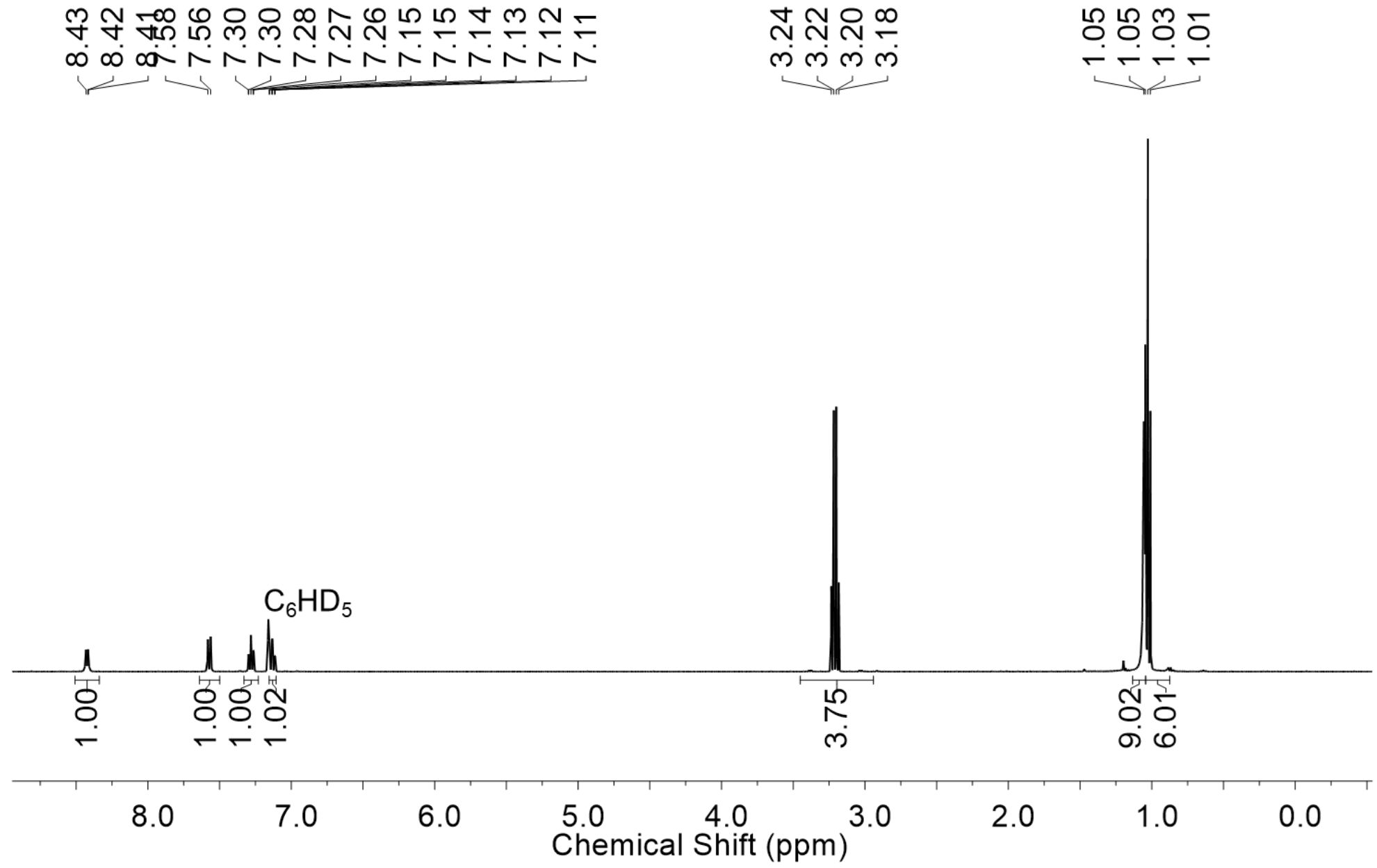

*Figure S 10. ^{1}H NMR spectrum of **2**•OEt_2 in C_6D_6.*

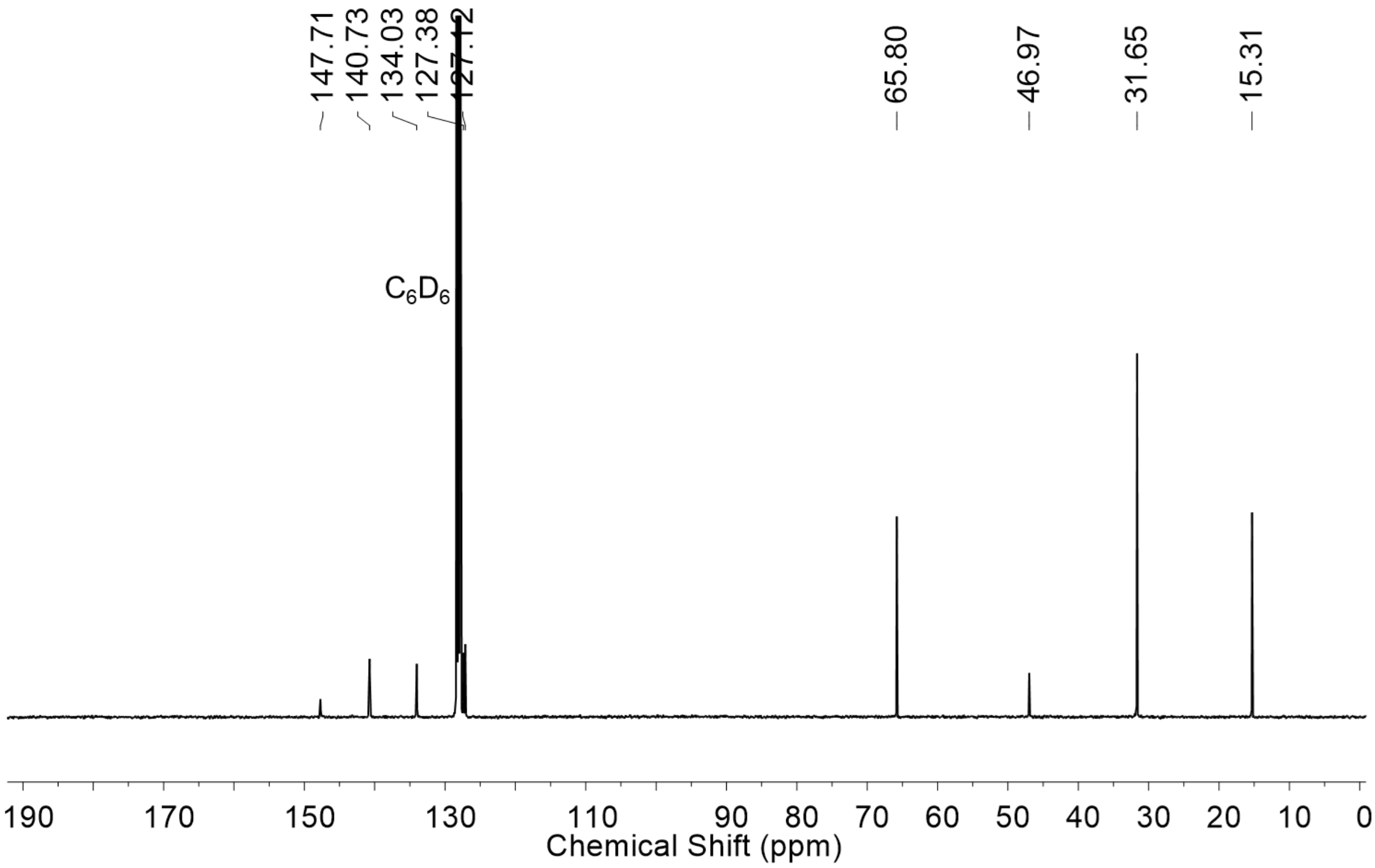

*Figure S 11. $^{13}C\{^{1}H\}$ NMR spectrum of **2**•OEt_2 in C_6D_6.*

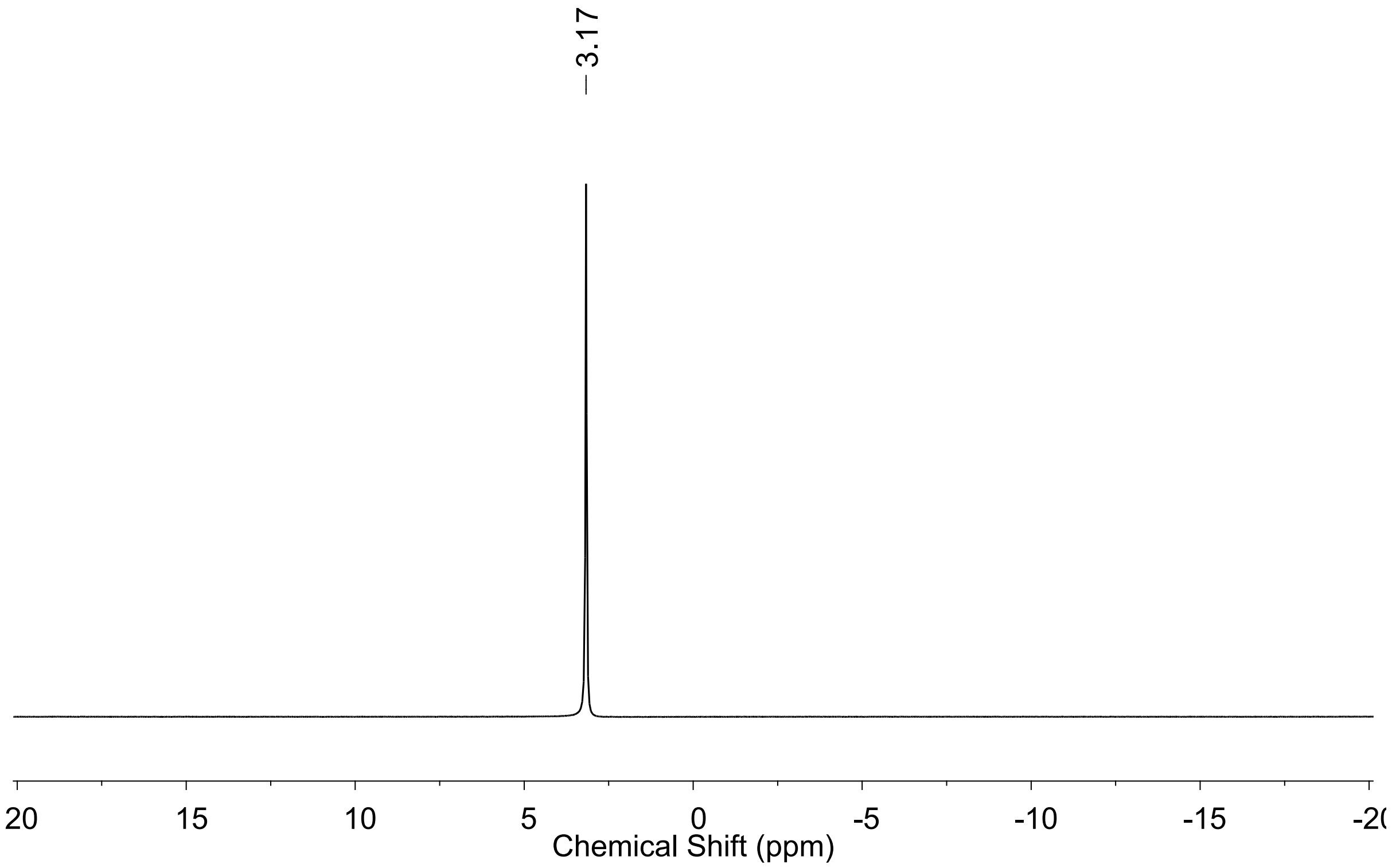

*Figure S 12. $^{7}Li\{^{1}H\}$ NMR spectrum of **2•OEt$_2$** in C_6D_6.*

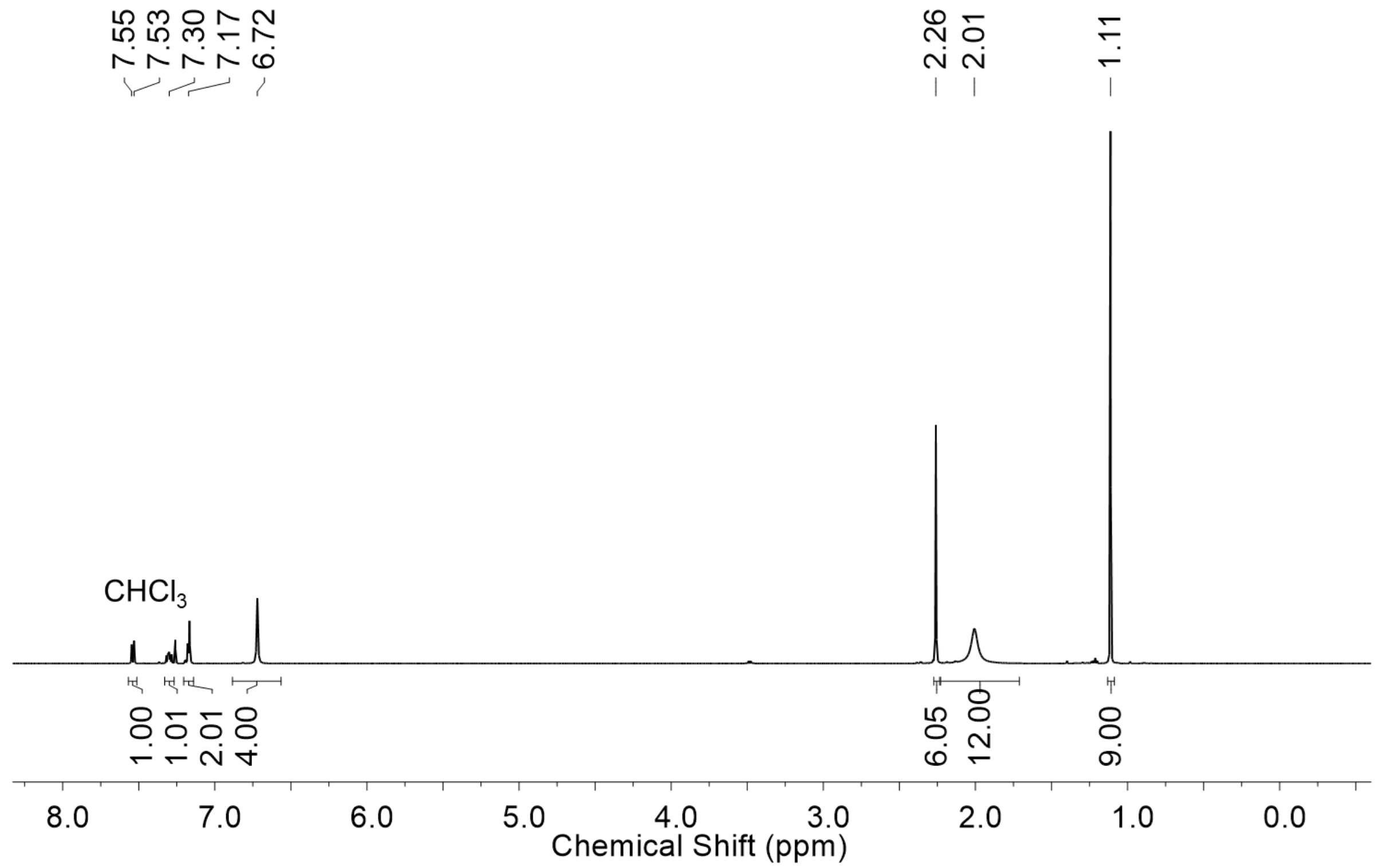

*Figure S 13. ^{1}H NMR spectrum of **3** in $CDCl_3$.*

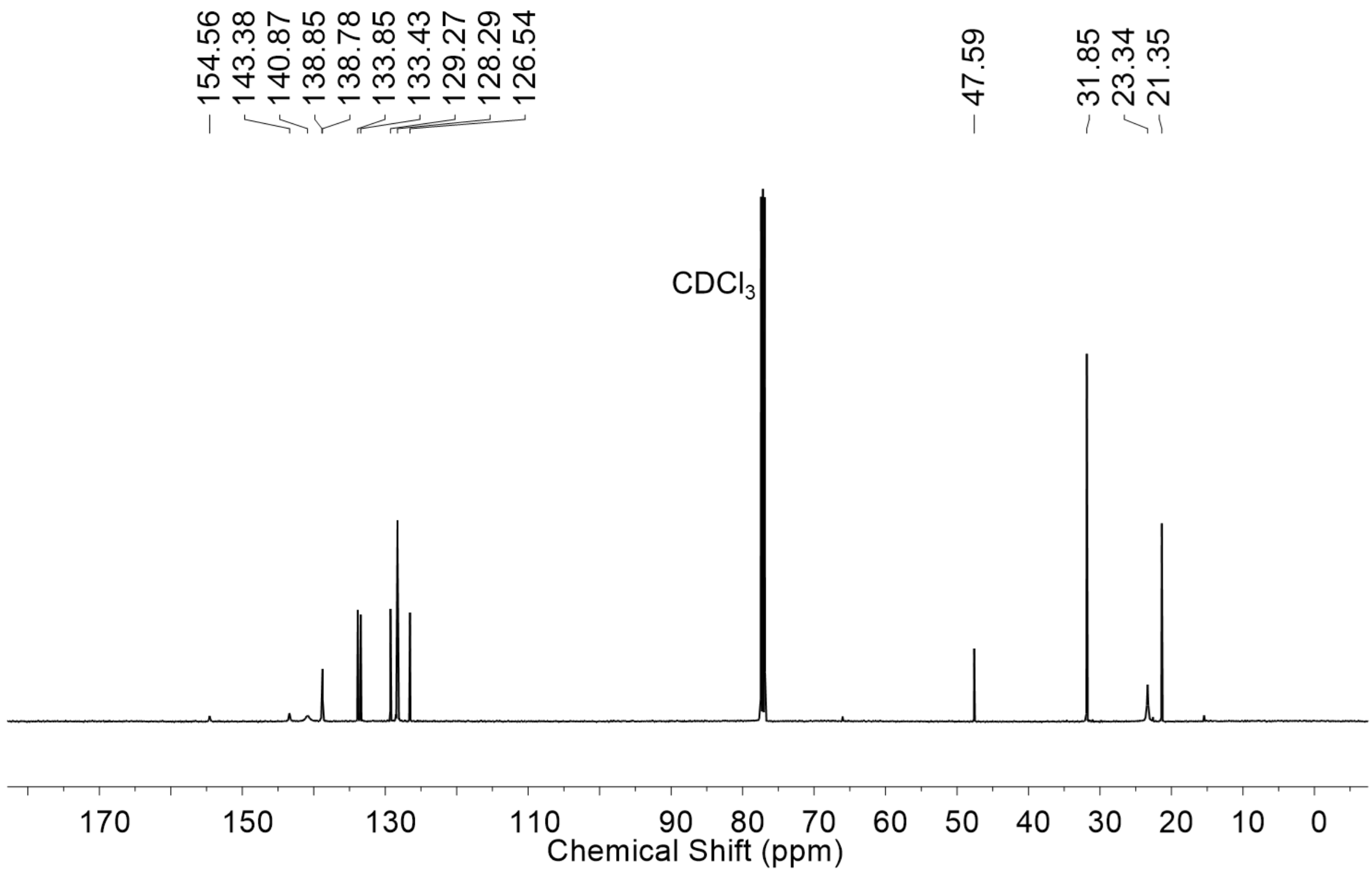

Figure S 14. $^{13}C\{^{1}H\}$ *NMR spectrum of* ***3*** *in* $CDCl_3$.

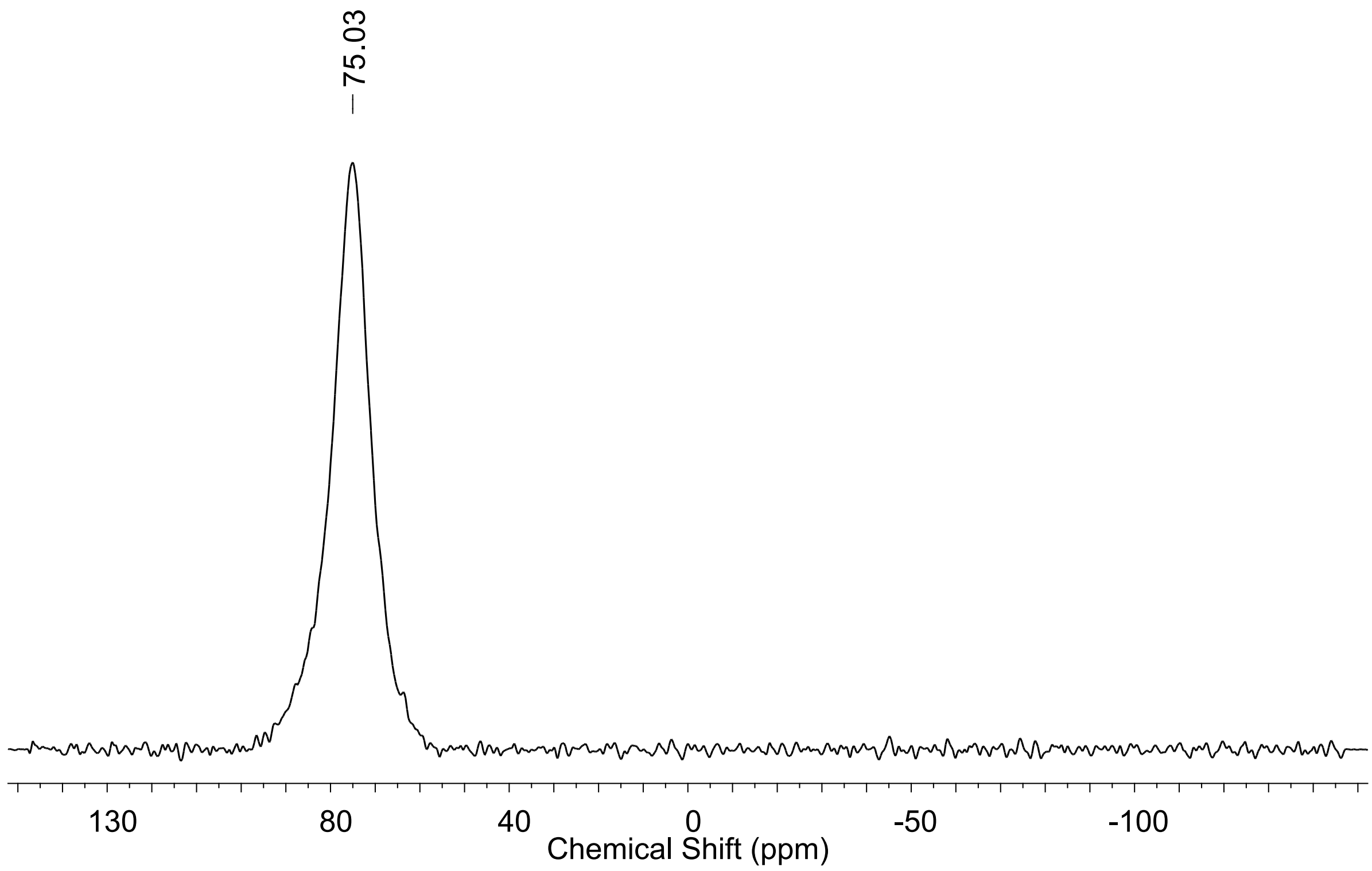

Figure S 15. $^{11}B\{^{1}H\}$ *NMR spectrum of* ***3*** *in* $CDCl_3$.

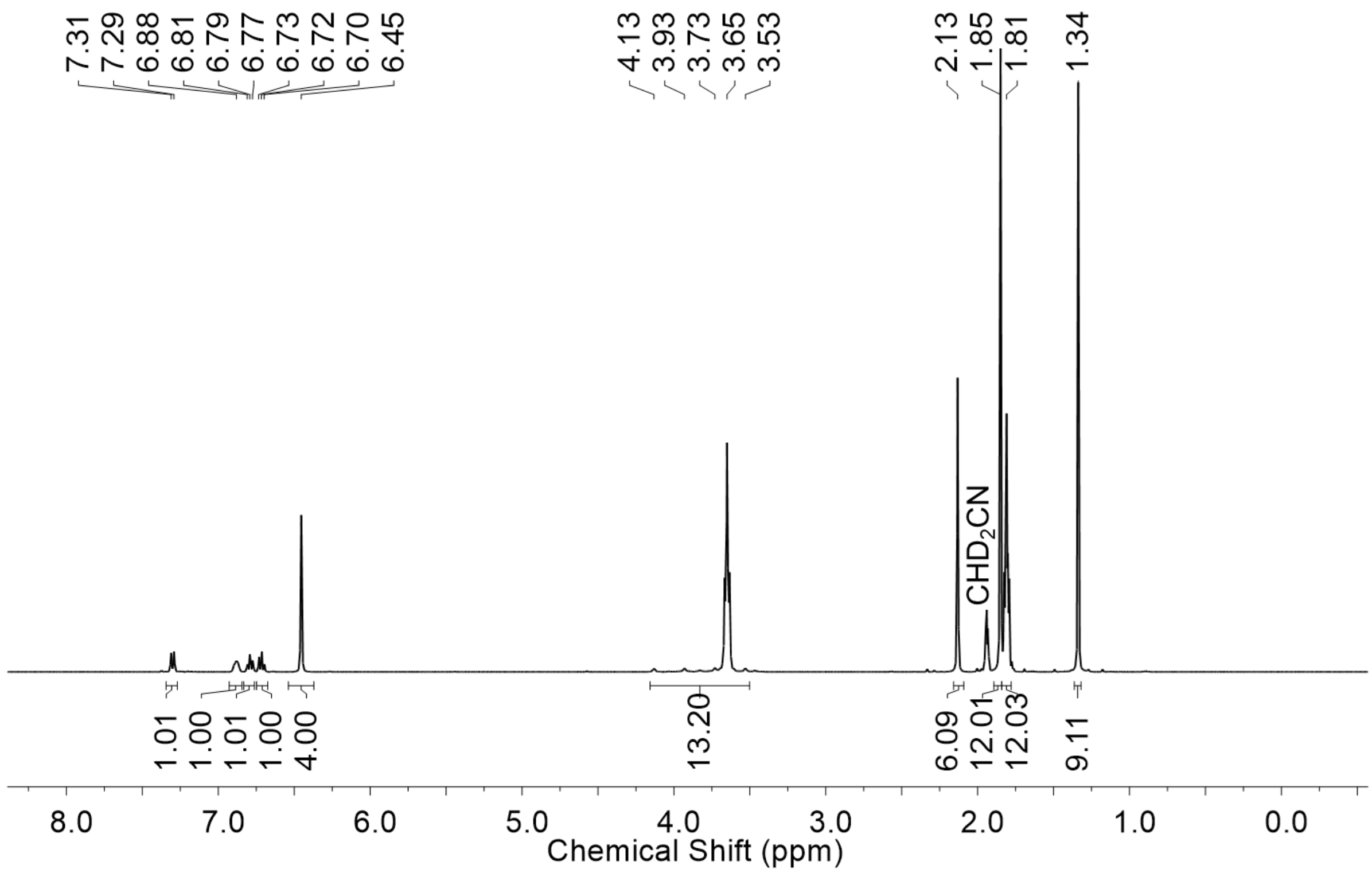

*Figure S 16. ^{1}H NMR spectrum of $[Li(THF)_3]$**4** in CD_3CN.*

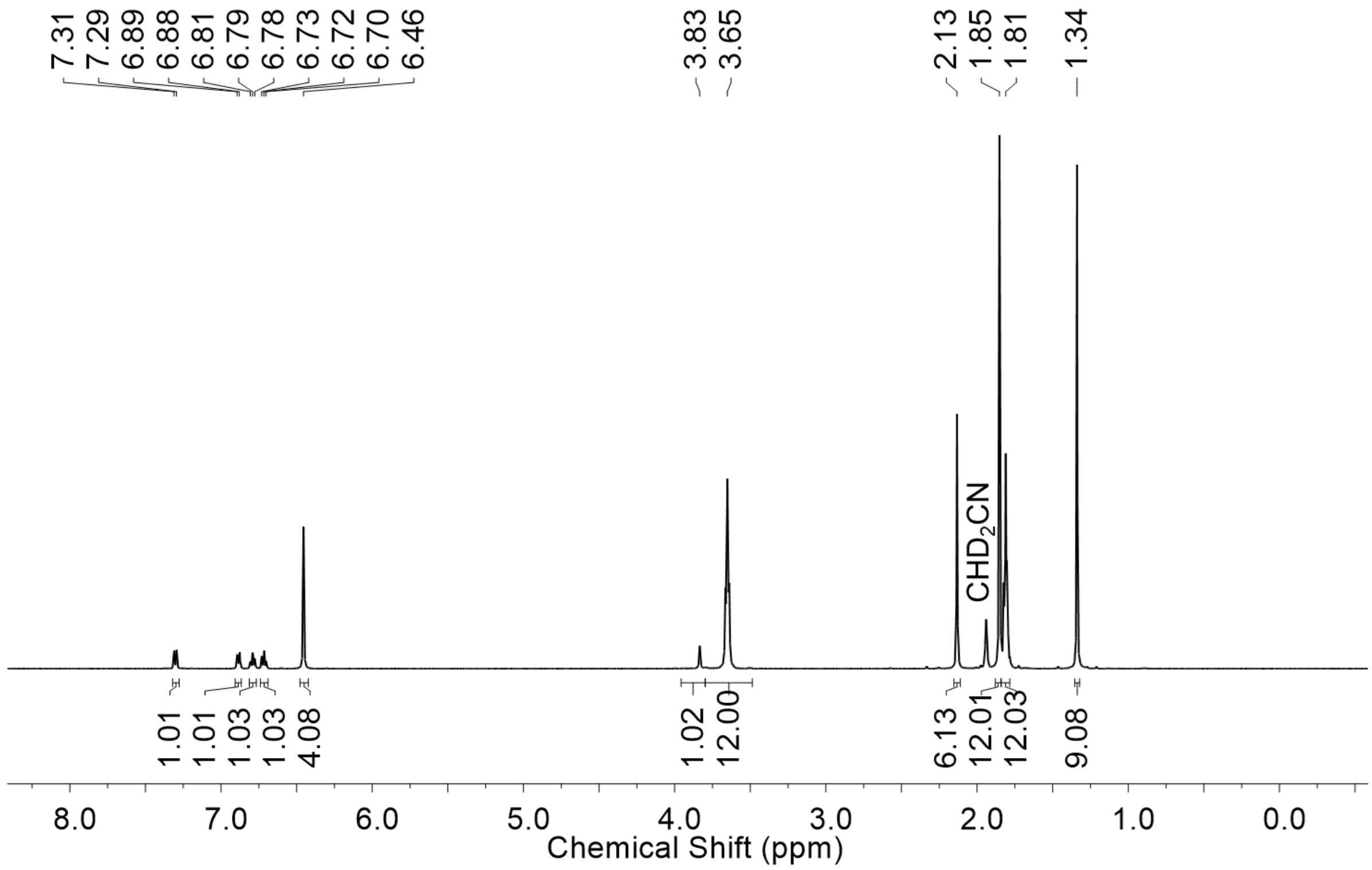

*Figure S 17. $^{1}H\{^{11}B\}$ NMR spectrum of $[Li(THF)_3]$**4** in CD_3CN.*

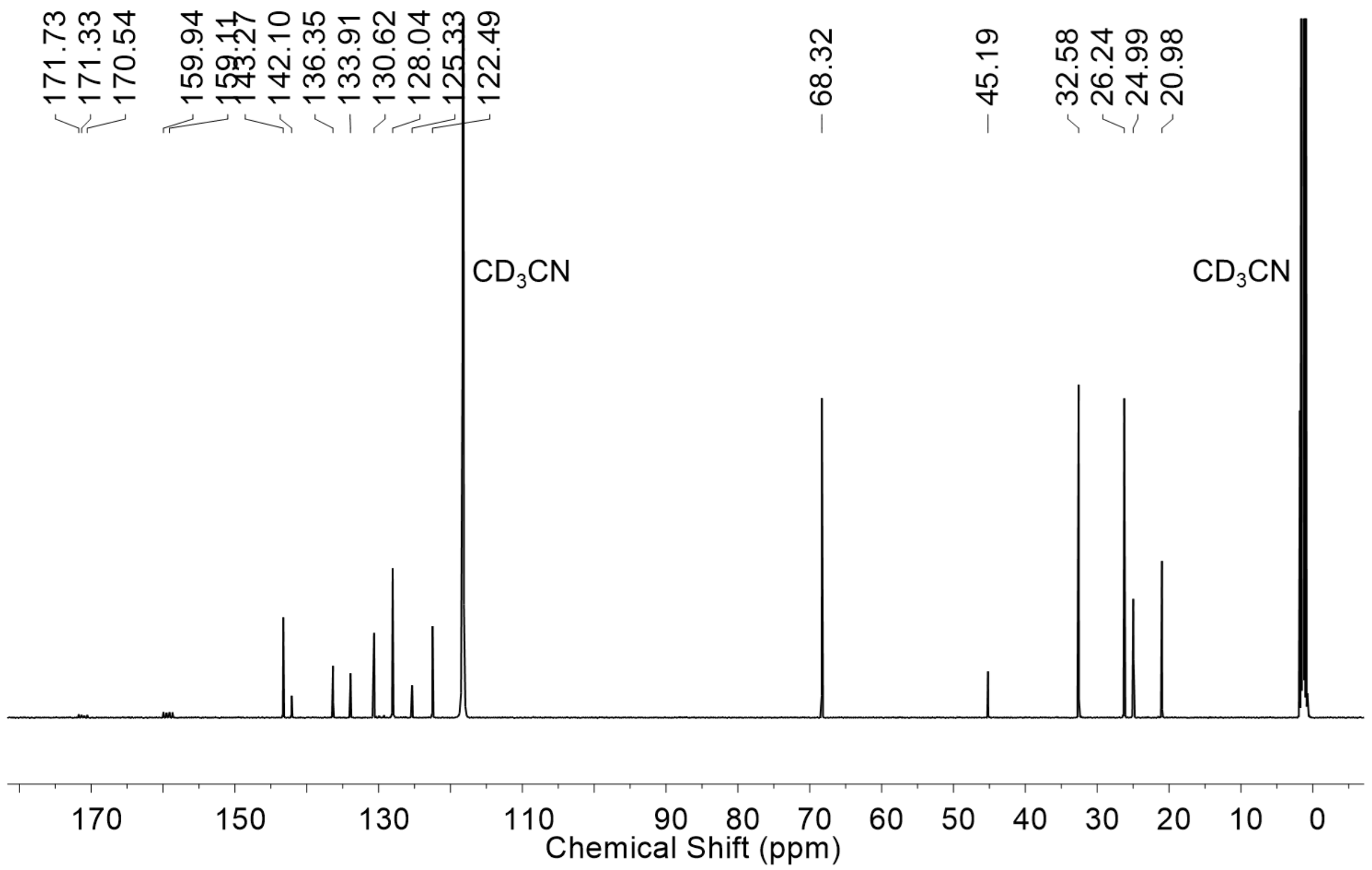

Figure S 18. $^{13}C\{^{1}H\}$ *NMR spectrum of [Li(THF)*$_3$*]**4** in* CD_3CN.

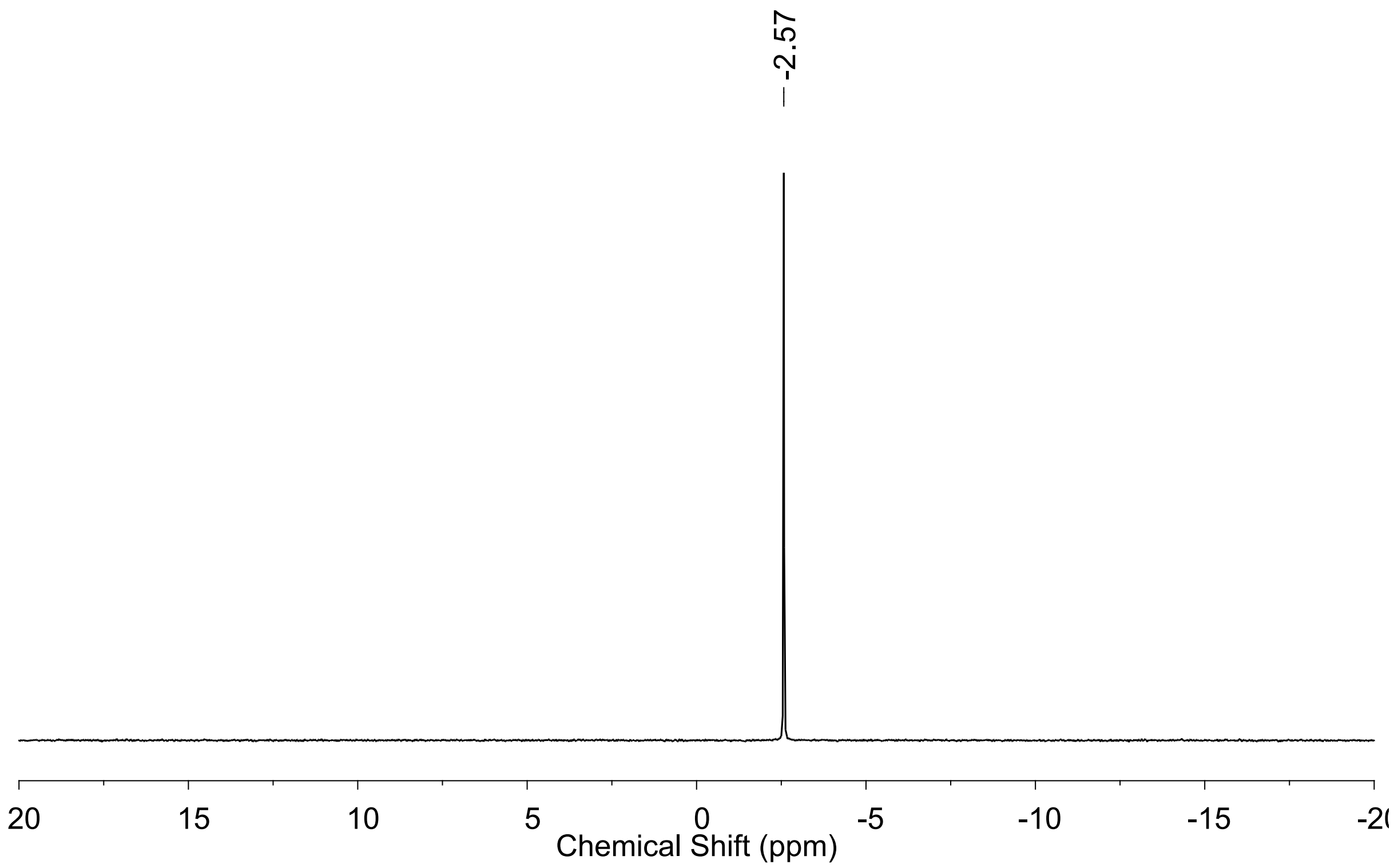

Figure S 19. $^{7}Li\{^{1}H\}$ *NMR spectrum of [Li(THF)*$_3$*]**4** in* CD_3CN.

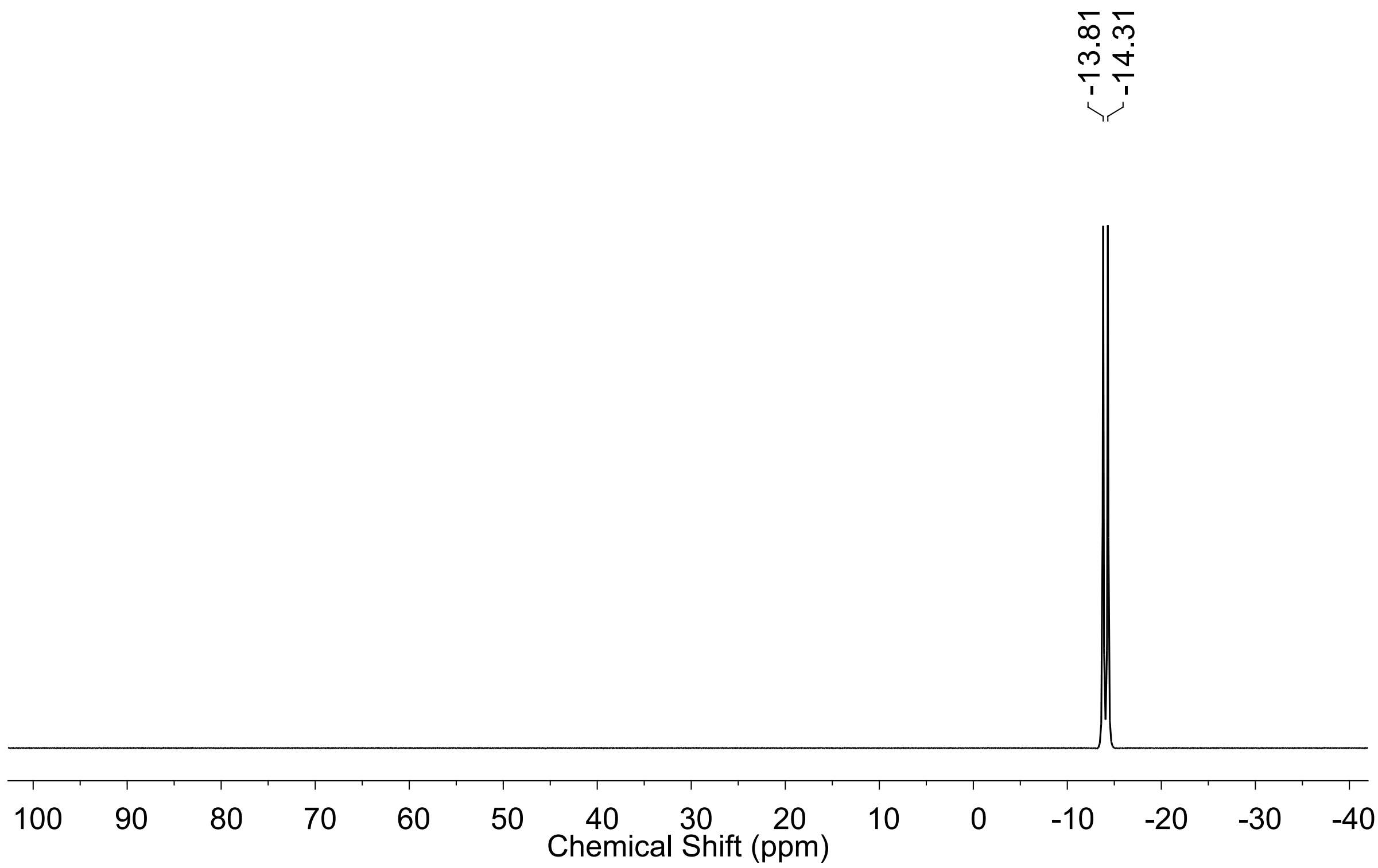

Figure S 20. ^{11}B *NMR spectrum of* $[Li(THF)_3]$***4*** *in* CD_3CN.

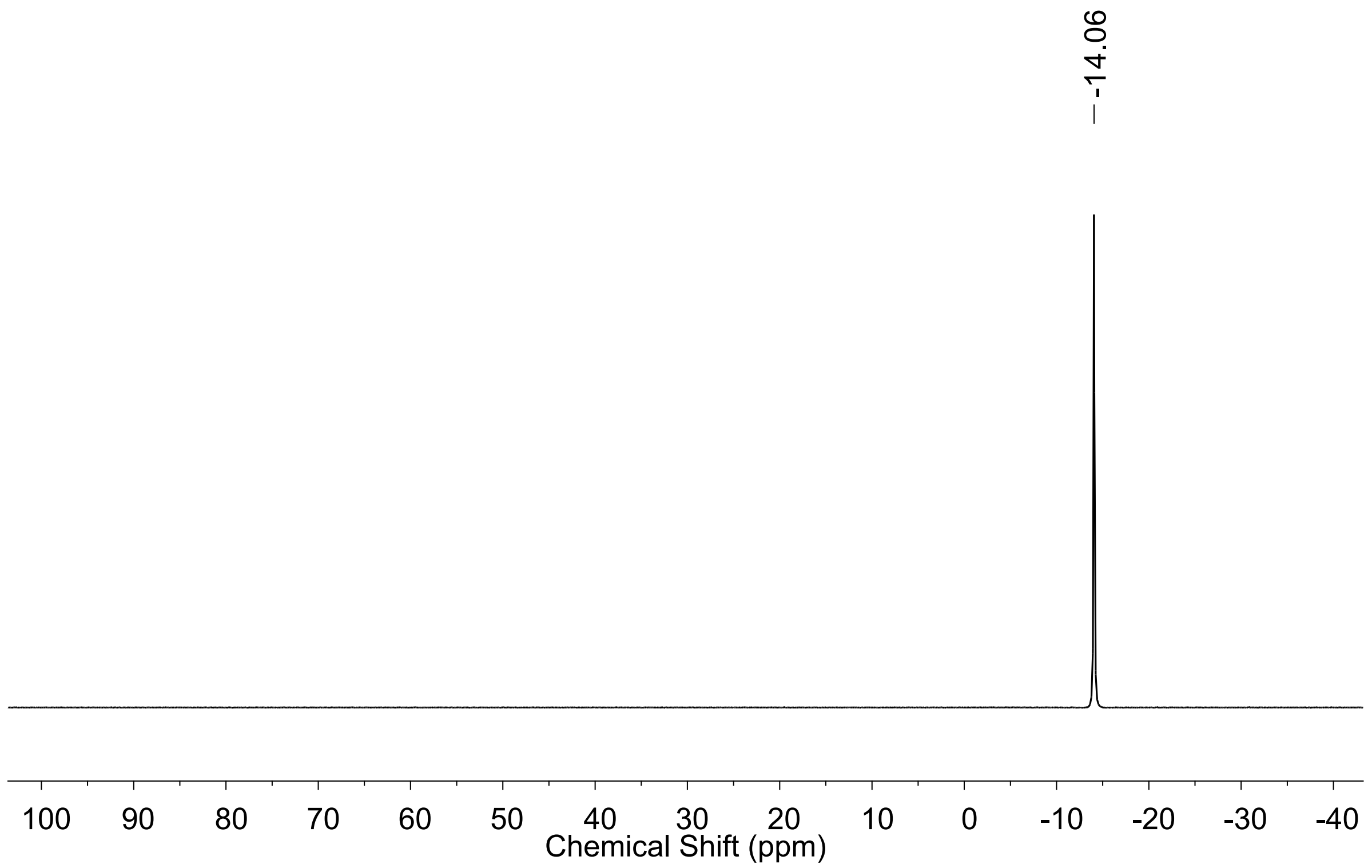

Figure S 21. $^{11}B\{^1H\}$ *NMR spectrum of* $[Li(THF)_3]$***4*** *in* CD_3CN.

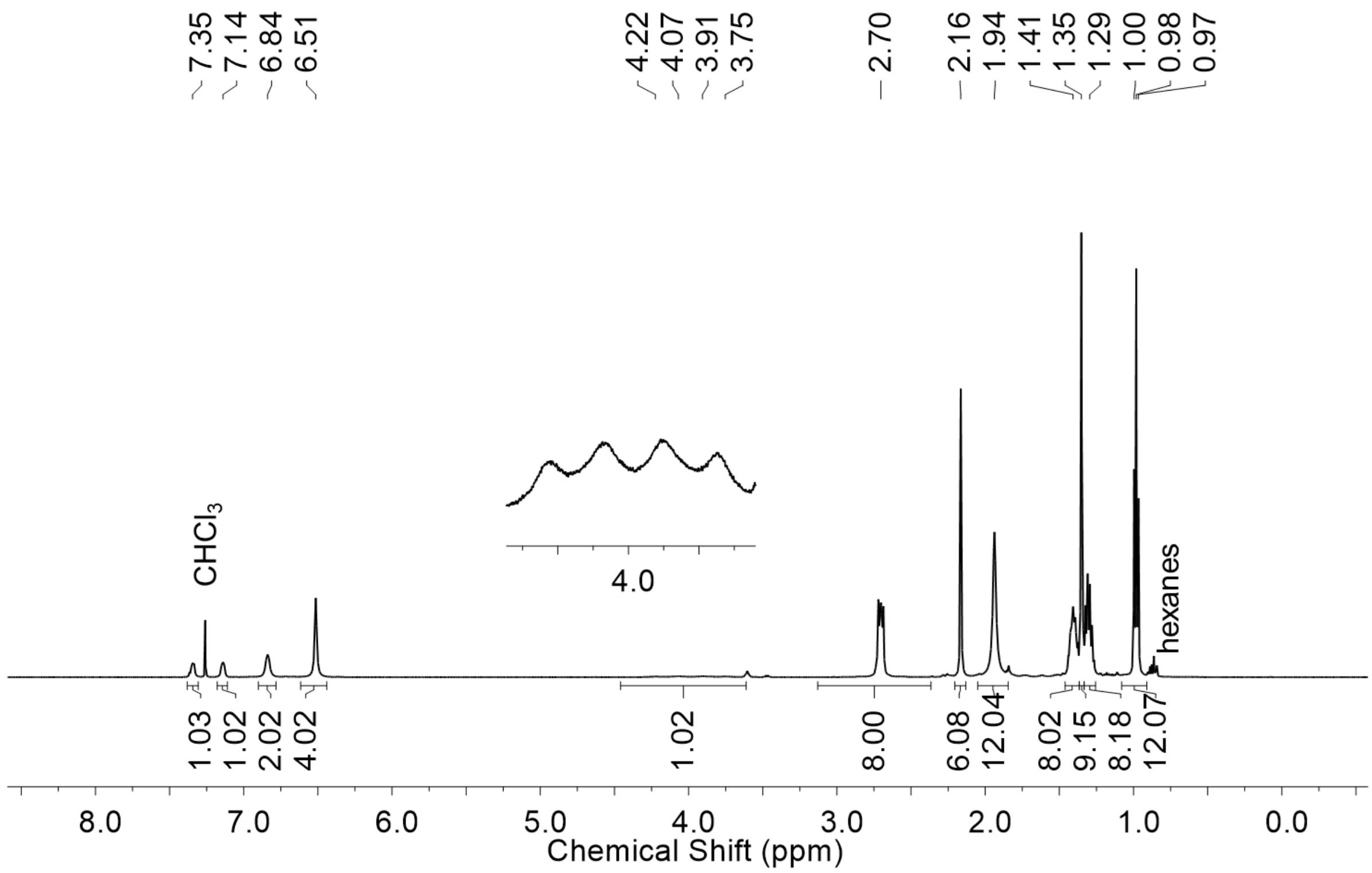

*Figure S 22. ^{1}H NMR spectrum of (^{n}Bu$_4$N)**4** in CDCl$_3$.*

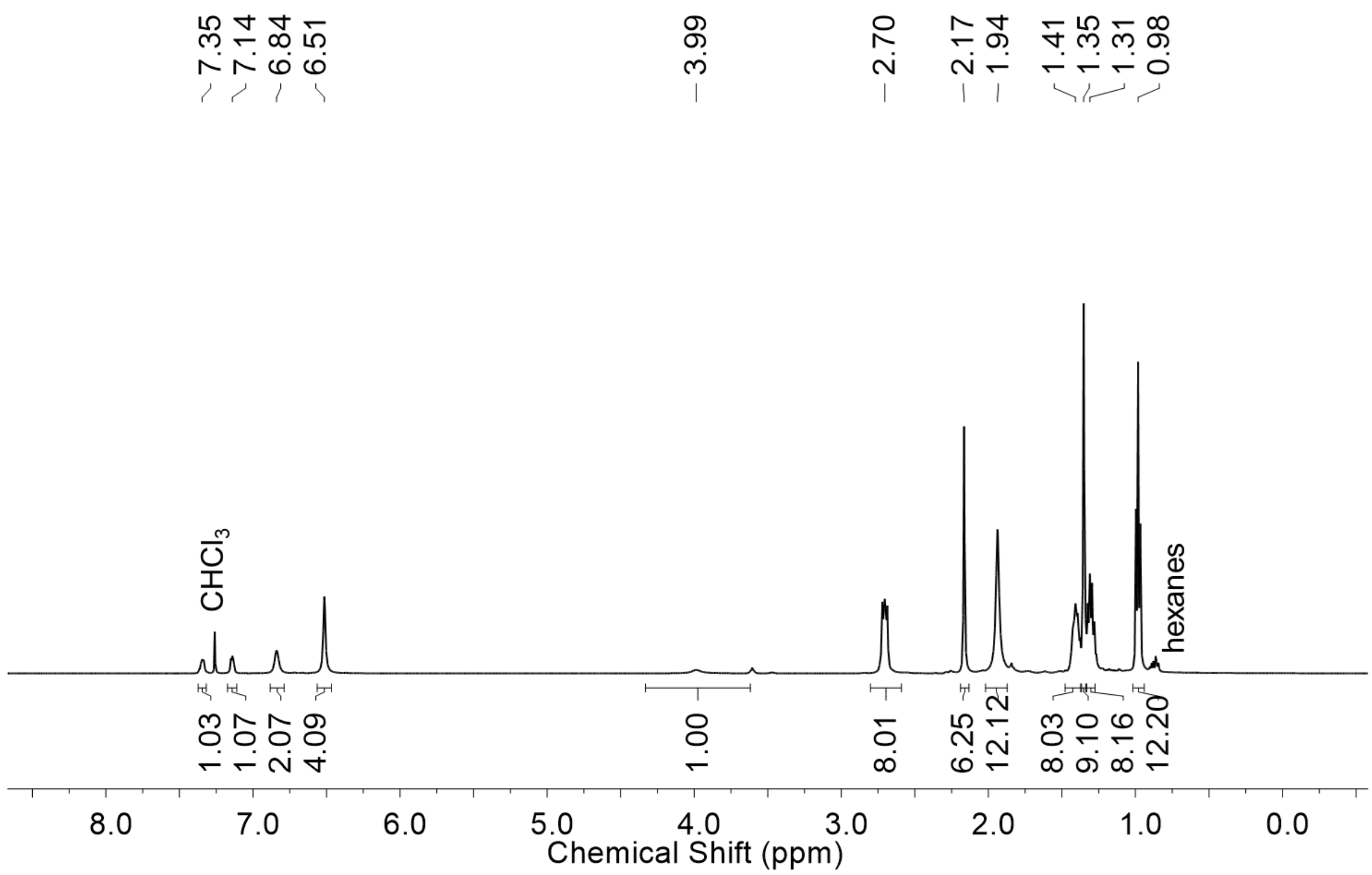

*Figure S 23. ^{1}H{^{11}B} NMR spectrum of (^{n}Bu$_4$N)**4** in CDCl$_3$.*

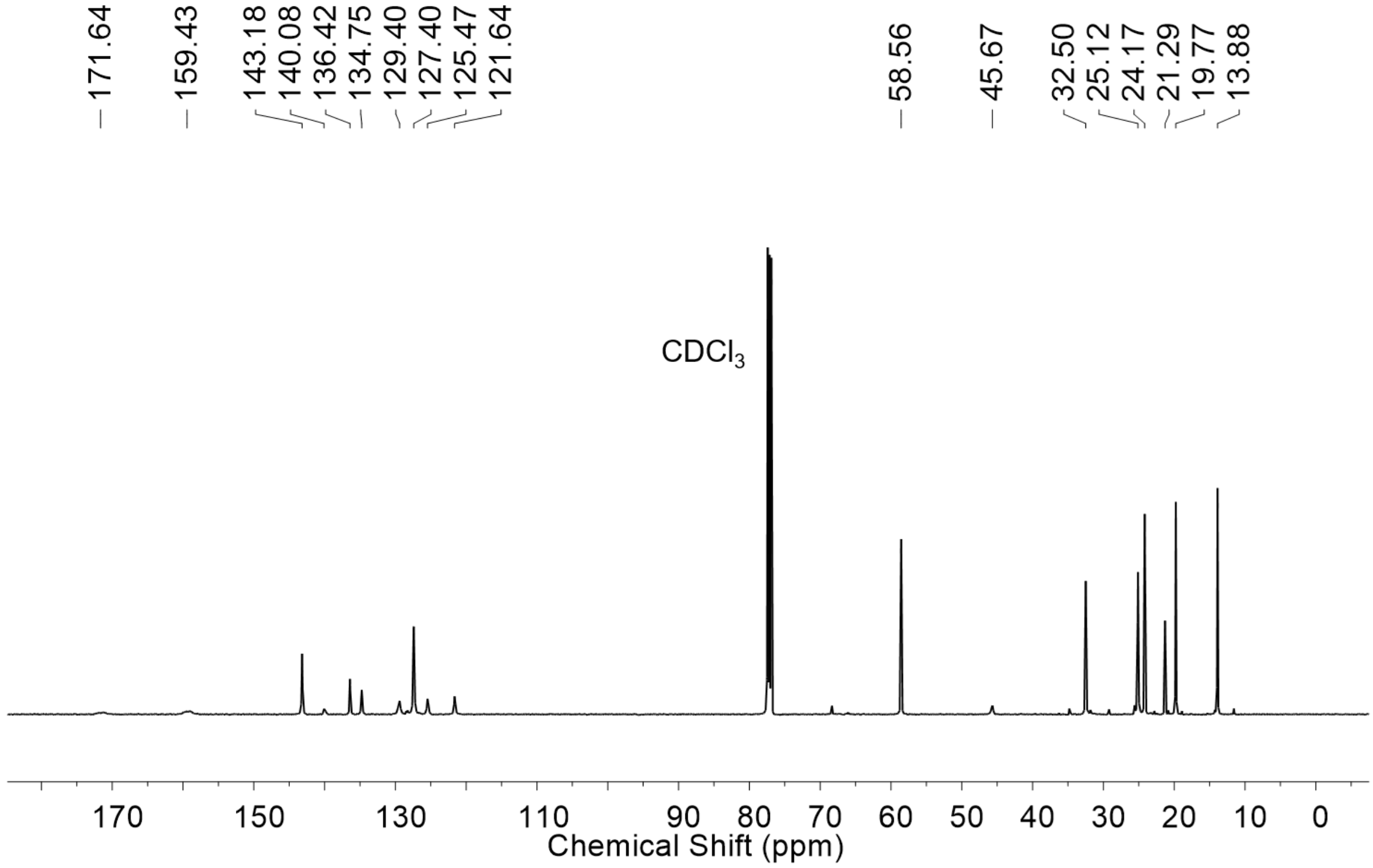

*Figure S 24. $^{13}C\{^{1}H\}$ NMR spectrum of ($^{n}Bu_4N$)**4** in $CDCl_3$.*

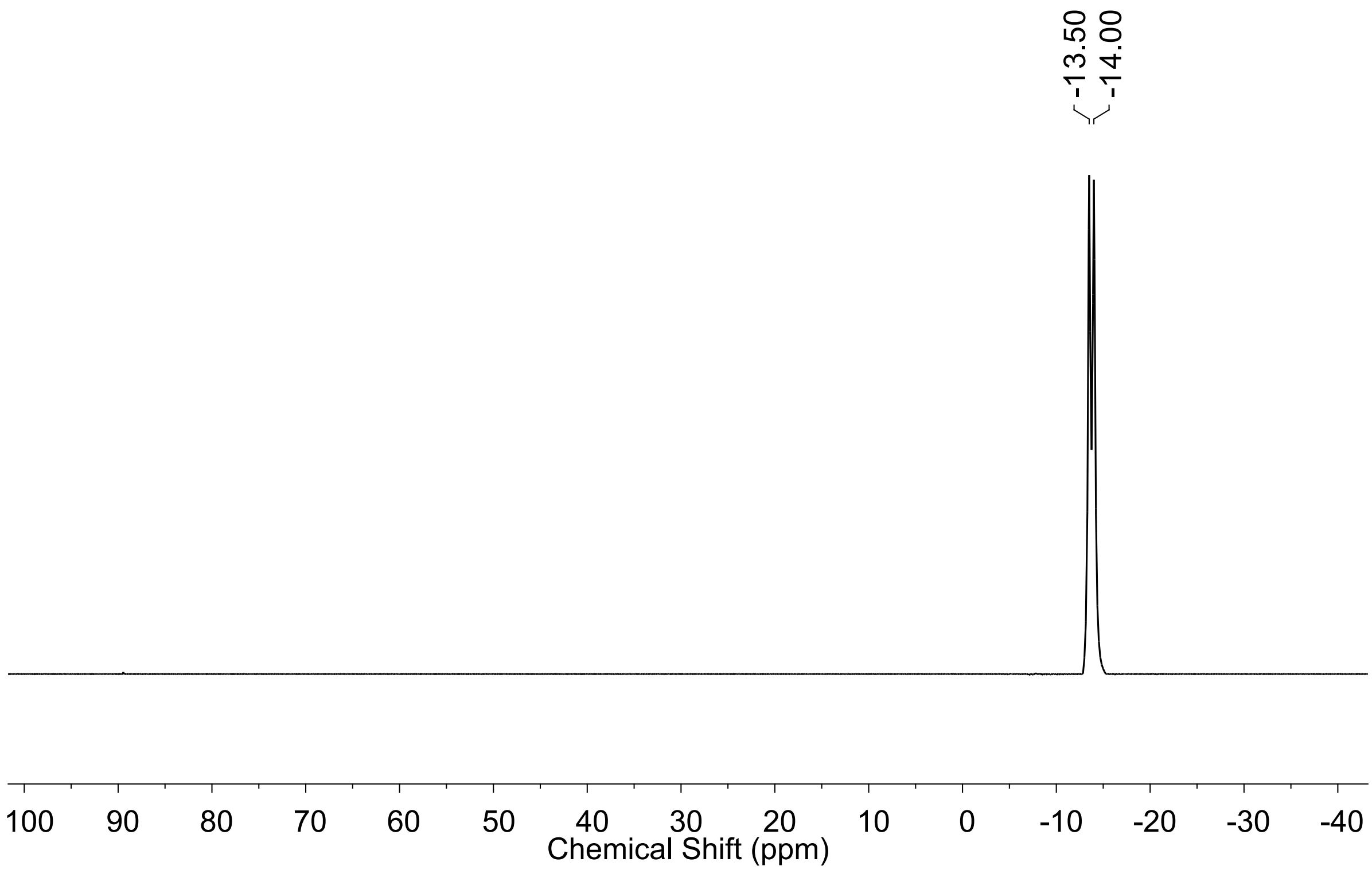

*Figure S 25. ^{11}B NMR spectrum of ($^{n}Bu_4N$)**4** in $CDCl_3$.*

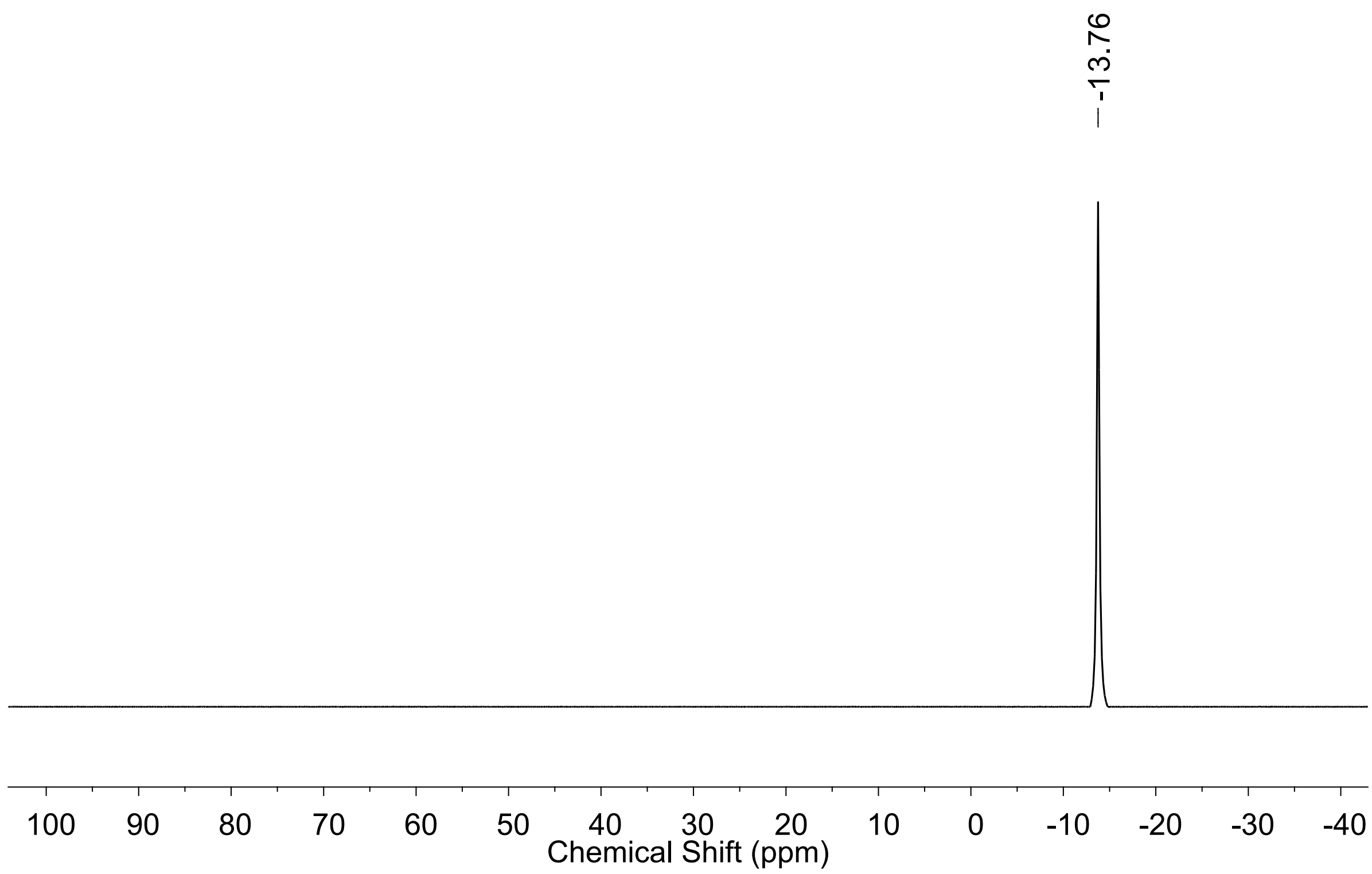

Figure S 26. $^{11}B\{^1H\}$ *NMR spectrum of (^{n}Bu$_4$N)**4** in $CDCl_3$.*

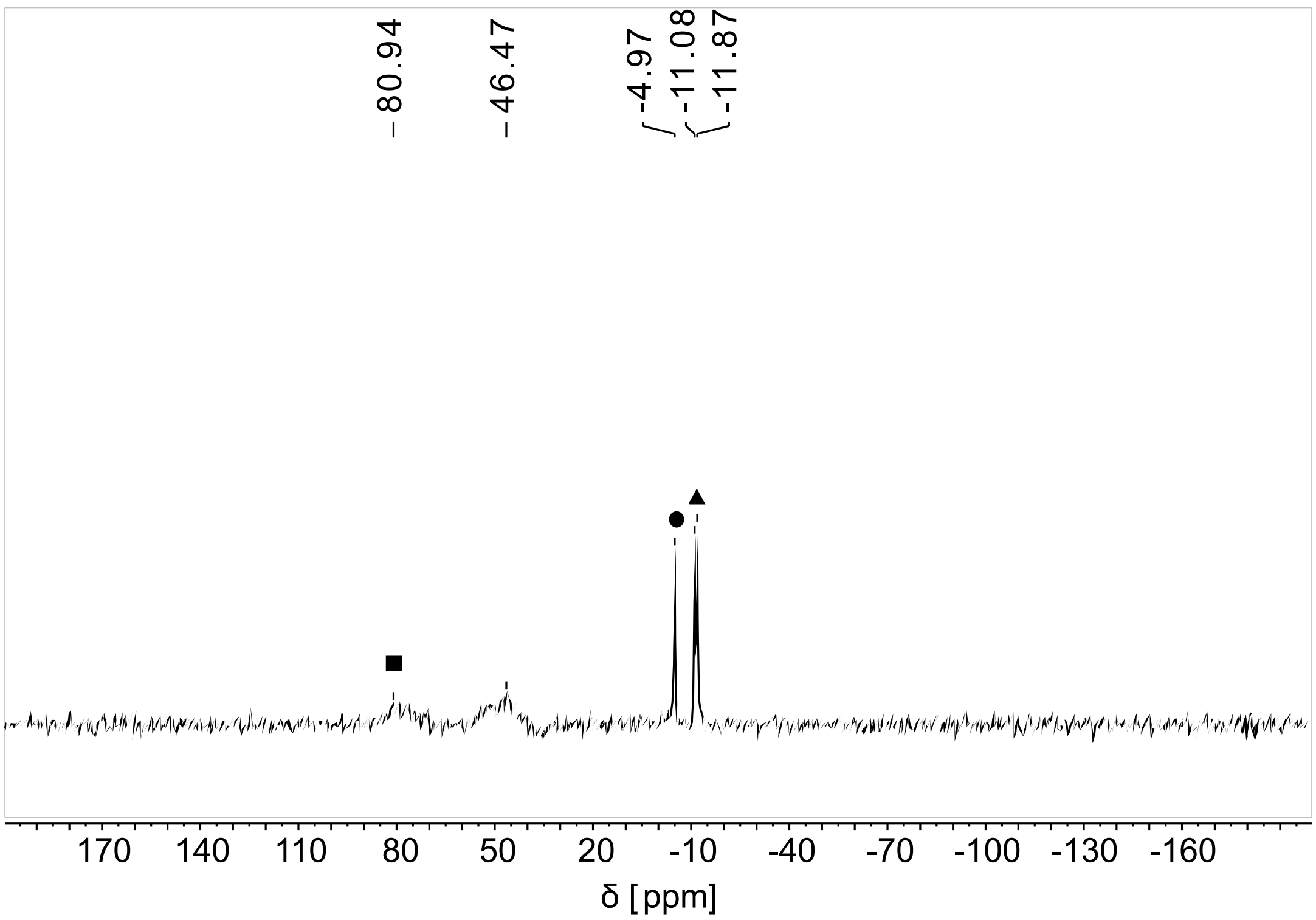

Figure S 27. ^{11}B*-BS NMR spectrum of K**4** in THF. Square: **3**, triangle: K**4**, circle: BPh_4^-.*

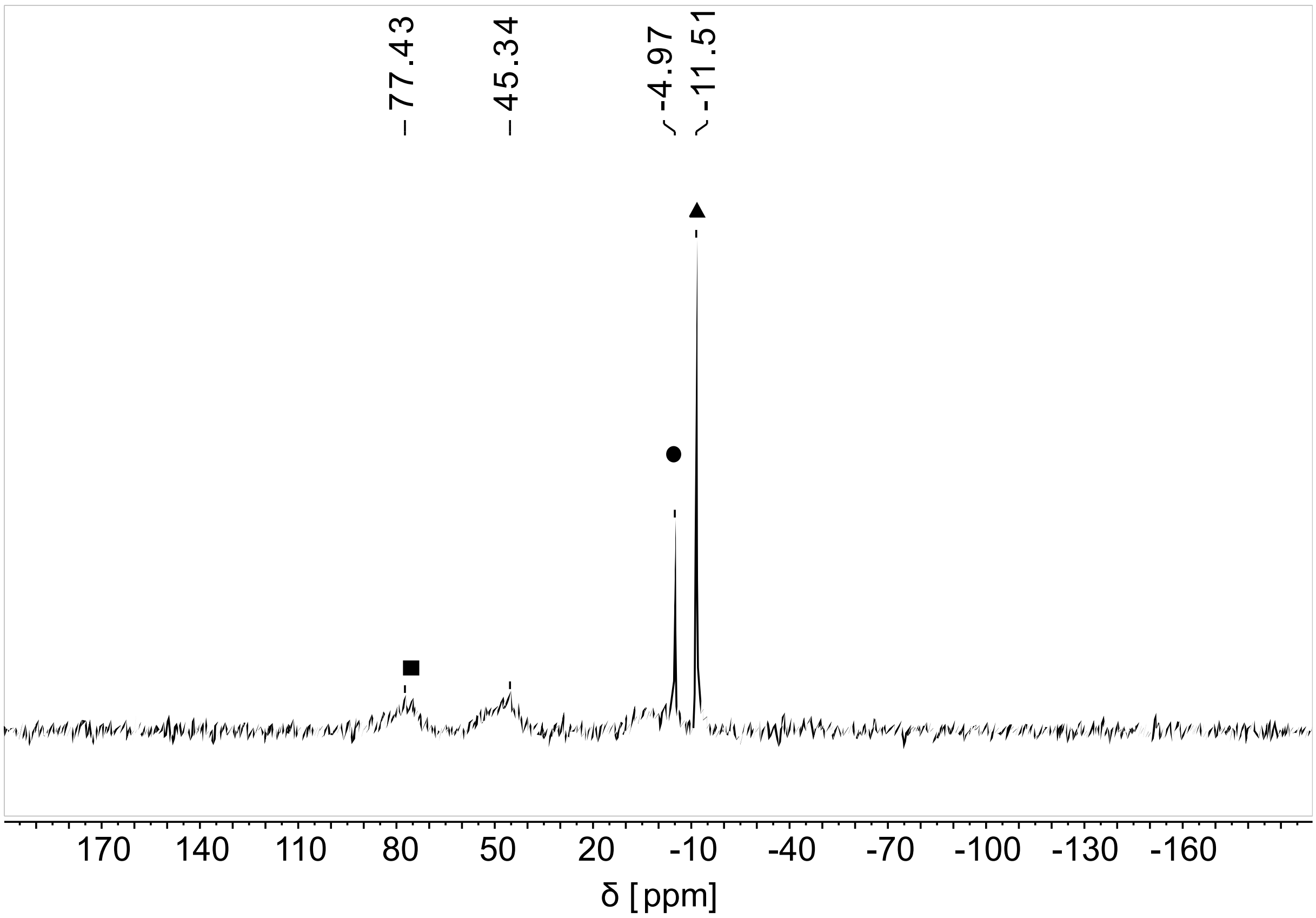

*Figure S 28. $^{11}B\{^{1}H\}$-BS NMR spectrum of K**4** in THF. Square: **3**, triangle: K**4**, and circle: BPh_4^{-}.*

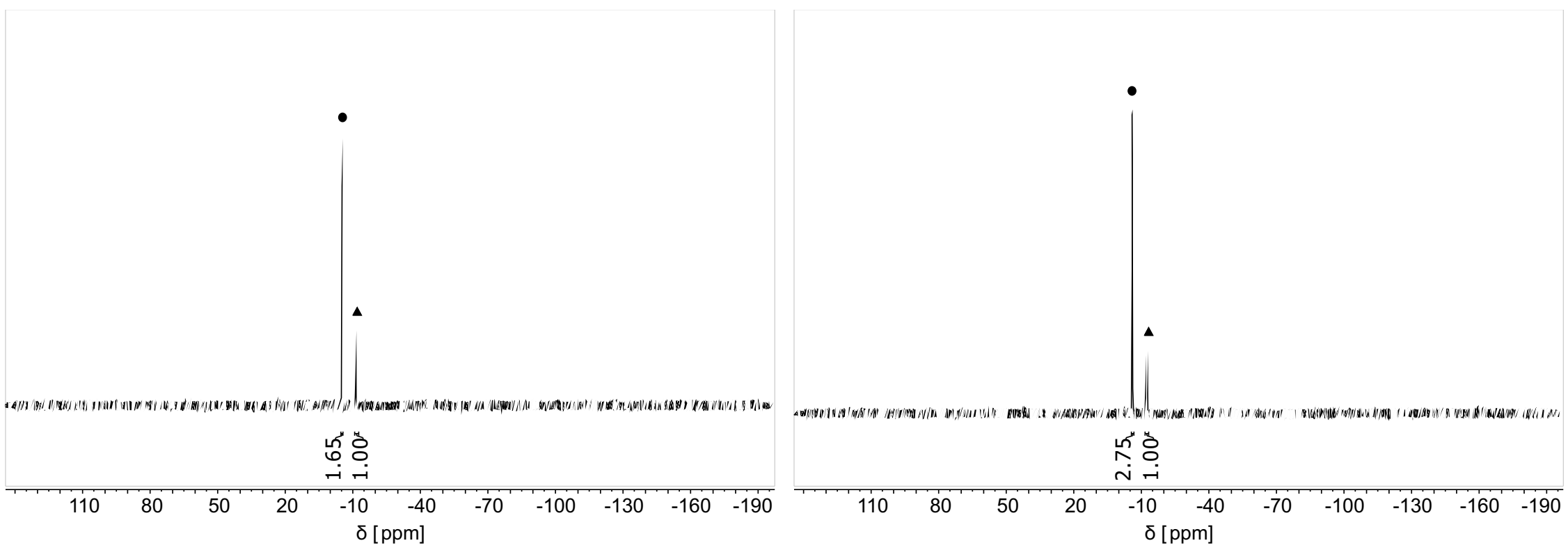

*Figure S 29: ^{11}B NMR spectrum of the reaction of **3**, 1 eq. [DBUH][BPh_4] and 2 eq. KC_8 after decantation of the THF solution without (left) and with (right) 9.1 mg of $^{n}Hex_4NBAr^{F}_4$ as an internal standard. Triangle: K**4**, circle: BPh_4^{-}.*

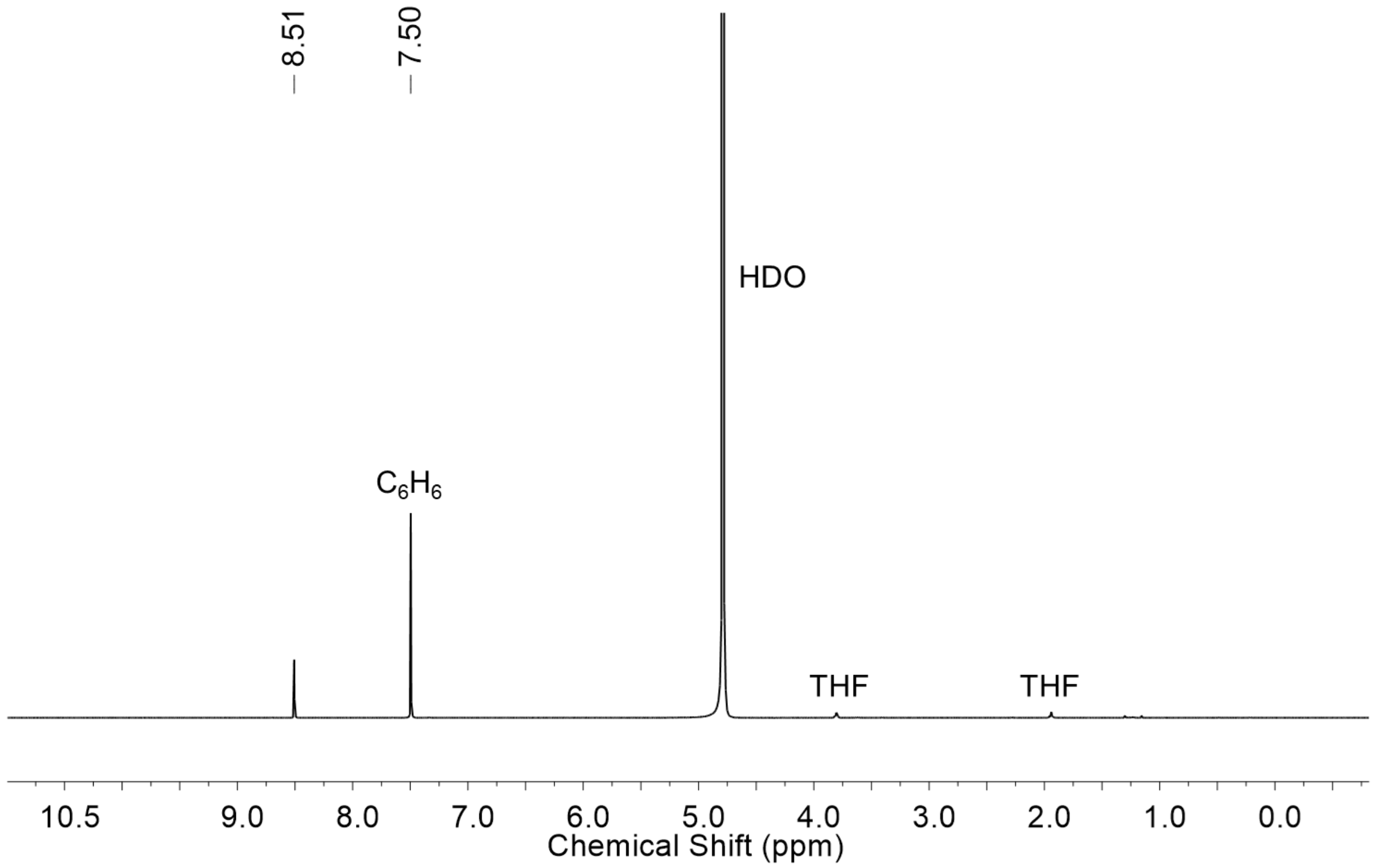

*Figure S 30. ^{1}H NMR spectrum of the D_2O solution after exposing [Li(THF)$_3$]**4** in THF to 1 atm of CO_2 at room temperature.*

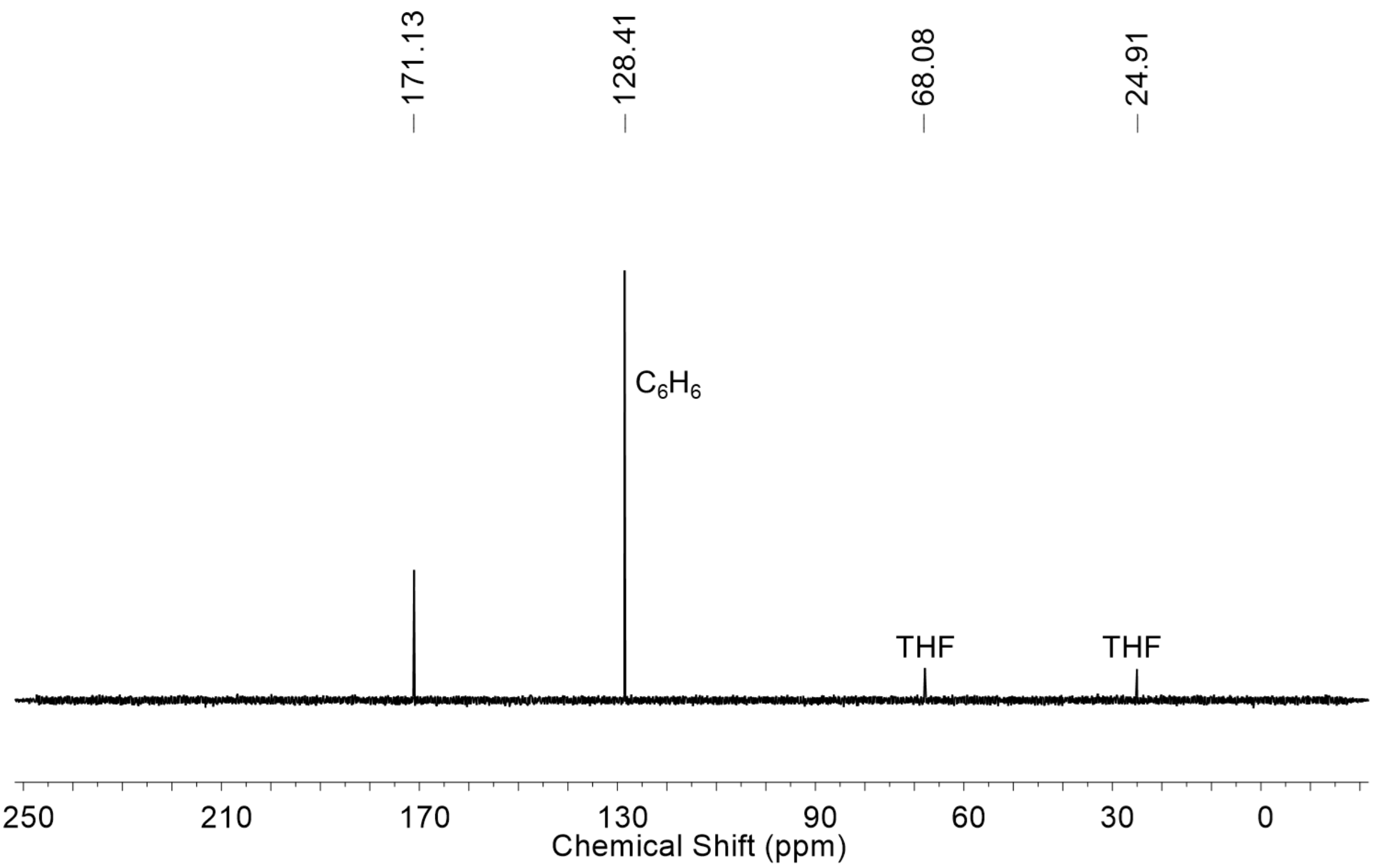

*Figure S 31. $^{13}C\{^{1}H\}$ NMR spectrum of the D_2O solution after exposing [Li(THF)$_3$]**4** in THF to 1 atm of CO_2 at room temperature.*

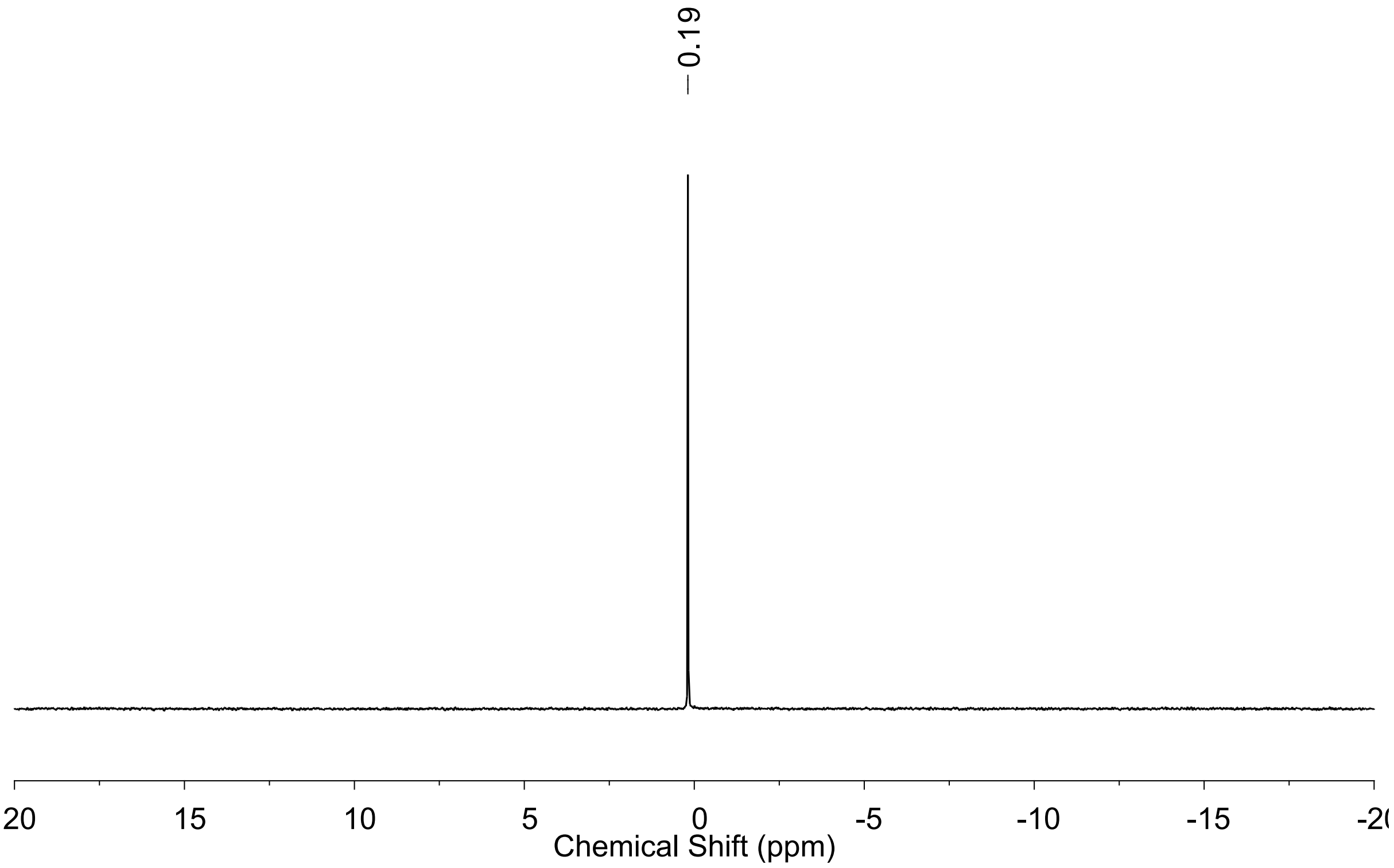

Figure S 32. $^{7}Li\{^{1}H\}$ *NMR spectrum of the* D_2O *solution after exposing [Li(THF)*$_3$*]**4** in THF to 1 atm of* CO_2 *at room temperature.*

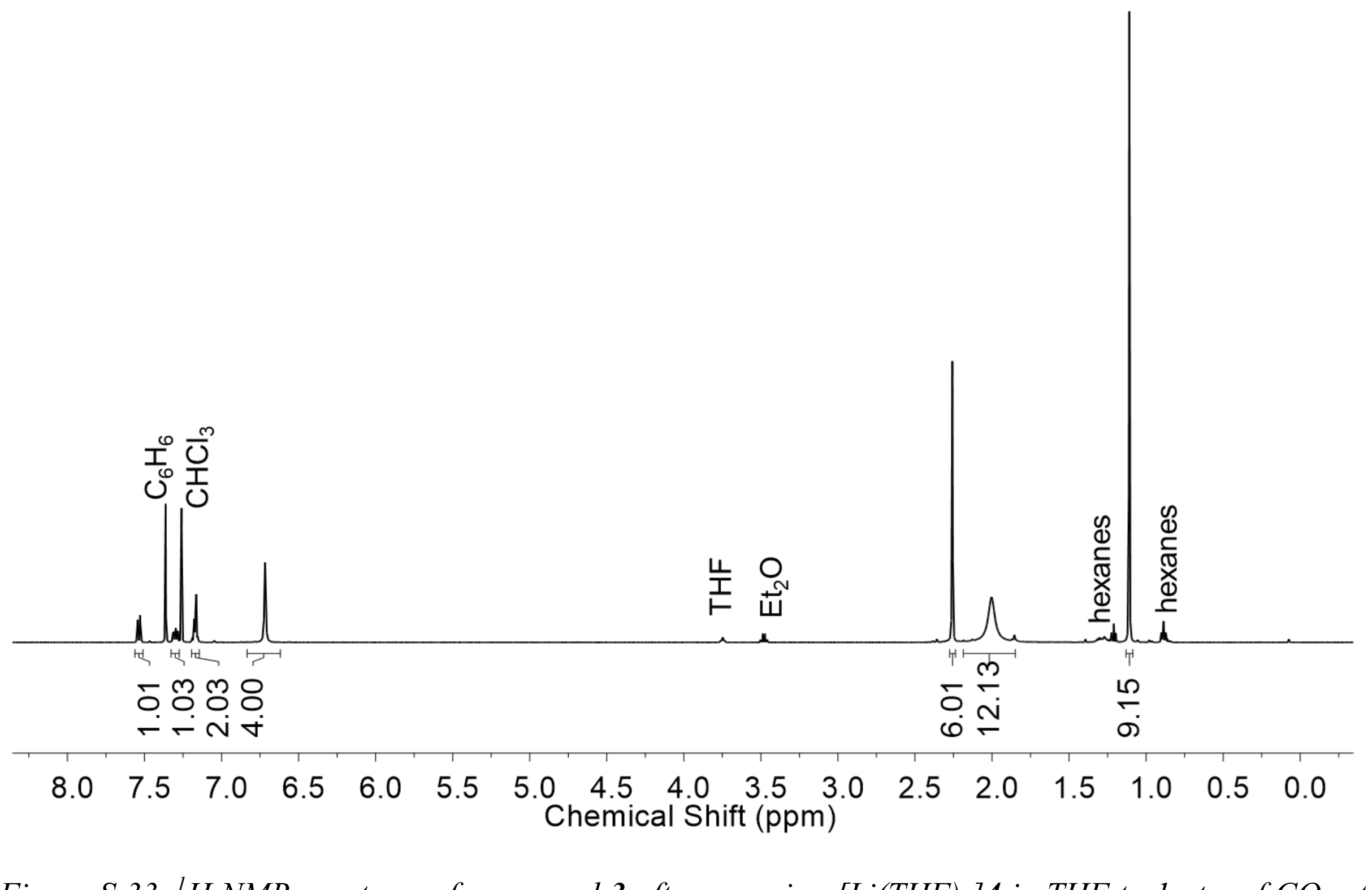

Figure S 33. ^{1}H *NMR spectrum of recovered **3** after exposing [Li(THF)*$_3$*]**4** in THF to 1 atm of* CO_2 *at room temperature.*

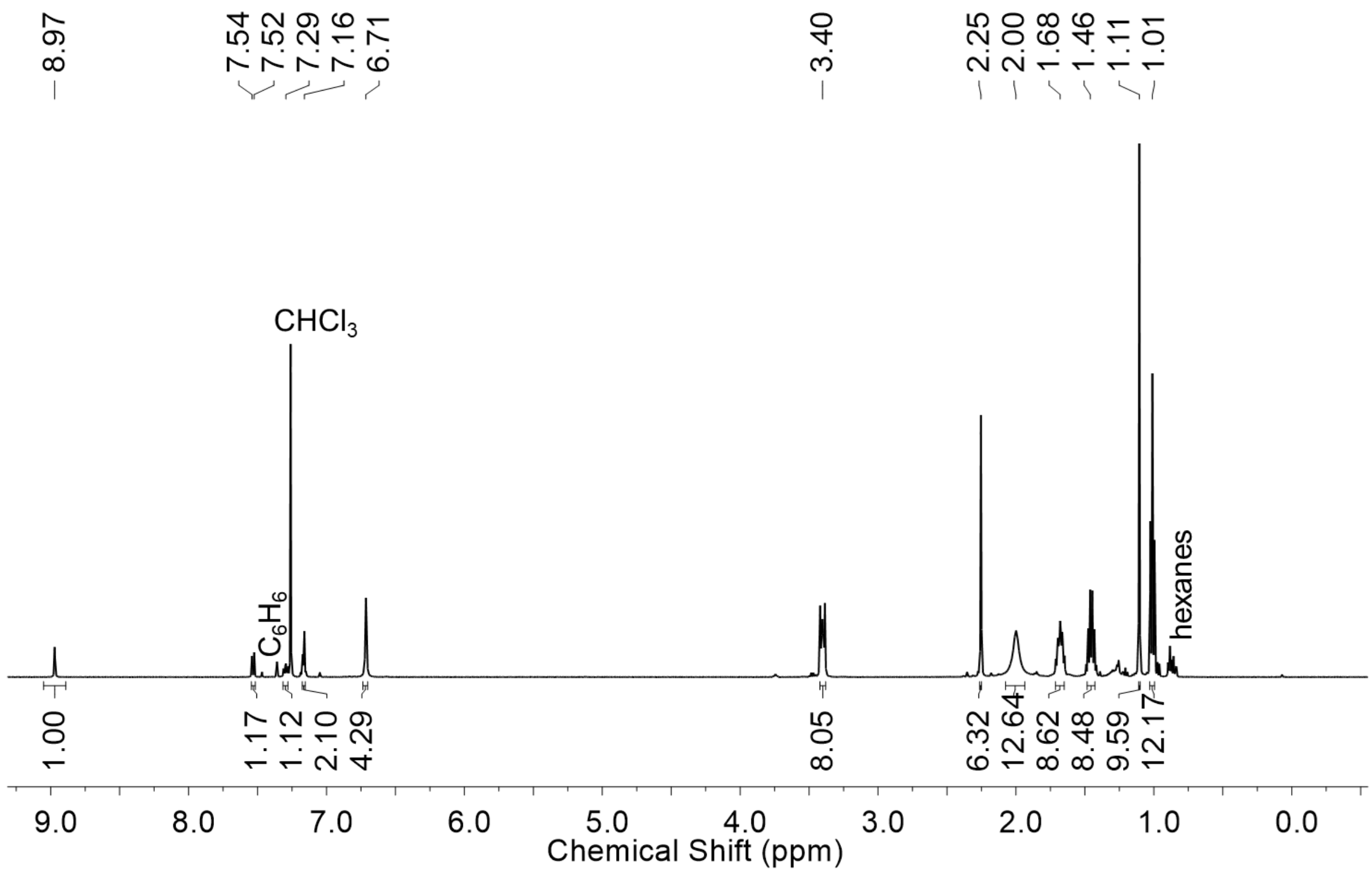

*Figure S 34. ^{1}H NMR spectrum of the reaction mixture of (^{n}Bu$_4$N)**4** and 1 atm CO_2 in $CDCl_3$ after 10 min. at room temperature.*

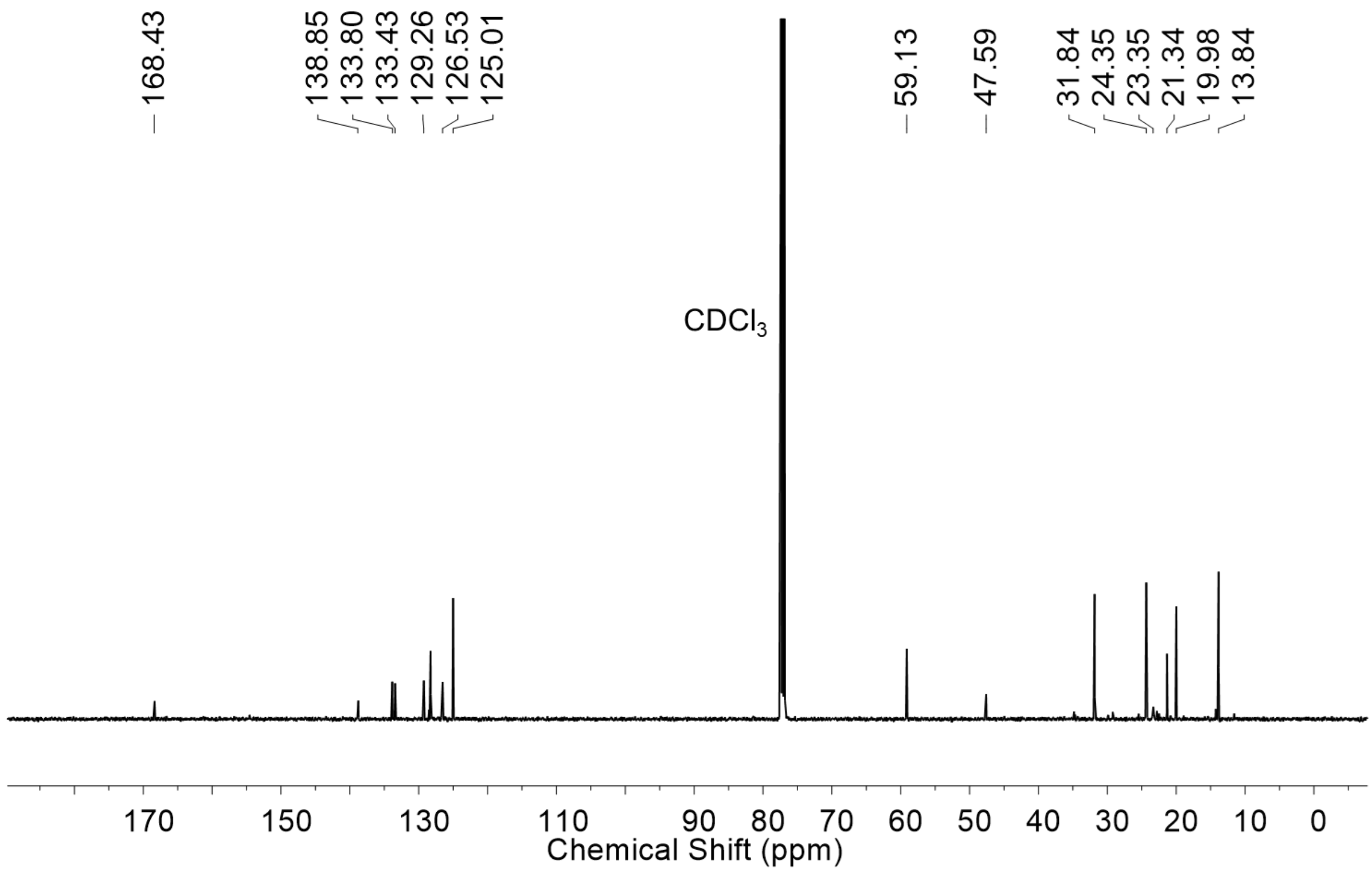

*Figure S 35. ^{13}C{^{1}H} NMR spectrum of the reaction mixture of (^{n}Bu$_4$N)**4** and 1 atm CO_2 in $CDCl_3$ at room temperature.*

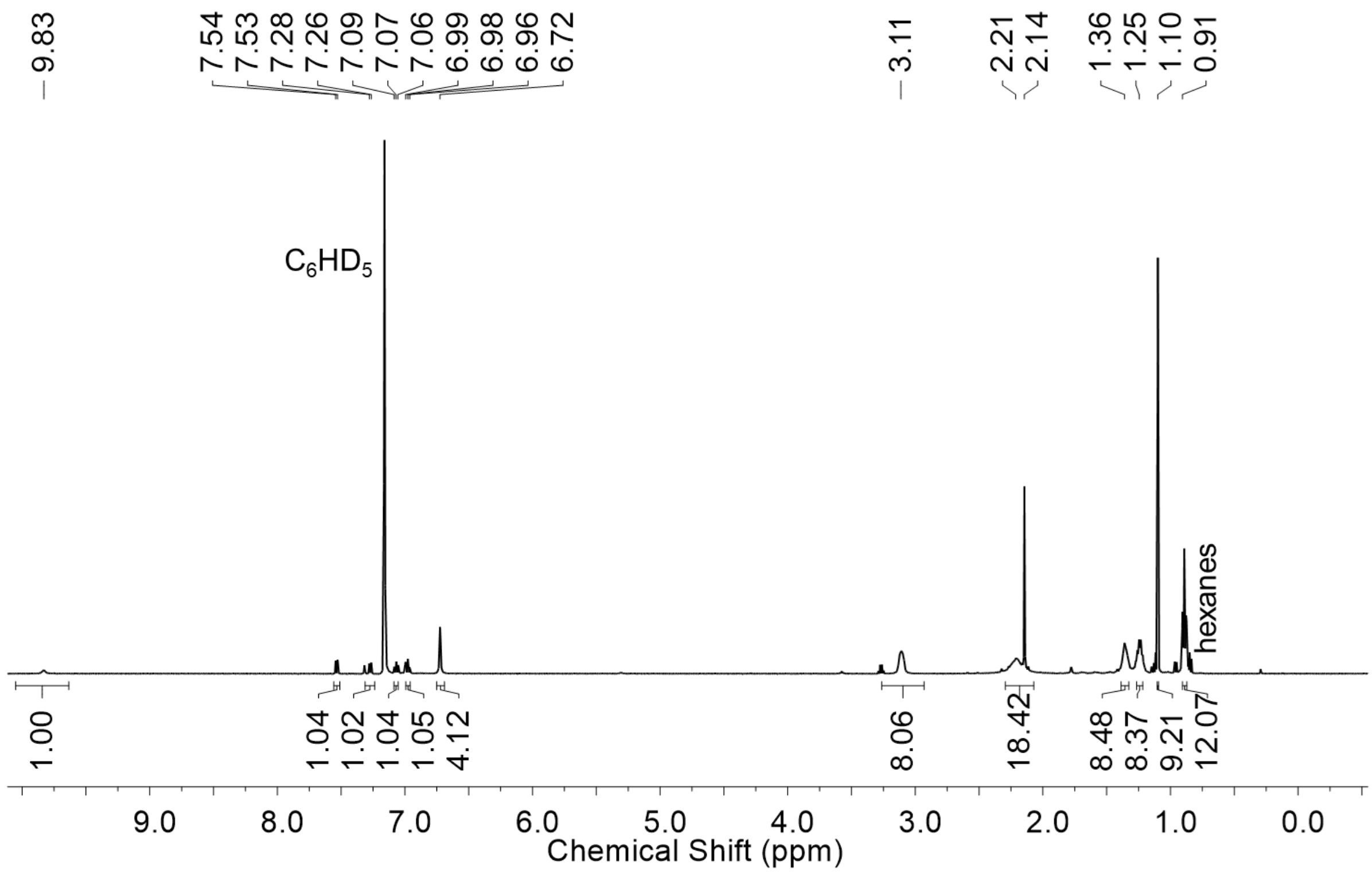

*Figure S 36. ^{1}H NMR spectrum of the reaction mixture of ($^{n}Bu_4N$)**4** and 1 atm CO_2 in C_6D_6 after 10 min. at room temperature.*

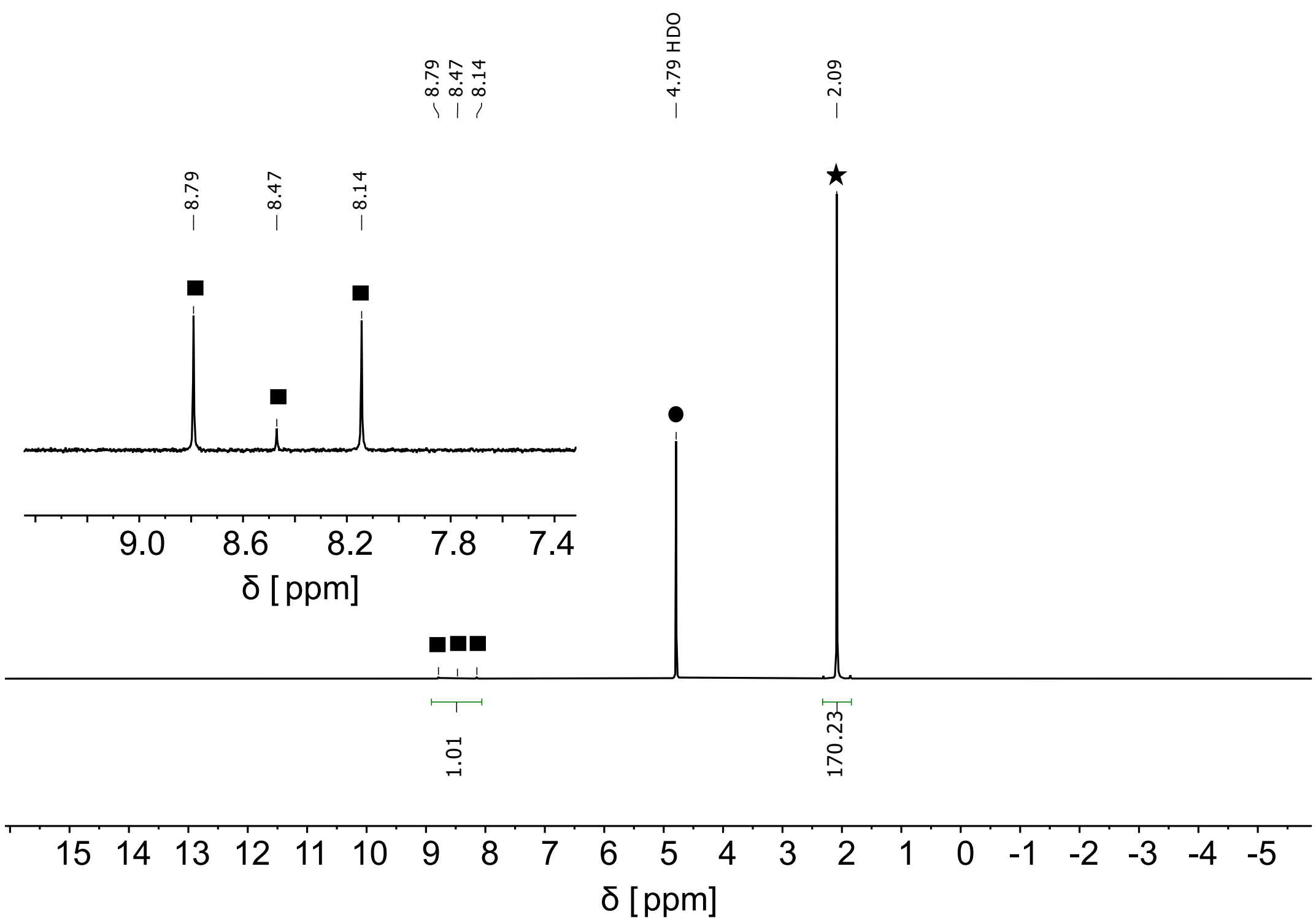

*Figure S 37. ^{1}H NMR spectrum after the reaction of **3**, 2 eq. KC_8 and 1 eq. [DBUH][BPh_4] under 1.2 atm of $^{13}CO_2$ in THF-d_8. The NMR spectrum was recorded in D_2O after workup; MeCN as internal standard, inset: formate signal. Star: MeCN, circle: D_2O, square: potassium formate.*

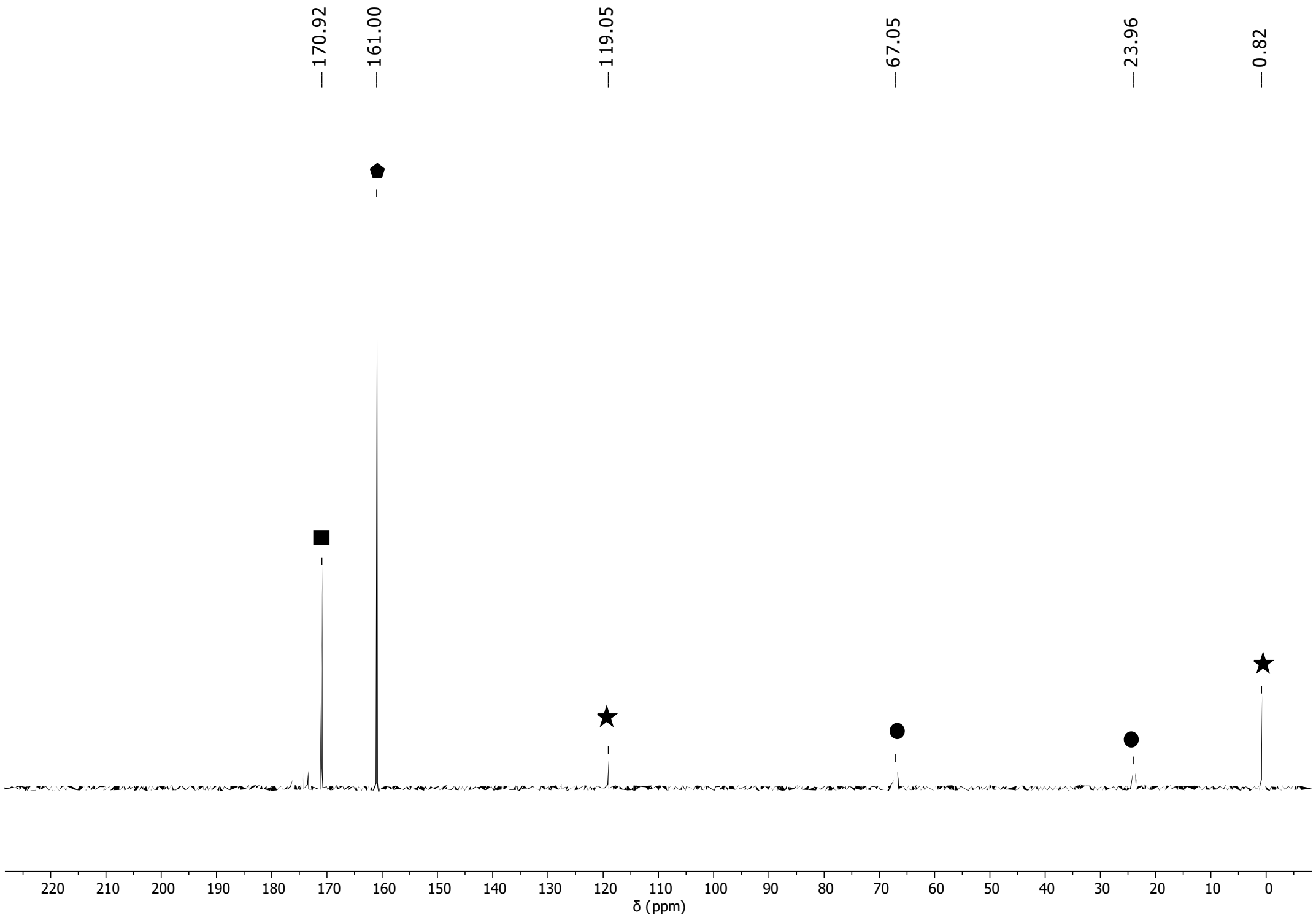

*Figure S 38. $^{13}C\{^{1}H\}$ NMR spectrum of the reaction of **3**, 2 eq. KC_8 and 1 eq. [DBUH][BPh_4] under 1.2 atm of $^{13}CO_2$ in THF-d_8. The NMR spectrum is recorded in D_2O after the workup; MeCN as internal standard. Star: MeCN, circle: THF-d_8, square: potassium carbonate, pentagon: potassium formate.*

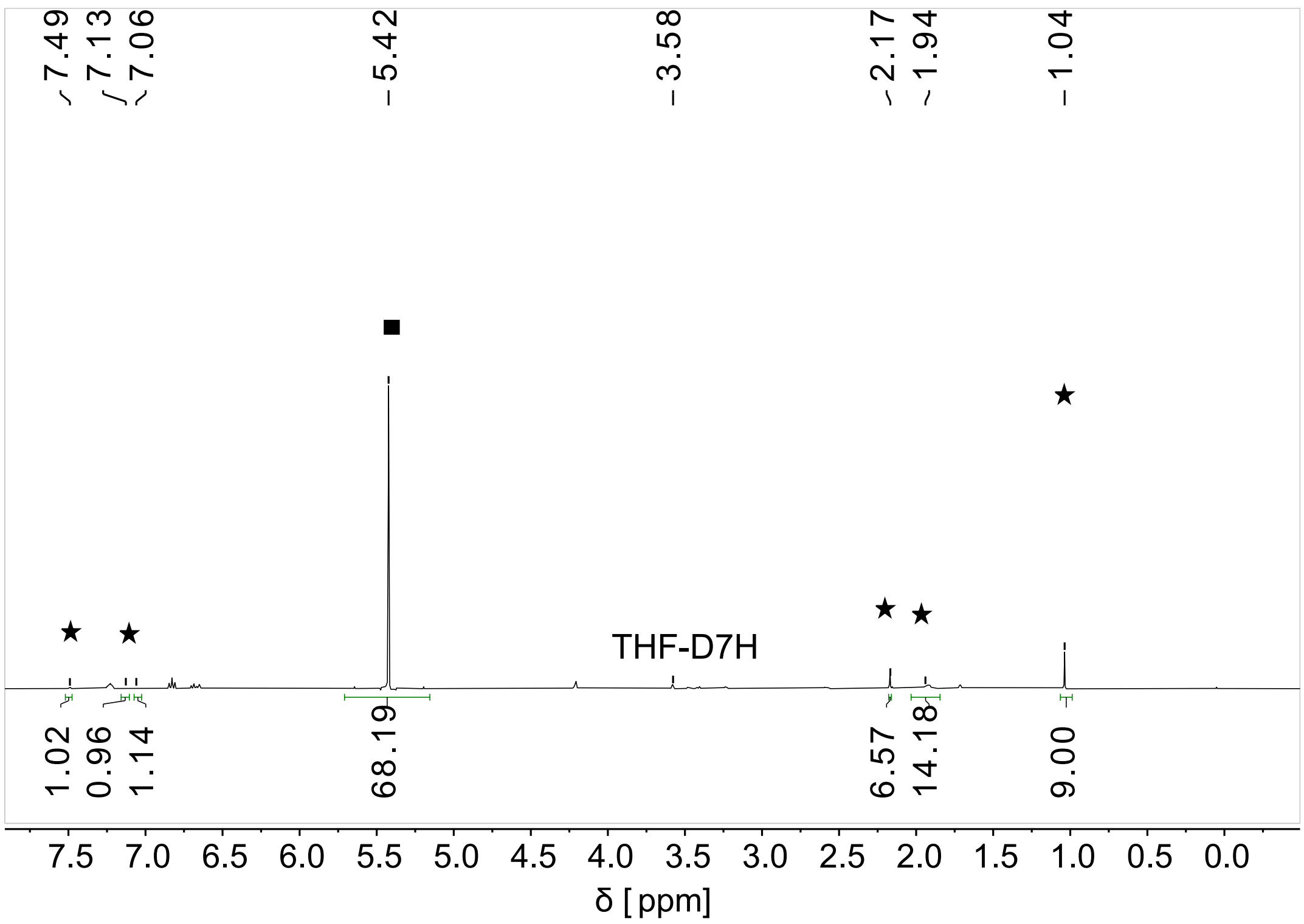

*Figure S 39. ^{1}H NMR spectrum of the reaction of **3**, 2 eq. KC_8 and 1 eq. [DBUH][BPh_4] under 1.2 atm of $^{13}CO_2$ in THF-d_8. The NMR spectrum is recorded in THF-d_8 after the workup, CH_2Cl_2 as internal standard. Square: CH_2Cl_2, star: **3**.*

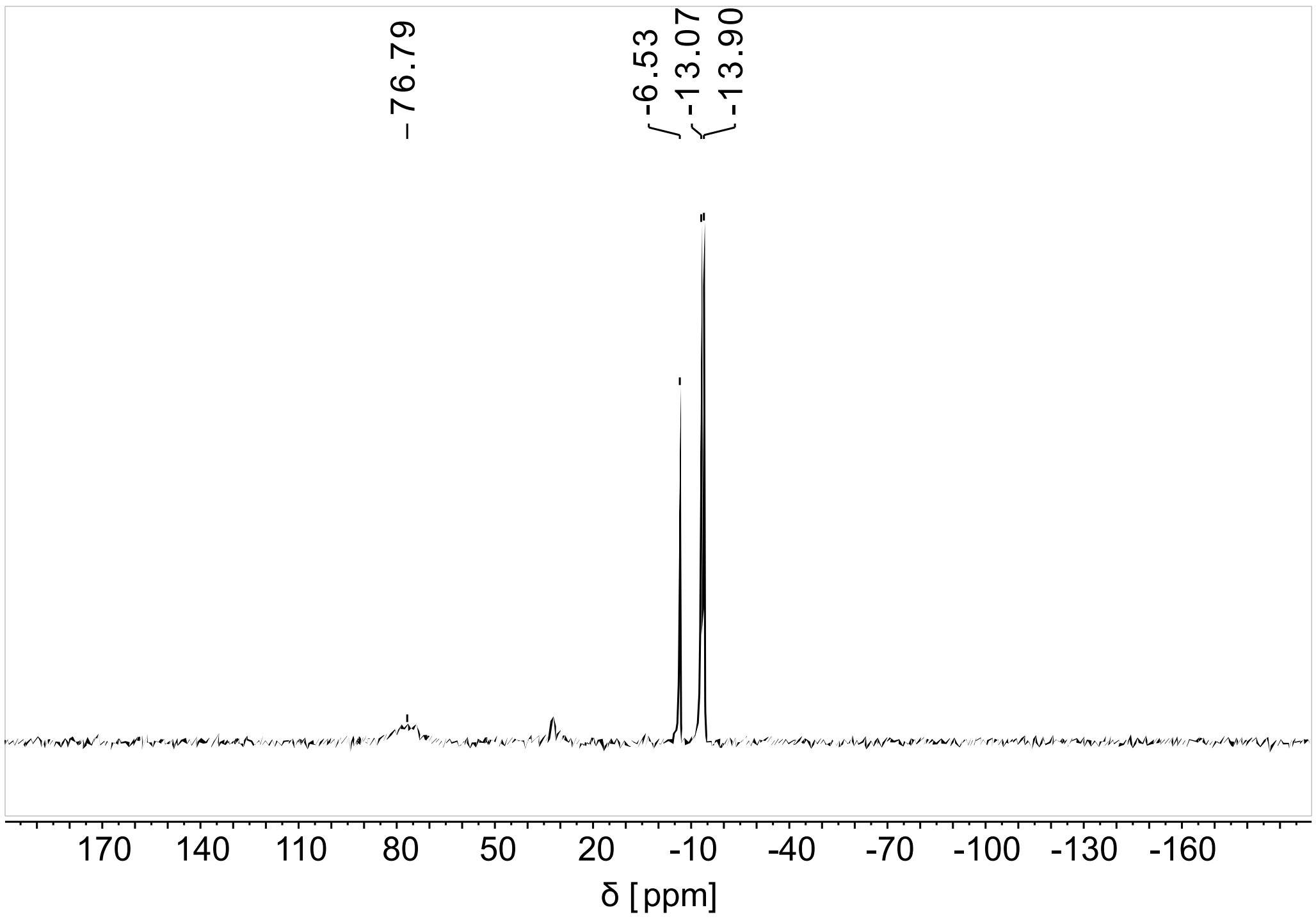

Figure S 40. ^{11}B-BS NMR spectrum of the reaction mixture of ***3****, 2 eq. KC_8, 1 eq. [DBUH][BPh_4] under 1.2 atm of $^{13}CO_2$ in THF-d_8 at room temperature after 10 min prior to the workup. Square:* ***3****, circle: BPh_4^-.*

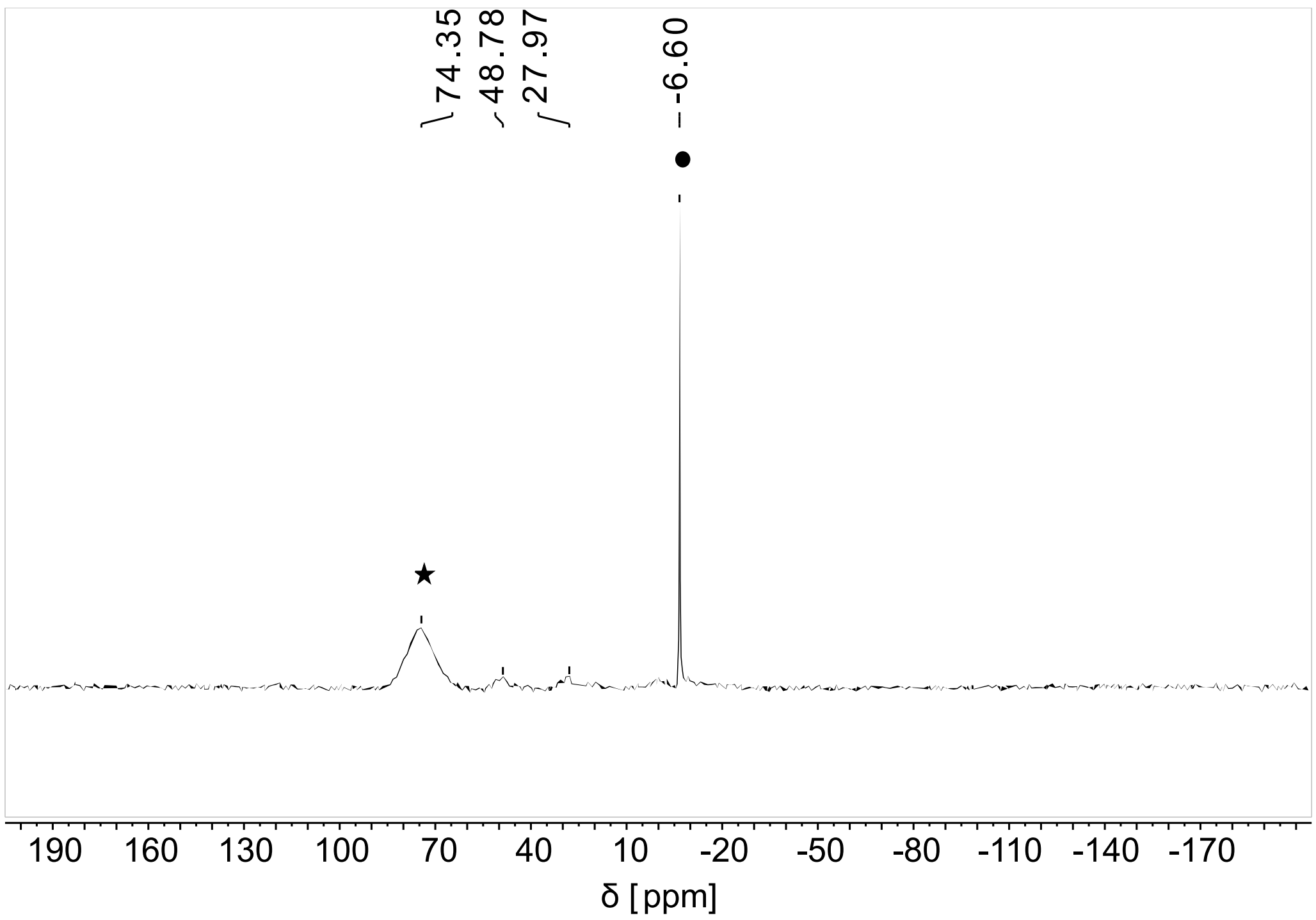

Figure S 41. ^{11}B NMR spectrum of the reaction mixture of ***3****, 2 eq. KC_8, 1 eq. [DBUH][BPh_4] under 1.2 atm of $^{13}CO_2$ in THF-d_8, after workup. Star:* ***3****, circle: BPh_4^-.*

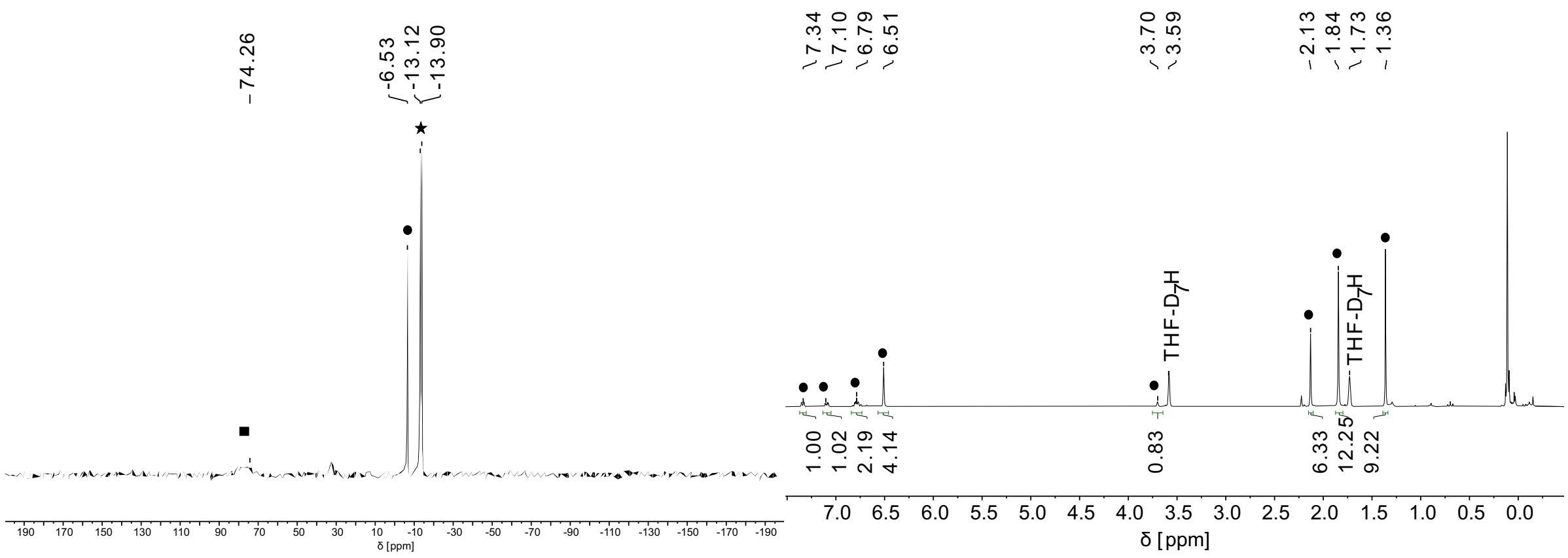

*Figure S 42: Left: ^{11}B-BS NMR spectrum of the reaction of a mixture of Na**4** and 2.8 eq. [DBUH][BPh$_4$] in THF-d$_8$ after 23 h. Square: **3**, circle: BPh$_4^-$, star: Na**4**. Right: ^{1}H{^{11}B} NMR spectrum of Na**4** in THF-d$_8$ after the reaction of **3** and 1 eq. Na(Et$_3$BH) and the removal of all volatiles under vacuum. Circle: Na**4**.*

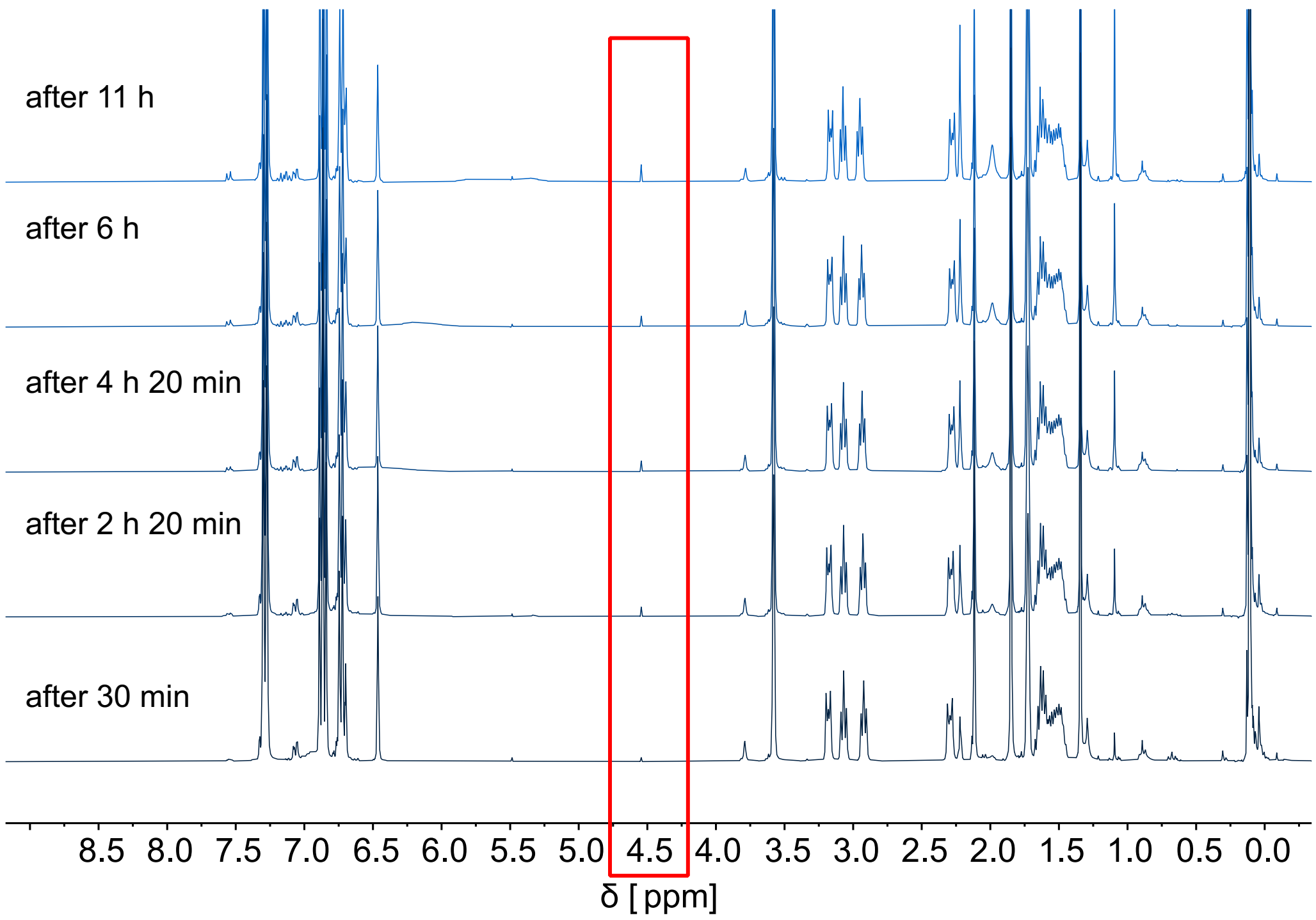

*Figure S 43: ^{1}H{^{11}B} NMR spectra in THF-d$_8$ monitoring the reaction of Na**4** with 2.8 eq. [DBUH][BPh$_4$] over a period of 11 h. The appearance of the H$_2$ signal at 4.55 ppm is highlighted in red.*

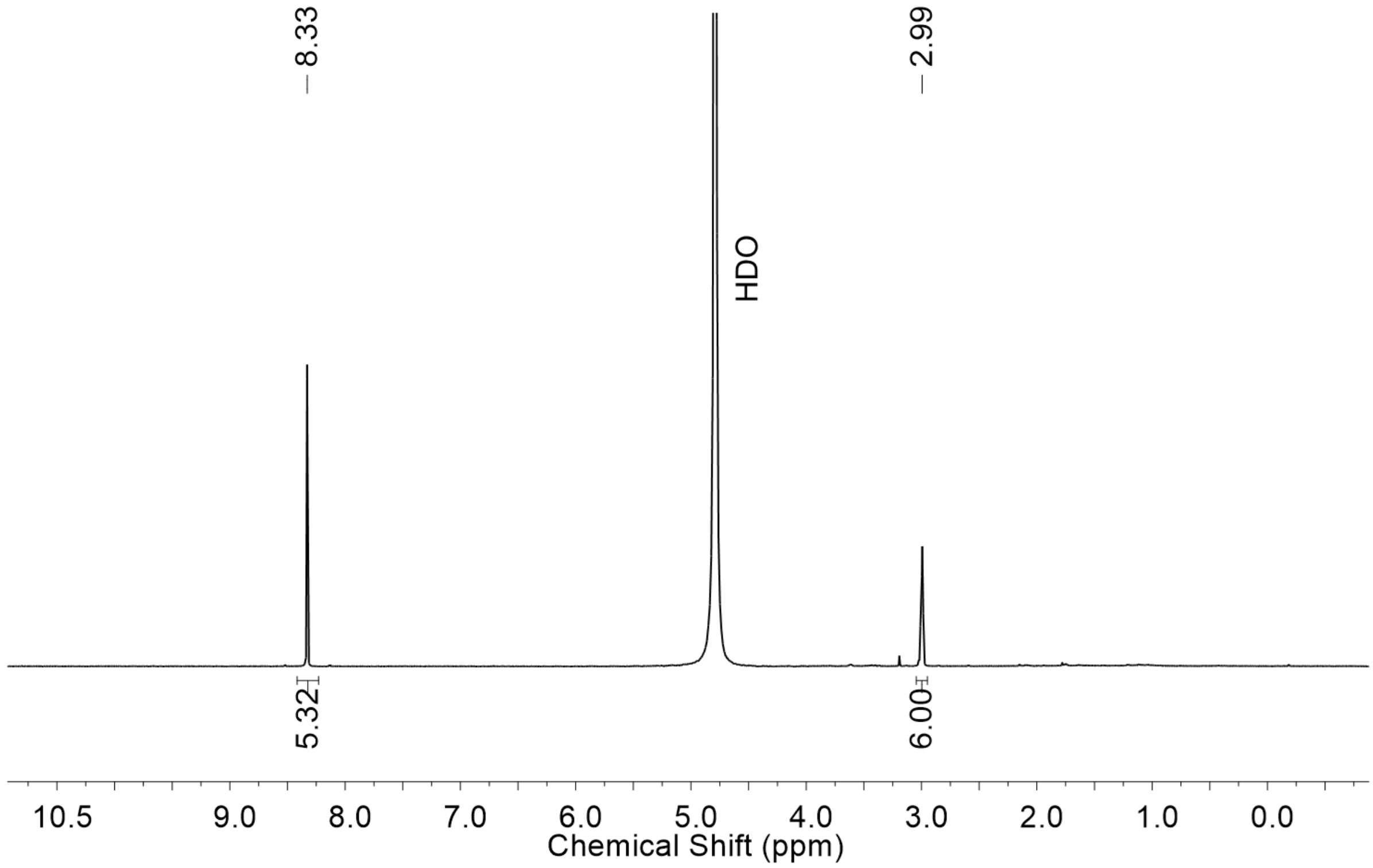

*Figure S 44. ^{1}H NMR spectrum of potassium formate with methylsulfonylmethane as internal standard in D_2O; aliquot of the aqueous phase after the catalytic reaction of **3**, KH and CO_2.*

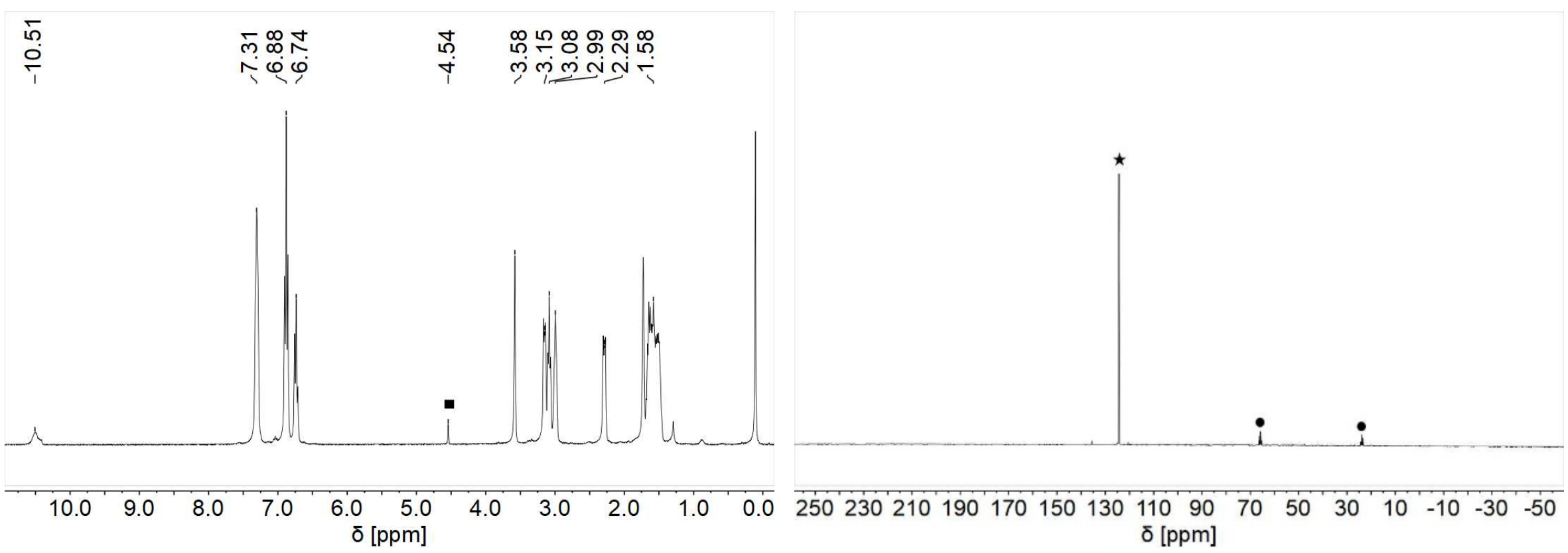

Figure S 45: ^{1}H NMR data (left) and $^{13}C\{^{1}H\}$ NMR data of the reaction mixture of [DBUH][BPh_4], 2 eq. KC_8, and $^{13}CO_2$ in THF-d_8 after 3 h. Square: H_2, star: $^{13}CO_2$, circle: THF-d_8.

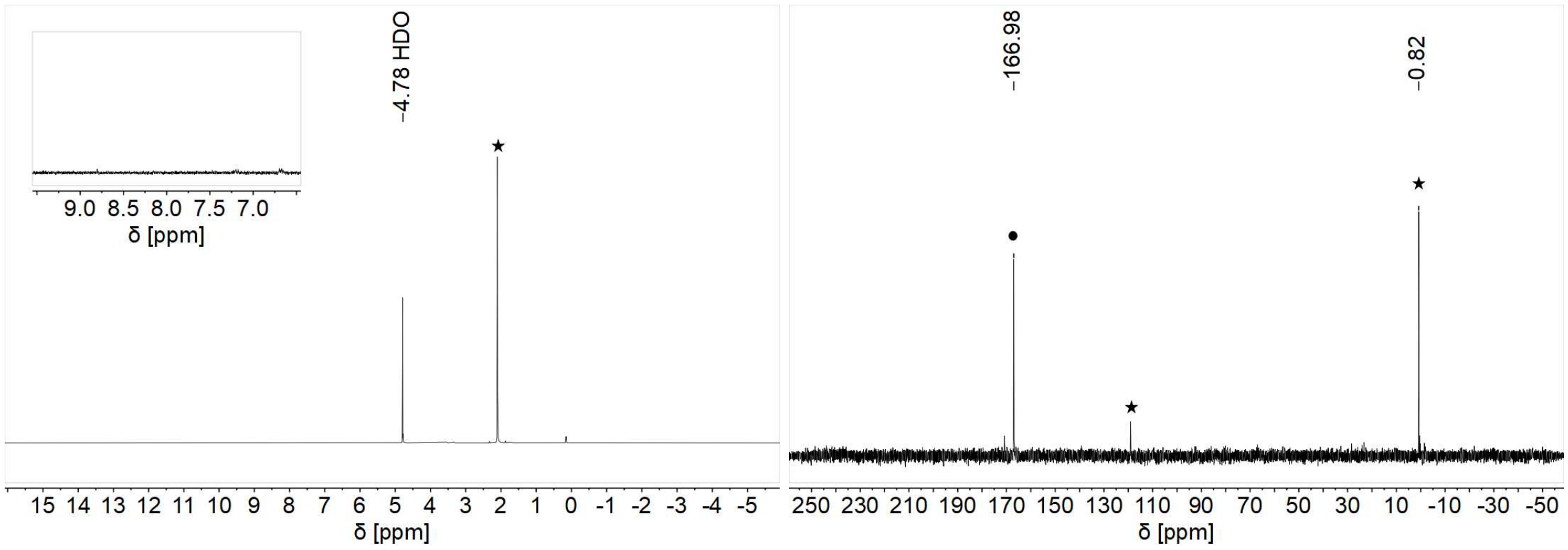

Figure S 46: ^{1}H NMR data (left) and ^{13}C{^{1}H} NMR data (right) of the reaction mixture of [DBUH][BPh$_4$] and 2 eq. KC_8, and $^{13}CO_2$ in THF after the removal of the solvent, extraction in D_2O and the addition of MeCN as internal standard. Star: MeCN, circle: HCO_3^-. Inset: region of the formate signal.

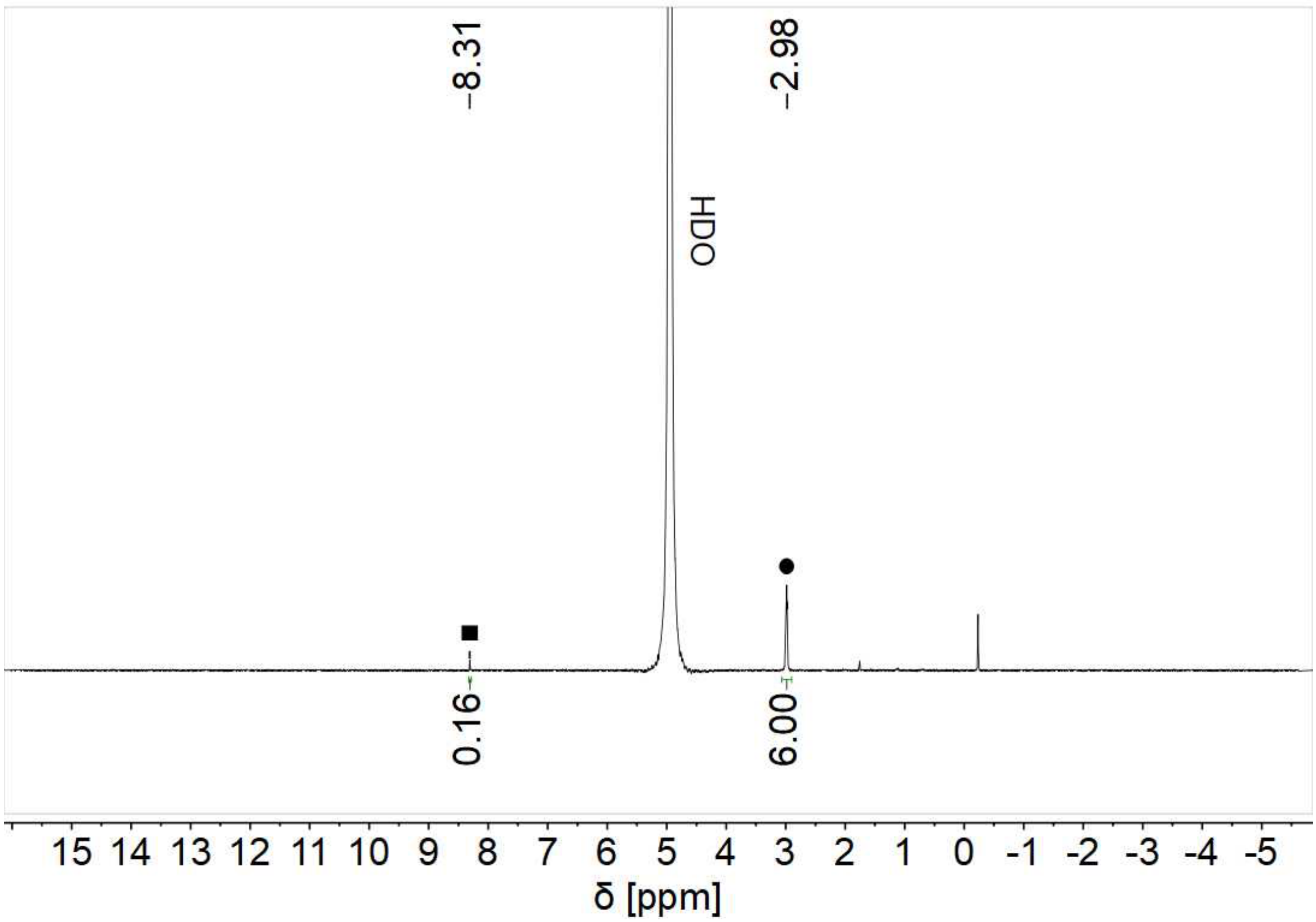

Figure S 47: ^{1}H NMR spectrum of potassium formate with methylsulfonylmethane as internal standard, recorded from the blank experiment of KH and CO_2, after removal of all volatiles and subsequent dissolution in D_2O. Circle: methylsulfonylmethane, square: potassium formate.

IR data

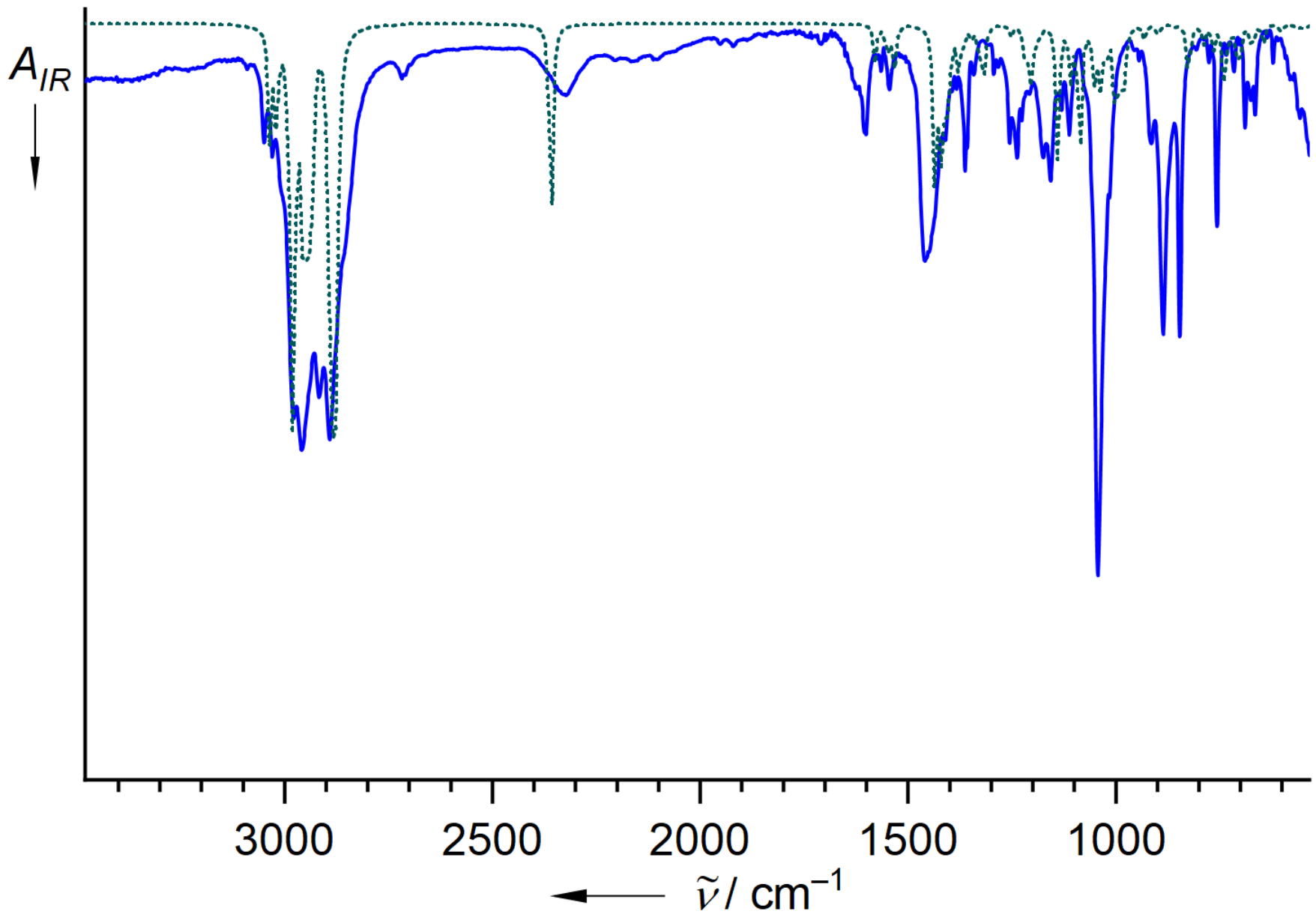

Figure S 48. Computed (doted trace, M06-2X/cc-pVTZ, scaled by 0.946) IR spectrum of ***4***$^{-}$ *and measured IR spectrum (solid trace, THF drop-cast film, KCl plate) of [Li(THF)$_3$]**4**.*

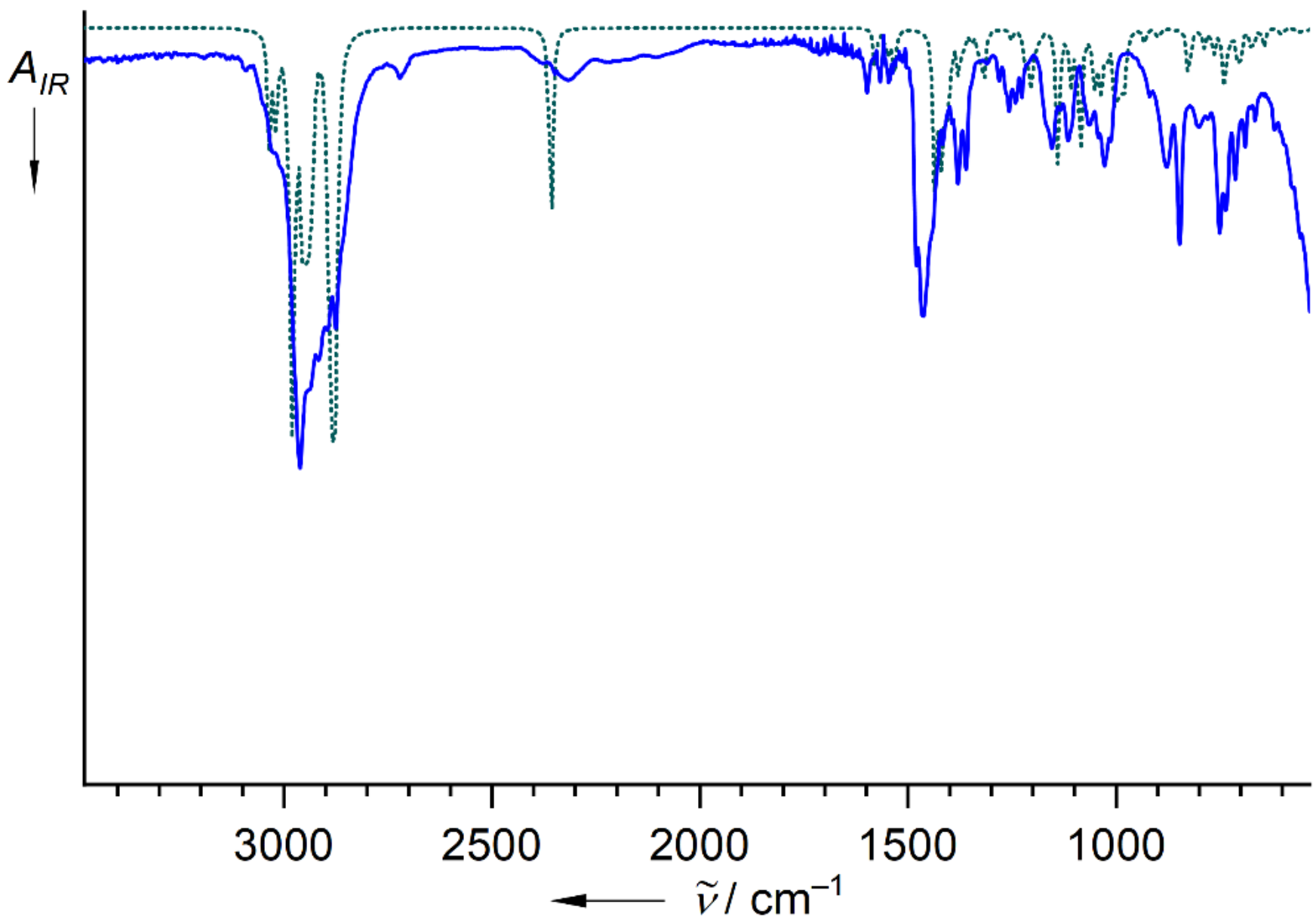

Figure S 49. Computed (doted trace, M06-2X/cc-pVTZ, scaled by 0.946) IR spectrum of ***4***$^{-}$ *and measured IR spectrum (solid trace, CH_2Cl_2 drop-cast film, KCl plate) of (^{n}Bu$_4$N)**4**.*

UV/Vis data

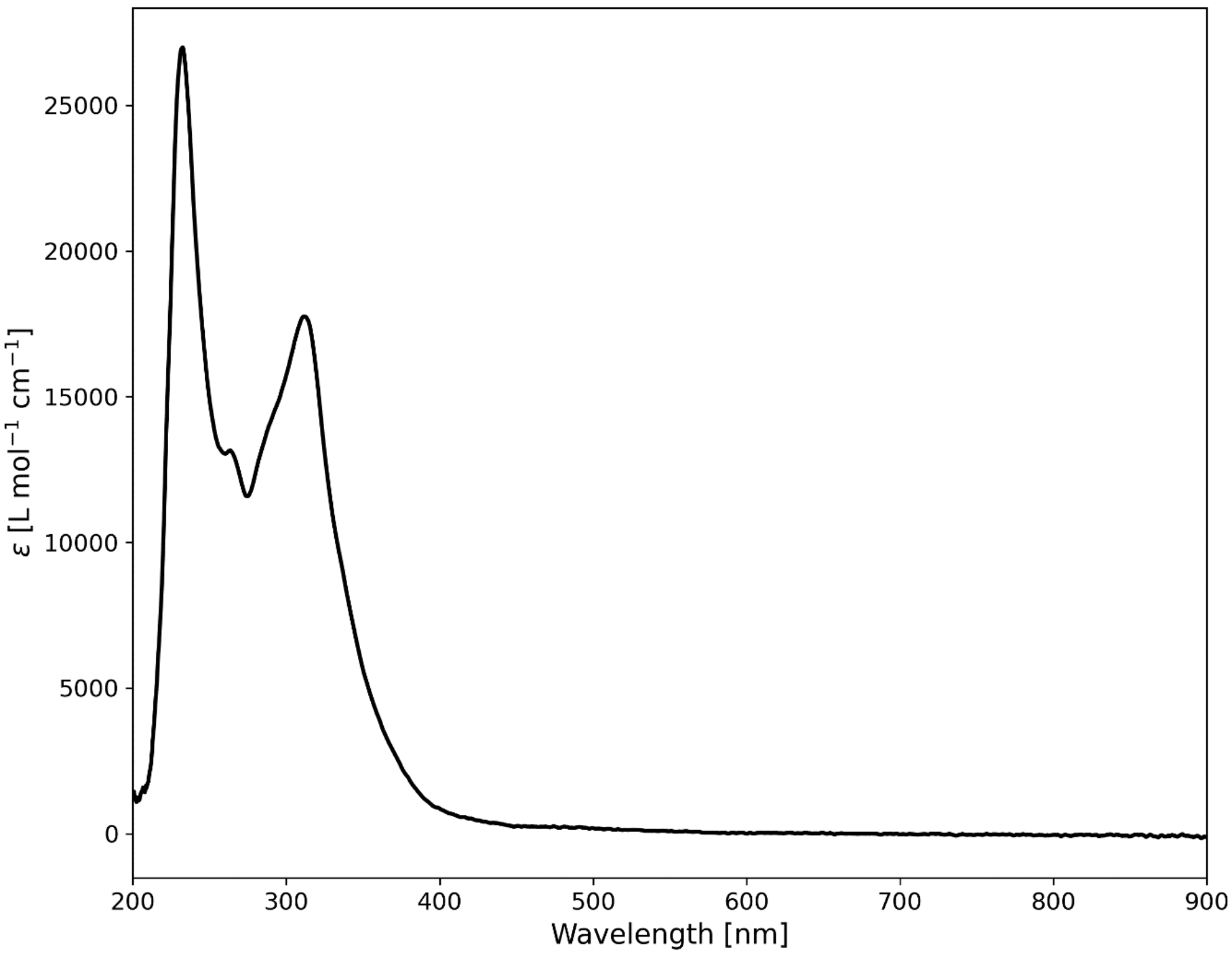

*Figure S 50. UV/Vis spectrum of **3** in THF.*

Computational Details

Gas phase structures were optimized using Gaussian16[14] with the M06-2X[15] functional with Grimme's D3 dispersion correction[16] using the cc-pVTZ[17] basis set. All computations used Gaussian's ultrafine grid. Frequency analyses were performed to confirm the absence of imaginary frequencies for optimized geometries. Optimized geometries and orbitals were visualized using Avogadro;[18] MOs and NBOs are plotted with isosurface values of +/− 0.06. NBO analysis was performed at the same level of theory as the geometry optimizations/frequency analyses, using the NBO 7.0 program.[19] Atoms in molecules (AIM) analysis was performed using Multiwfn 3.8.[20] IR frequencies were corrected by a scaling factor of 0.946.[21]

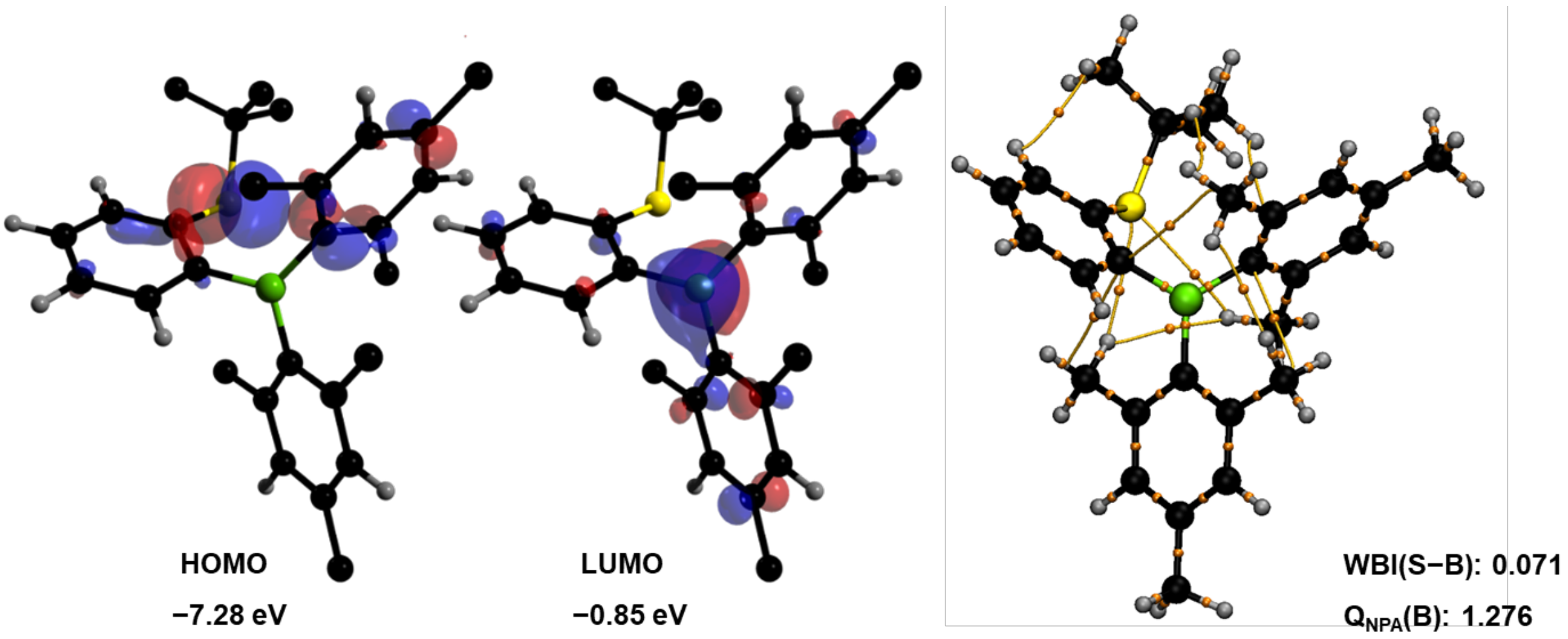

*Figure S 51. Optimized gas-phase geometry with selected DFT-computed molecular orbitals, bond critical paths and points of **3**, Wiberg bond index (WBI) for the S−B interaction, and natural population analysis (NPA) charge for boron.*

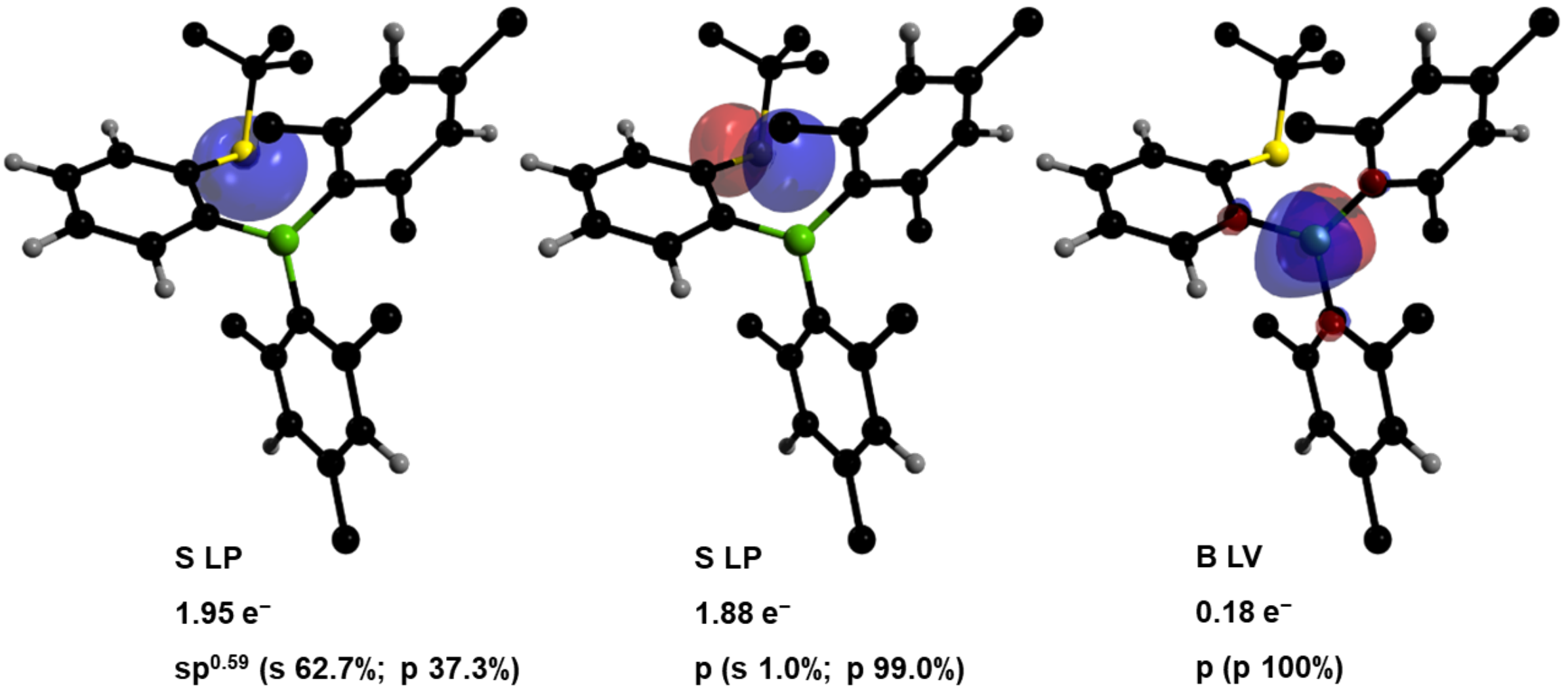

*Figure S 52. Selected NBOs, along with their occupancies and compositions of **3**, showing the two lone pairs (LP) on S and the lone valence (LV) on B.*

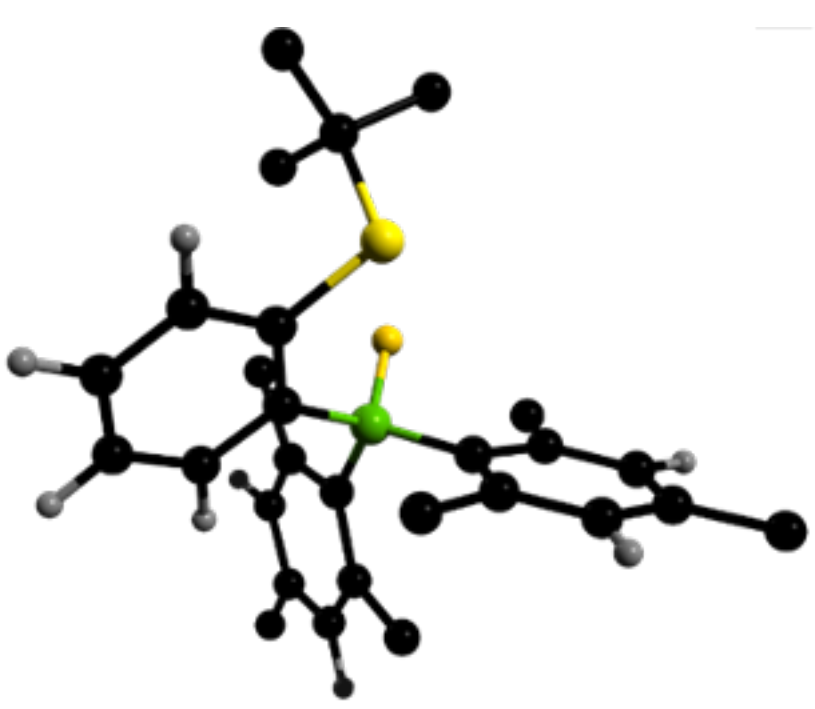

*Figure S 53. Gas-phase optimized geometry of **3F⁻**.*

References

[1] Ilic, S.; Alherz, A.; Musgrave, C. B.; Glusac, K. D. Thermodynamic and kinetic hydricities of metal-free hydrides. *Chemical Society Reviews* **2018**, *47* (8), 2809-2836. DOI: 10.1039/C7CS00171A.
[2] Connelly, N. G.; Geiger, W. E. Chemical Redox Agents for Organometallic Chemistry. *Chemical Reviews* **1996**, *96* (2), 877-910. DOI: 10.1021/cr940053x.
[3] DuBois, D. L.; Berning, D. E. Hydricity of transition-metal hydrides and its role in CO_2 reduction. *Applied Organometallic Chemistry* **2000**, *14* (12), 860−862. DOI: 10.1002/1099-0739(200012)14:12<860::AID-AOC87>3.0.CO;2-A.
[4] Kütt, A.; Tshepelevitsh, S.; Saame, J.; Lõkov, M.; Kaljurand, I.; Selberg, S.; Leito, I. Strengths of Acids in Acetonitrile. *European Journal of Organic Chemistry* **2021**, (9), 1407−1419. DOI: 10.1002/ejoc.202001649.
[5] Avinash, I.; Parveen, S.; Anantharaman, G. Backbone Boron-Functionalized Imidazoles/Imidazoilum Salts: Synthesis, Structure, Metalation Studies, and Fluoride Sensing Properties. *Inorganic Chemistry* **2020**, *59* (8), 5646−5661. DOI: 10.1021/acs.inorgchem.0c00348.
[6] Yakelis, N. A.; Bergman, R. G. Safe Preparation and Purification of Sodium Tetrakis[(3,5-Trifluoromethyl)phenyl]borate ($NaBArF_{24}$): Reliable and Sensitive Analysis of Water in Solutions of Fluorinated Tetraarylborates. Organometallics **2005**, *24* (14), 3579–3581. DOI: 10.1021/om0501428.
[7] Lalancette, J.-M.; Rollin, G.; Dumas, P. Metals Intercalated in Graphite. I. Reduction and Oxidation. *Canadian Journal of Chemistry* **1972**, *50* (18), 3058–3062. DOI: 10.1139/v72-485.
[8] Fanjul-Mosteirín, N.; Jehanno, C.; Ruipérez, F.; Sardon, H.; Dove, A. P. Rational Study of DBU Salts for the CO_2 Insertion into Epoxides for the Synthesis of Cyclic Carbonates. *ACS Sustainable Chemistry & Engineering* **2019**, *7* (12), 10633–10640. DOI: 10.1021/acssuschemeng.9b01300.
[9] Wilken, M.; Ortgies, S.; Breder, A.; Siewert, I. Mechanistic Studies on the Anodic Functionalization of Alkenes Catalyzed by Diselenides. *ACS Catalysis* **2018**, *8* (11), 10901−10912. DOI: 10.1021/acscatal.8b01236.
[10] Sheldrick, G. M.; *SHELXT-2014, Program for Crystal Structure Solution and Refinement*, Universität Göttingen, **2014**.
[11] Sheldrick, G. M. SHELXT – Integrated space-group and crystal-structure determination. *Acta Crystallographica Section A* **2015**, *71* (1), 3−8. DOI: 10.1107/S2053273314026370.
[12] Hübschle, C. B.; Sheldrick G. M.; Dittrich B. ShelXle: a QT graphical user interface for SHELXL. *Journal of Applied Crystallography* **2011**, *44* (6), 1281−1284. DOI: 10.1107/S0021889811043202.
[13] Brandenburg, K. *Diamond 4.6.6.* Crystal Impact GbR: Bonn, Germany **1997–2021**.
[14] Frisch, M. J.; Trucks, G.W.; Schlengel, H.B.; Scuseria, G.E.; Robb, M.A.; Cheeseman, J.R.; Scalmani, G.; Barone, V.; Petersson, G.A.; Nakatsuji, H.; Li, X.; Caricato, M.; Marenich, A.V; Bloino, J.; Janesko, B.G.; Gomperts, R.; Mennucci, B.; Hratchian, H.P.; Ortiz, J.V.; Izmaylov, A.F.; Sonnenberg, J.L.; Williams-Young, D.; Ding, F.; Lipparini, F.; Egidi, F.; Goings, J.; Peng, B.; Petrone, A.; Henderson, T.; Ranasinghe, D.; Zakrzewski, V.G.; Gao, J.; Rega, N.; Zheng, G.; Liang, W.; Hada, M.; Ehara, M.; Toyota, K.; Fukuda, R.; Hasegawa, J.; Ishida, M.; Nakajima, T.; Honda, Y.; Kitao, O.; Nakai, H.; Vreven, T.; Throssell, K.; Montgomery Jr., J.A.;. Peralta, J.E.; Ogliaro, F.; Bearpark, M.J.; Heyd, J.J.; Brothers, E.N.; Kudin, K.N.; Staroverov, V.N.; Keith, T.A.; Kobayashi, R.; Normand, J.; Raghavachari, K.; Rendell, A.P.; Burant, J.C.; Iyengar, S.S.; Tomasi, J.; Cossi, M.; Miliam, J.M.; Klene, M.; Adamo, C.; Cammi, R.; Ochterski, J.W.; Martin, R.L. Morokuma, K.; Farkas, O.; Foresman, J.B.; Fox, D.J. *Gaussian 16,* Revision B.01, Gaussian, Inc.: Wallingford CT, USA **2016**.
[15] Zhao, Y.; Truhlar, D. G. The M06 suite of density functionals for main group thermochemistry, thermochemical kinetics, noncovalent interactions, excited states, and transition elements: two new functionals and systematic testing of four M06-class functionals and 12 other functionals. *Theoretical Chemistry Accounts* **2008**, *120*, 215−241. DOI: 10.1007/s00214-007-0310-x.
[16] Grimme, S.; Antony, J.; Ehrlich, S.; Krieg, H. A consistent and accurate ab initio parametrization of density functional dispersion correction (DFT-D) for the 94 elements H-Pu. *The Journal of Chemical Physics* **2010**, *132* (15), 154104. DOI: 10.1063/1.3382344.
[17] Dunning Jr., T. H. Gaussian basis sets for use in correlated molecular calculations. I. The atoms boron through neon and hydrogen*.* *The Journal of Chemical Physics* **1989**, *90* (2), 1007−1023. DOI: 10.1063/1.456153.
[18] Hanwell, M. D.; Curtis, D. E.; Lonie, D. C.; Vandermeersch, T.; Zurek, E.; Hutchison, G. R. Avogadro: an advanced semantic chemical editor, visualization, and analysis platform. *Journal of Cheminformatics* **2012**, *4* (1), 17. DOI: 10.1186/1758-2946-4-17.
[19] Glendening, E. D.; Landis, C. R.; Weinhold, F. *NBO 7.0*: New vistas in localized and delocalized chemical bonding theory. *Journal of Computational Chemistry* **2019**, *40* (25), 2234−2241. DOI: 10.1002/jcc.25873.
[20] Lu, T.; Chen, F. Multiwfn: a multifunctional wavefunction analyzer. *Journal of Computational Chemistry* **2012**, *33* (5), 580–592. DOI: 10.1002/jcc.22885.
[21] Alecu, I. M.; Zheng, J.; Zhao, Y.; Truhlar, D. G. Computational Thermochemistry: Scale Factor Databases and Scale Factors for Vibrational Frequencies Obtained from Electronic Model Chemistries. *Journal of Chemical Theory and Computation* **2010**, *6* (9), 2872–2887. DOI: 10.1021/ct100326h.
